# Randomized open-label trial of semaglutide and dapagliflozin in patients with type 2 diabetes of different pathophysiology

**DOI:** 10.1038/s42255-023-00943-3

**Published:** 2024-01-04

**Authors:** Chinmay Dwibedi, Ola Ekström, Jasmine Brandt, Martin Adiels, Stefan Franzén, Birgitta Abrahamsson, Anders H. Rosengren

**Affiliations:** 1 Department of Neuroscience and Physiology, Sahlgrenska Academy at the University of Gothenburg, Gothenburg, Sweden; 2 Institute of Medicine, Sahlgrenska Academy at the University of Gothenburg, Gothenburg, Sweden; 3 Department of Clinical Sciences, Diabetes and Endocrinology, Lund University, Malmö, Sweden; 4 Department of Clinical Chemistry and Pharmacology, Skåne University Hospital, Lund, Sweden; 5 Clinical Studies Sweden, Forum South, Skåne University Hospital, Lund, Sweden; 6 AstraZeneca, Gothenburg, Sweden

**Keywords:** Type 2 diabetes, Randomized controlled trials, Type 2 diabetes

## Abstract

The limited understanding of the heterogeneity in the treatment response to antidiabetic drugs contributes to metabolic deterioration and cardiovascular complications^1,2^, stressing the need for more personalized treatment^1^. Although recent attempts have been made to classify diabetes into subgroups, the utility of such stratification in predicting treatment response is unknown^3^. We enrolled participants with type 2 diabetes (*n* = 239, 74 women and 165 men) and features of severe insulin-deficient diabetes (SIDD) or severe insulin-resistant diabetes (SIRD). Participants were randomly assigned to treatment with the glucagon-like peptide 1 receptor agonist semaglutide or the sodium–glucose cotransporter 2 inhibitor dapagliflozin for 6 months (open label). The primary endpoint was the change in glycated haemoglobin (HbA1c). Semaglutide induced a larger reduction in HbA1c levels than dapagliflozin (mean difference, 8.2 mmol mol^−1^; 95% confidence interval, −10.0 to −6.3 mmol mol^−1^), with a pronounced effect in those with SIDD. No difference in adverse events was observed between participants with SIDD and those with SIRD. Analysis of secondary endpoints showed greater reductions in fasting and postprandial glucose concentrations in response to semaglutide in participants with SIDD than in those with SIRD and a more pronounced effect on postprandial glucose by dapagliflozin in participants with SIDD than in those with SIRD. However, no significant interaction was found between drug assignment and the SIDD or SIRD subgroup. In contrast, continuous measures of body mass index, blood pressure, insulin secretion and insulin resistance were useful in identifying those likely to have the largest improvements in glycaemic control and cardiovascular risk factors by adding semaglutide or dapagliflozin. Thus, systematic evaluation of continuous pathophysiological variables can guide the prediction of the treatment response to these drugs and provide more information than stratified subgroups (NCT04451837).

## Abstract (web summary)

Systematic evaluation of glucose control, body mass index, blood pressure, insulin secretion and insulin resistance is leveraged to identify patients who are likely to receive the greatest metabolic benefit from common antidiabetic drugs.

## Main

Type 2 diabetes is an escalating health problem of enormous proportions^1^. International guidelines highlight the need for more personalized treatment^1^, but the concept has not yet been systematically examined in randomized trials specifically designed to evaluate treatment responses in patients with different characteristics. This is important to reduce the risk of biases compared with observational studies, meta-analyses or post hoc analyses of previously conducted trials^1,2^.

The choice of antihyperglycaemic treatment is usually based on comorbidities, baseline cardiovascular risk, side effects, cost and clinical assumptions, but rarely on measurements of pathophysiological features driving the deteriorating metabolic state that ultimately leads to complications^2^. Furthermore, evaluations of glucose-lowering drugs have mainly been based on average efficacy data, and there is a major gap in our understanding of treatment response heterogeneity^1^. To address the current knowledge gaps and facilitate the cost-effective use of drugs, the latest international guidelines emphasize the need to investigate treatment efficacy in different subgroups of patients^1^.

Interestingly, a recent analysis of 9,000 patients with diabetes highlighted five clusters, each with different characteristics and risk of complications^3^. Two of these clusters are particularly aggressive. One cluster has been coined ‘severe insulin-deficient diabetes’ (SIDD), which features young age at onset, low body mass index (BMI) and poor insulin secretion. The second cluster, termed ‘severe insulin-resistant diabetes’ (SIRD), presents at older age and is associated with high BMI and high insulin resistance. Similar clusters have been reproduced in several multiethnic cohorts^4–10^.

This could potentially provide a tool to distinguish individuals with different pathophysiology. However, the clinical relevance of such stratification for predicting treatment response has also been questioned, as it assumes homogeneity within each cluster^8,11–14^. An alternative option to stratifying patients into subgroups would be using continuous variables that reflect individual pathophysiology^8,15–19^. Evaluating the most feasible approaches to predict the individual response to common drugs is critical to guiding future clinical and scientific work in precision medicine.

In this trial, patients with SIDD or SIRD characteristics were randomly assigned to receive semaglutide, a glucagon-like peptide 1 receptor agonist (GLP1ra), or dapagliflozin, a sodium–glucose cotransporter 2 inhibitor (SGLT2i). GLP1ra and SGLT2i drugs are increasingly used and have shown cardiovascular benefits in patients with established cardiovascular or renal disease. However, for most patients with type 2 diabetes, it is currently unclear who benefit most from these drugs. In particular, it is unknown to what extent the glucose-lowering efficacy depends on the pathophysiological characteristics of the patient. The trial represents the first randomized comparison of a GLP1ra and an SGLT2i in stratified subgroups, allowing for side-to-side comparisons of the efficacy of these two drug classes in patients with different pathophysiology. We aimed to address two main questions of clinical and scientific importance: (1) whether knowledge of the SIDD or SIRD subgroup could help inform the decision of adding semaglutide or dapagliflozin to metformin in terms of metabolic benefits and (2) whether continuous pathophysiological measures could be used to identify which patients are likely to benefit most from these drugs in terms of metabolic improvement.

**Extended Data Fig. 1:**
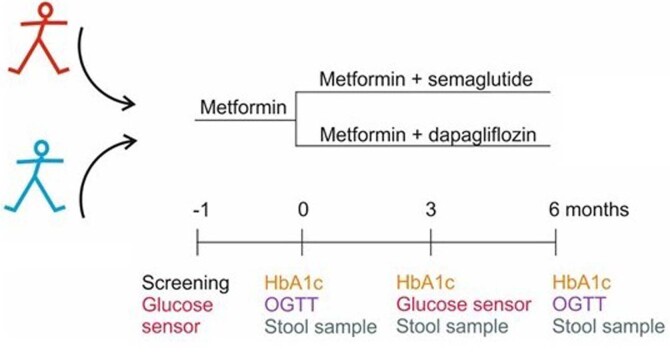
Design of study. Figure depicts the different study visits and allocation of randomized treatment. Source data

A total of 360 patients with type 2 diabetes were screened, with 239 randomized to receive semaglutide or dapagliflozin in addition to metformin (the study was approved by the Swedish Ethical Review Authority (2020-01353); Extended Data Fig. 1). The study participants had either SIDD (*n* = 126) or SIRD (*n* = 113) characteristics (Table 1).

**Table 1:** Demographic and baseline characteristics of study participants

Characteristics	SIDD (*n* = 126)			SIRD (*n* = 113)			All (*n* = 239)
	Semaglutide (*n* = 63)	Dapagliflozin (*n* = 63)	All SIDD	Semaglutide (*n* = 57)	Dapagliflozin (*n* = 56)	All SIRD	
HbA1c (mmol mol^−1^)	57.4 ± 9.9	57.5 ± 11.0	57.5 ± 10.4	54 ± 7.7	53 ± 7.6	53.4 ± 7.7	55.5 ± 9.4
Age (years)	63 ± 10	63 ± 10	63 ± 10	69 ± 9	68 ± 7	69 ± 8	66 ± 10
Male sex, *n* (%)	45 (71.4)	46 (73.0)	91 (72.2)	34 (59.7)	40 (71.4)	74 (65.5)	165 (69.0)
BMI (kg m^−2^)^a^	28.3 ± 4.1	29.1 ± 5.1	28.8 ± 4.7	34.3 ± 4.4	33.8 ± 5.9	34.1 ± 5.3	31.2 ± 5.6
Waist circumference (cm)	104 ± 11	105 ± 13	104.7 ± 12	119 ± 12	117 ± 15	118 ± 13	111 ± 14
Diabetes duration (years)	5.2 ± 3.1	4.7 ± 4.3	5.0 ± 3.8	4.4 ± 3.3	3.8 ± 3.0	4.1 ± 3.2	4.6 ± 3.5
Systolic blood pressure (mm Hg)	127 ± 13	130 ± 13	129 ± 13	132 ± 13	129 ± 12	131 ± 12	130 ± 13
Diastolic blood pressure (mm Hg)	76 ± 11	79 ± 8	78 ± 9	77 ± 10	77 ± 9	77 ± 9	77 ± 9
LDL (mmol l^−1^)	2.2 ± 0.8	2.6 ± 0.7	2.4 ± 0.8	2.3 ± 1.1	2.3 ± 0.8	2.3 ± 0.9	2.3 ± 0.9
Triglycerides (mmol l^−1^)	1.7 ± 1.1	1.5 ± 0.6	1.6 ± 0.8	2.1 ± 1.1	2.1 ± 0.9	2.1 ± 1	1.8 ± 1.0
eGFR (ml min^−1^ 1.73 m^−2^)	75.2 ± 13.4	78.3 ± 11.3	76.8 ± 12.4	66.8 ± 11.2	68.8 ± 11.4	67.8 ± 11.3	72.5 ± 12.7
Urinary albumin/creatinine index (g mol^−1^)	1.8 ± 4.7	1 ± 1.6	1.4 ± 3.6	1.6 ± 2.8	1.7 ± 2.8	1.6 ± 2.8	1.5 ± 3.2
Fasting glucose (mmol l^−1^)	9.1 ± 2.4	8.6 ± 2.4	8.8 ± 2.4	7.8 ± 1.7	7.8 ± 2	7.8 ± 1.8	8.3 ± 2.2
Glucose 120 min (mmol l^−1^)^b^	16.1 ± 3.8	15.8 ± 3.6	15.9 ± 3.7	14.3 ± 3.3	14.1 ± 3.5	14.2 ± 3.4	15.1 ± 3.7
HOMA2-B	67.6 ± 39.6	67.3 ± 28.4	67.5 ± 33.7	117.8 ± 39.4	127.2 ± 70.4	122.7 ± 57.5	91.9 ± 53.3
HOMA2-IR	2.7 ± 1.1	2.6 ± 1.0	2.6 ± 1.1	4.2 ± 1.3	4.2 ± 1.1	4.2 ± 1.2	3.3 ± 1.4
Insulin sensitivity index	2.8 ± 1.5	3.8 ± 2.0	3.3 ± 1.8	1.5 ± 0.7	1.7 ± 0.8	1.6 ± 0.8	2.6 ± 1.7
Disposition index	105 ± 93	143 ± 140	126 ± 122	158 ± 125	186 ± 215	173 ± 179	147 ± 151
Time in range^c^	0.71 ± 0.29	0.74 ± 0.23	0.70 ± 0.30	0.83 ± 0.19	0.80 ± 0.23	0.82 ± 0.21	0.76 ± 0.24
Average glucose (mmol l^−1^)^c^	8.6 ± 2.5	8.5 ± 2.0	8.5 ± 2.2	7.4 ± 1.8	7.5 ± 2.1	7.5 ± 2.0	8.1 ± 2.2
Coefficient of variance of glucose^c^	0.25 ± 0.07	0.24 ± 0.05	0.25 ± 0.06	0.21 ± 0.05	0.23 ± 0.05	0.22 ± 0.05	0.23 ± 0.05
NAFLD liver fat score	0.6 ± 1.3	0.6 ± 1.6	0.6 ± 1.5	2.8 ± 2.6	3.0 ± 4.6	3.0 ± 3.7	1.8 ± 3.0

Plus–minus values are mean ± s.d.

^a^BMI is the weight in kilograms divided by the square of the height in metres.

^b^Glucose 120 min refers to the glucose concentration at 120 min of an OGTT.

^c^Time in range, average glucose and coefficient of variance of glucose were obtained from continuous glucose monitoring for 2 weeks. Time in range refers to the fraction of time with glucose between 3.9 and 10.0 mmol l^−1^.

**Fig. 1:**
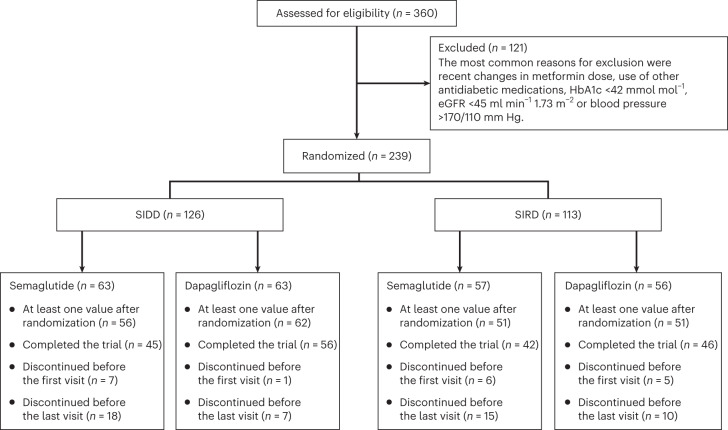
Study profile shown as a CONSORT diagram. Participants with at least one value after randomization were included in the full analysis set. The most common reason for study discontinuation was the occurrence of side effects from any of the drugs. Two participants inadvertently changed the metformin dose during the study; one participant underwent surgery for coronary artery bypass graft; one participant underwent gastric sleeve surgery; one participant stopped the study medication because of a misunderstanding; and one participant was lost to follow-up. Source data

Of the 239 randomized participants, 220 (67 women, 153 men) had at least one glycated haemoglobin (HbA1c) measurement after randomization and were included in the full analysis set independent of compliance (Fig. 1 and Supplementary Tables 1 and 2). Participants on semaglutide reported mainly gastrointestinal adverse events, which required dose reduction from 1.0 to 0.5 mg in 12 participants. Those who needed dose reduction were younger than those who remained at full dose (mean age difference, 6.8 years; 95% confidence interval (CI), 0.7 to 12.9 years). Participants on dapagliflozin experienced more urinary tract symptoms than those on semaglutide, with a higher incidence in the SIRD group than in the SIDD group (Supplementary Table 3).

**Fig. 2:**
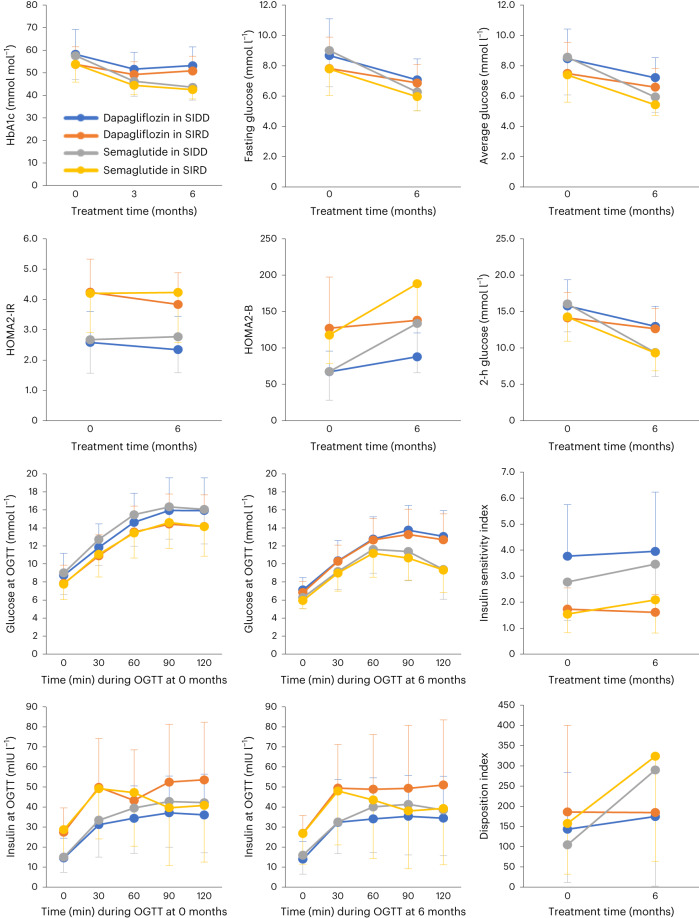
Changes over time in fasting and postprandial measures. Data show means and s.d. of fasting and postprandial measures in response to each study drug in participants with SIDD and SIRD. Measurements were obtained at baseline (0) and after 6 months of treatment (HbA1c was also measured at 3 months). Statistics for comparisons of the primary and secondary variables are presented in Table 2 and Supplementary Table 4. Average glucose measures were obtained from continuous glucose monitoring. Glucose and insulin excursions at 0, 30, 60, 90 and 120 min of the OGTT at baseline (0 months) and after 6 months are also shown, as well as the disposition index and insulin sensitivity index derived from the OGTT. Data represent *n* = 57 for dapagliflozin treatment in the SIDD group (16 women, 41 men), indicated by blue lines; *n* = 47 for dapagliflozin treatment in the SIRD group (14 women, 33 men), indicated by orange lines; *n* = 49 for semaglutide treatment in the SIDD group (14 women, 35 men), indicated by grey lines; and *n* = 44 for semaglutide treatment in the SIRD group (16 women, 28 men), indicated by yellow lines. Numbers include participants who underwent blood sampling at both baseline and 6 months. The last semaglutide dose was administered 3 days before the final OGTT and the last dapagliflozin dose was administered 12 h before the final OGTT to provide an average steady-state condition for both drugs with regard to their different half-lives. Source data

We first analysed which of the study drugs resulted in the greatest improvement in HbA1c (Fig. 2 and Table 2). Participants receiving semaglutide had a larger average reduction in HbA1c levels than those receiving dapagliflozin, with a mean difference of 8.2 mmol mol^−1^ (95% CI, −10.0 to −6.3 mmol mol^−1^) between the randomization groups. Similar differences were observed when participants with SIDD and SIRD were analysed separately (8.6 mmol mol^−1^ (95% CI, −11.3 to −6.0 mmol mol^−1^) in the SIDD group and 7.8 mmol mol^−1^ (95% CI, −10.3 to −5.3 mmol mol^−1^) in the SIRD group).

**Table 2:** Effect of study drugs on primary and secondary endpoints

Endpoints	SIDD (*n* = 118, 32 women and 86 men)			SIRD (*n* = 102, 35 women and 67 men)			Interaction *P* value^a^
	Semaglutide (*n* = 56)	Dapagliflozin (*n* = 62)	Mean difference (95% CI)^a^	Semaglutide (*n* = 51)	Dapagliflozin (*n* = 51)	Mean difference (95% CI)	
Primary endpoint							
Change in HbA1c (mmol mol^−1^)	−13.4	−4.7	−8.6 (−11.3 to −6.0)	−10.5	−2.7	−7.8 (−10.3 to −5.3)	0.7
Secondary endpoints							
Change in fasting glucose (mmol l^−1^)	−2.7	−1.6	−1.1 (−1.9 to −0.4)	−1.9	−1.0	−0.9 (−1.5 to −0.2)	0.6
Change in glucose at 120 min (mmol l^−1^)	−6.7	−2.8	−3.9 (−4.9 to −3.0)	−5.0	−1.5	−3.5 (−4.6 to −2.5)	0.6
Change in HOMA2-B	66.2	20.6	45.5 (31.0 to 60.0)	70.5	10.8	59.7 (38.7 to 80.6)	0.3
Change in HOMA2-IR	0.1	−0.2	0.33 (0.04 to 0.63)	0.1	−0.4	0.43 (0.01 to 0.84)	0.7
Change in disposition index	185	31	154 (77 to 230)	166	0	167 (67 to 267)	0.8
Change in insulin sensitivity index	0.7	0.2	0.44 (−0.04 to 0.92)	0.5	−0.1	0.67 (0.35 to 0.98)	0.5
Change in time in range^b^	0.22	0.11	0.11 (0.00 to 0.22)	0.03	0.07	−0.04 (−0.16 to 0.09)	0.07
Change in average glucose (mmol l^−1^)	−2.9	−1.3	−1.6 (−2.5 to −0.7)	−1.8	−1.0	−0.9 (−1.6 to −0.1)	0.2
Change in coefficient of variance of glucose	−0.02	0.02	−0.04 (−0.06 to −0.02)	−0.02	0.02	−0.03 (−0.06 to −0.01)	0.6
Change in BMI (kg m^−2^)	−1.9	−1.1	−0.8 (−1.2 to −0.5)	−2.4	−1.3	−1.1 (−1.6 to −0.6)	0.3
Change in waist circumference (cm)	−5.8	−4.1	−1.7 (−3.4 to −0.1)	−5.4	−4.4	−1.1 (−2.7 to 0.5)	0.6
Change in systolic blood pressure (mm Hg)	−3	−3	0 (−4 to 3)	−6	0	−5 (−10 to 0)	0.1
Change in diastolic blood pressure (mm Hg)	2	−2	4 (1 to 7)	0	0	0 (−3 to 0)	0.08
Change in LDL (mmol l^−1^)	−0.3	0.0	−0.2 (−0.4 to −0.1)	−0.3	0.1	−0.4 (−0.6 to −0.1)	0.4
Change in triglycerides (mmol l^−1^)	−0.2	0.0	−0.2 (−0.5 to 0.0)	−0.3	0.1	−0.4 (−0.7 to −0.1)	0.4
Change in eGFR (ml min^−1^ 1.73 m^−2^)	−0.6	−3.9	3.3 (0.8 to 5.7)	0.0	−2.2	2.2 (0.2 to 4.2)	0.5
Change in eGFR relative to baseline (%)^c^	−0.4	−5.1	4.7 (1.3 to 8.2)	0.4	−3.1	3.4 (0.4 to 6.5)	0.6
Change in urine albumin/creatinine index (g mol^−1^)	−0.3	0.0	−0.3 (−0.9 to 0.3)	−0.4	0.3	−0.7 (−1.7 to 0.4)	0.5
Change in NAFLD liver fat score	0.2	−0.2	0.4 (−0.1 to 0.9)	−0.6	−0.8	0.8 (−1.4 to 2.0)	0.9
Change in HbA1c in the per-protocol set (mmol mol^−1^)^d^	−14.3	−5.1	−9.2 (−12.2 to −6.3)	−11.6	−3.2	−8.4 (−11.1 to −5.6)	0.7

Data show the changes relative to baseline in the primary and secondary endpoints in response to semaglutide and dapagliflozin in the full analysis set (*n* = 220) of participants with SIDD or SIRD.

^a^Estimated mean differences in values (response to semaglutide minus response to dapagliflozin) are presented with 95% CIs. The *P* value for the treatment-by-subgroup interaction term (two-sided, unadjusted for multiple comparisons) was analysed using an analysis of covariance model.

^b^Time in range (fraction of time with glucose between 3.9 and 10.0 mmol l^−1^), average glucose and coefficient of variance of glucose were obtained from continuous glucose monitoring.

^c^Reduction in eGFR is presented as the percentage change relative to baseline.

^d^Change in HbA1c in the subset of participants treated according to the protocol, with full doses and complete study visits (*n* = 178, 54 women and 124 men), instead of the full analysis set.

We then addressed whether knowledge of the subgroup provides information on the clinical response (Supplementary Table 4). In participants with SIDD characteristics, the average HbA1c decreased by 13.4 mmol mol^−1^ in response to semaglutide, as compared with 10.5 mmol mol^−1^ in participants with SIRD characteristics. The mean difference between the subgroups was 2.9 mmol mol^−1^ (95% CI, −5.7 to −0.1 mmol mol^−1^; *P* = 0.04). In those assigned to dapagliflozin treatment, the average reduction in HbA1c in the SIDD group was 4.7 mmol mol^−1^, as compared with 2.7 mmol mol^−1^ in the SIRD group, for a mean difference of 2.0 mmol mol^−1^ (95% CI, −4.4 to 0.3 mmol mol^−1^; *P* = 0.09). When treatment and subgroup were evaluated as an interaction term in the statistical model, no statistically significant interaction was observed between drug assignment and SIDD or SIRD characteristics (*P* = 0.7 and model *R*^2^ = 0.28; *P* = 0.4 after correcting for baseline HbA1c and metformin dose; *P* = 0.2 when only participants with HbA1c >53 mmol mol^−1^ were analysed). Thus, there was no interaction between the subgroup and the effect of semaglutide or dapagliflozin on glycaemic improvement (Table 2). Participants treated according to the protocol (*n* = 178, with full doses and complete visits, as compared with *n* = 220 in the full analysis set) had similar mean differences but wider CIs (Table 2 and Supplementary Table 4).

**Extended Data Fig. 2:**
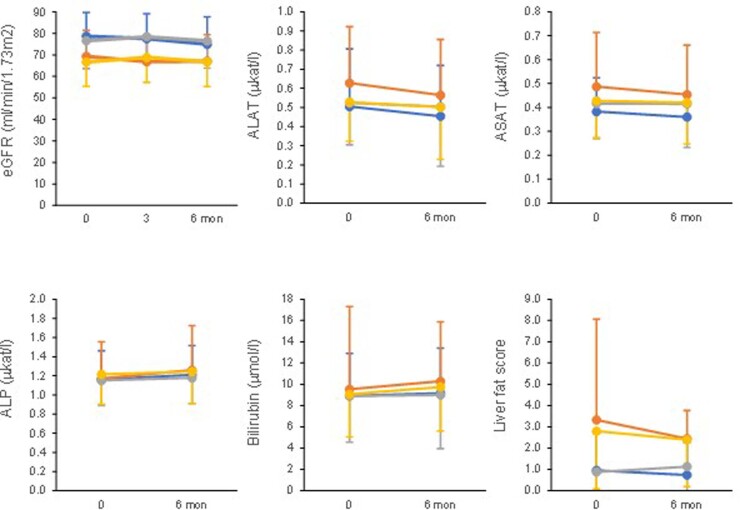
Changes over time of estimated glomerular filtration rate and liver parameters. Data show means and SD of estimated glomerular filtration rate (eGFR), alanine aminotransferase (ALAT), aspartate aminotransferase (ASAT), alkaline phosphatase (ALP), bilirubin and NAFLD liver fat score in response to each study drug in SIDD and SIRD participants, respectively, obtained at baseline (0) and after 6 months of treatment (eGFR was also measured at 3 months). Data represent n = 57 dapagliflozin in SIDD [16 women, 41 men]; n = 47 dapagliflozin in SIRD [14 women, 33 men]; n = 49 semaglutide in SIDD [14 women, 35 men]; n = 44 semaglutide in SIRD [16 women, 28 men]; numbers include participants who underwent blood sampling both at baseline and at 6 months. Source data

Analysis of secondary variables showed that participants on semaglutide had larger improvements in fasting and postprandial glucose, insulin secretion and BMI than those on dapagliflozin (Table 2). In contrast, dapagliflozin treatment improved the homoeostasis model assessment 2 estimate of insulin resistance (HOMA2-IR) compared with semaglutide treatment. When the effects disaggregated by clusters were analysed, semaglutide was found to result in greater reductions in fasting glucose, glucose at 2 h of the oral glucose tolerance test (OGTT) and average glucose concentration during continuous monitoring, as well as a higher time in range in participants with SIDD than in those with SIRD (Table 2, Fig. 2 and Supplementary Table 4). A tendency for improved nonalcoholic fatty liver disease (NAFLD) liver fat score in the SIRD group compared with the SIDD group in response to semaglutide was also observed (Extended Data Fig. 2 and Supplementary Table 4). Meanwhile, dapagliflozin resulted in a significantly larger improvement in 2-h glucose and a tendency for greater improvements in fasting glucose and HOMA2-IR in participants with SIDD than in those with SIRD. We also observed that semaglutide resulted in a larger improvement in patient-reported treatment satisfaction^19^ compared with dapagliflozin, particularly in the SIDD group. Moreover, after treatment with semaglutide, participants with SIRD reported less hunger, less difficulty in resisting food cravings and less cravings for starchy foods as compared with participants with SIDD (Supplementary Tables 5 and 6).

As the study allows for randomized comparisons of a GLP1ra and an SGLT2i in subgroups and includes OGTT data, we contrasted the effects of the study compounds on fasting and postprandial measures. Although semaglutide resulted in overall greater glycaemic improvements, it is notable that the effect of dapagliflozin relative to semaglutide was larger on fasting than postprandial outcomes. The effect of dapagliflozin on fasting glucose was 57% relative to that of semaglutide, whereas the effect of dapagliflozin on 2-h glucose was only 38% compared with that of semaglutide (Table 2 and Supplementary Table 4). In parallel, dapagliflozin improved the HOMA2 estimate of beta-cell function (HOMA2-B, reflecting insulin secretion in the fasted state) by 24% relative to semaglutide, with a more pronounced effect in participants with SIDD, whereas the effect of dapagliflozin on the disposition index (reflecting postprandial insulin secretion) was merely 10% compared with that of semaglutide. We further observed that HOMA2-IR (insulin resistance in the fasted state) was improved by dapagliflozin, particularly in participants with SIRD; however, it was unaffected by semaglutide. In contrast, semaglutide had a significant effect on the insulin sensitivity index, which estimates postprandial insulin sensitivity.

As the cohort included patients with a baseline HbA1c between 42 and 91 mmol mol^−1^, we wanted to compare those with HbA1c <53 mmol mol^−1^ (‘well-regulated’, *n* = 123) and >53 mmol mol^−1^ (‘dysregulated’, *n* = 116) at randomization. This is relevant because current guidelines emphasize the potential value of intensifying treatment even in patients with lower glycaemic levels^1,17^. Clinical data revealed that beta-cell function had partly recovered from diagnosis to study inclusion in participants with well-regulated SIDD but remained low in those who were dysregulated. The addition of semaglutide resulted in a relatively larger improvement in HOMA2-B as compared with dapagliflozin, whereas dapagliflozin resulted in a greater improvement in HOMA2-IR (Supplementary Tables 7–9).

In contrast, individuals with SIRD who were dysregulated at randomization had presented with already high HOMA2-IR at diagnosis and showed a marked decrease in HOMA2-B between diagnosis and study inclusion, indicating that metformin treatment had been insufficient. During the study, these participants had clear improvements in HbA1c, BMI and HOMA2-B in response to both drugs, with a relatively larger effect of semaglutide. Notably, well-regulated participants had improved HOMA2-B in response to both drugs (Supplementary Table 9), at a similar magnitude to that observed in dysregulated participants. This demonstrates that beta-cell function is ameliorated in response to GLP1ra and SGLT2i drugs even in those with HbA1c <53 mmol mol^−1^.

We next turned to the clinical question of whether continuous pathophysiological measures rather than fixed subgroups could be used to identify which patients are likely to benefit most from these common drugs. First, we analysed whether any of the measures used for cluster designation (HbA1c, BMI, HOMA2-B, HOMA2-IR, glutamic acid decarboxylase (GAD) antibodies and age) explain more of the variation in the change in HbA1c than the clusters (Supplementary Table 10). The full analysis set was included independent of the subgroup. A significant interaction was observed between drug assignment and baseline HbA1c (*P* = 0.002, model *R*^2^ = 0.65), with a steeper association between baseline HbA1c and the glycaemic response to semaglutide (beta coefficient, −0.63) than to dapagliflozin (beta coefficient, −0.41). We also observed a significant interaction between drug assignment and baseline HOMA2-B (*P* = 0.02, *R*^2^ = 0.42), with a more pronounced association between baseline HOMA2-B and the change in HbA1c in response to semaglutide (beta coefficient, 0.08) than to dapagliflozin (beta coefficient, 0.04). When participants were categorized into tertiles of baseline HbA1c or HOMA2-B instead of using continuous data, we observed a significant treatment-by-subgroup interaction for tertiles of baseline HbA1c (*P* = 0.03, *R*^2^ = 0.55). In contrast, the treatment-by-subgroup interaction for tertiles of HOMA2-B did not reach statistical significance (*P* = 0.4, *R*^2^ = 0.46). This suggests that continuous measures of baseline HbA1c and HOMA2-B or categorical tertiles of baseline HbA1c explain more of the change in HbA1c in response to the drugs than the subgroups (for which the interaction with medication was nonsignificant with a model *R*^2^ of 0.28).

Antihyperglycaemic drugs are expected to have larger effects at high HbA1c levels. However, estimating the likely response to different drugs in patients at a given HbA1c level often has clinical relevance. Therefore, we examined the interaction between medication and baseline variables after adjusting for baseline HbA1c. A significant interaction was observed between drug assignment and fasting glucose (*P* = 0.02) and time in range (*P* = 0.02) in determining the glycaemic response when the model was adjusted for baseline HbA1c. A corresponding tendency was observed for the interaction between drug assignment and measures of insulin secretion (*P* = 0.08 for HOMA2-B and *P* = 0.05 for disposition index, adjusted for baseline HbA1c). As participants treated with semaglutide would, in most cases, have a larger glycaemic improvement in absolute terms than those treated with dapagliflozin, we also used the normalized HbA1c response based on *z* scores within each treatment group to compare the relative efficacy of the drugs. This showed that semaglutide had a relatively greater effect than dapagliflozin in terms of *z* scores in participants with high fasting and postprandial glucose, low time in range and poor insulin secretion. In contrast, the effect of dapagliflozin was relatively more pronounced in those with low BMI, high time in range and high insulin secretion.

**Extended Data Fig. 3:**
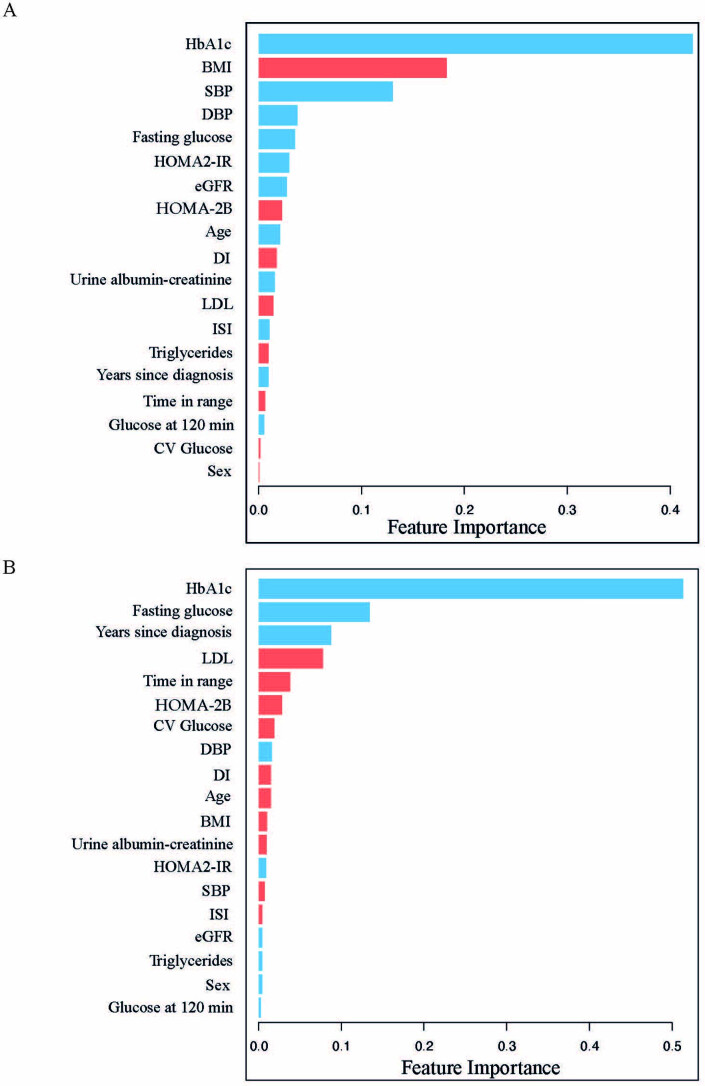
Relative importance of continuous baseline variables in predicting the change in HbA1c. Panels A and B show the relative importance of baseline variables (features) in predicting the change in HbA1c after treatment with dapagliflozin (n = 113 [31 women, 82 men]; panel A) or semaglutide (n = 107 [36 women, 71 men]; panel B). The full analysis set was included in these analyses, independent of SIDD or SIRD cluster, to investigate the continuous baseline variables across all individuals. The measures of relative feature importance sum up to 1 and were generated by XGBoost. The XGBoost algorithm per se is not intended to be used in routine clinical work but was employed here to identify continuous baseline variables that predict the change in HbA1c and which can easily be evaluated by clinicians to provide a better overall assessment of the likely treatment response (such that for example a patient with high BMI or low insulin secretion will likely have a more pronounced response to a particular drug) in conjunction with other clinical data and the general patient context. Blue bars indicate greater reduction in HbA1c with higher values of the baseline trait; red indicates greater reduction in HbA1c with lower values of the baseline trait (lower BMI is for example associated with greater improvement of HbA1c in response to dapagliflozin). Blue indication for the sex term implies greater reduction in women and red indicates greater reduction in men. BMI is body mass index; SBP systolic blood pressure; DBP diastolic blood pressure; eGFR estimated glomerular filtration rate; DI disposition index; ISI insulin sensitivity index; glucose at 120 min refers to glucose measured at 120 minutes. Time in range (the fraction of time with glucose between 3.9 and 10.0 mmol/l) and CV glucose (coefficient of variance of glucose) were obtained from continuous glucose monitoring during two weeks prior to treatment allocation. Source data

**Extended Data Fig. 4:**
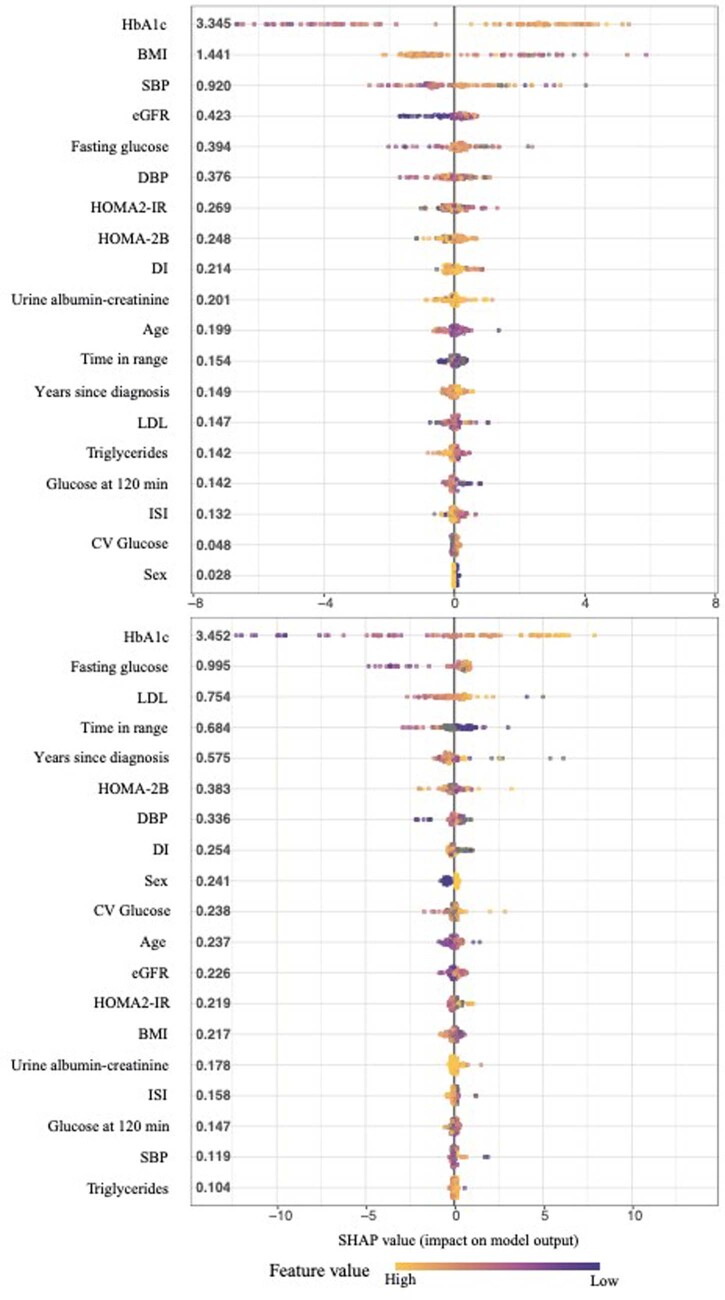
SHAP summary plots for the impact of baseline variables in predicting the change in HbA1c in response to dapagliflozin (upper panel) or semaglutide (lower panel). Each point in the figure corresponds to a participant, and the SHAP value reflects the impact of the baseline variable in predicting the change in HbA1c for that individual. For example, in the row corresponding to HbA1c in the upper panel, all individuals receiving dapagliflozin are plotted in accordance with how much baseline HbA1c predicts the change in HbA1c, and similar for BMI, SBP etc. Thus, for some participants, HbA1c may be a strong predictor and BMI less important, while for other individuals BMI may be the most important predictor. The variables are therefore arranged based on the mean of the absolute SHAP values for each variable. Upper panel shows data for dapagliflozin (n = 113 [31 women, 82 men]) and lower panel shows semaglutide (n = 107 [36 women, 71 men]). SBP denotes systolic blood pressure, DBP diastolic blood pressure, DI disposition index, ISI insulin sensitivity index, and CV glucose denotes coefficient of variation of glucose during continuous monitoring. Source data

**Extended Data Fig. 5:**
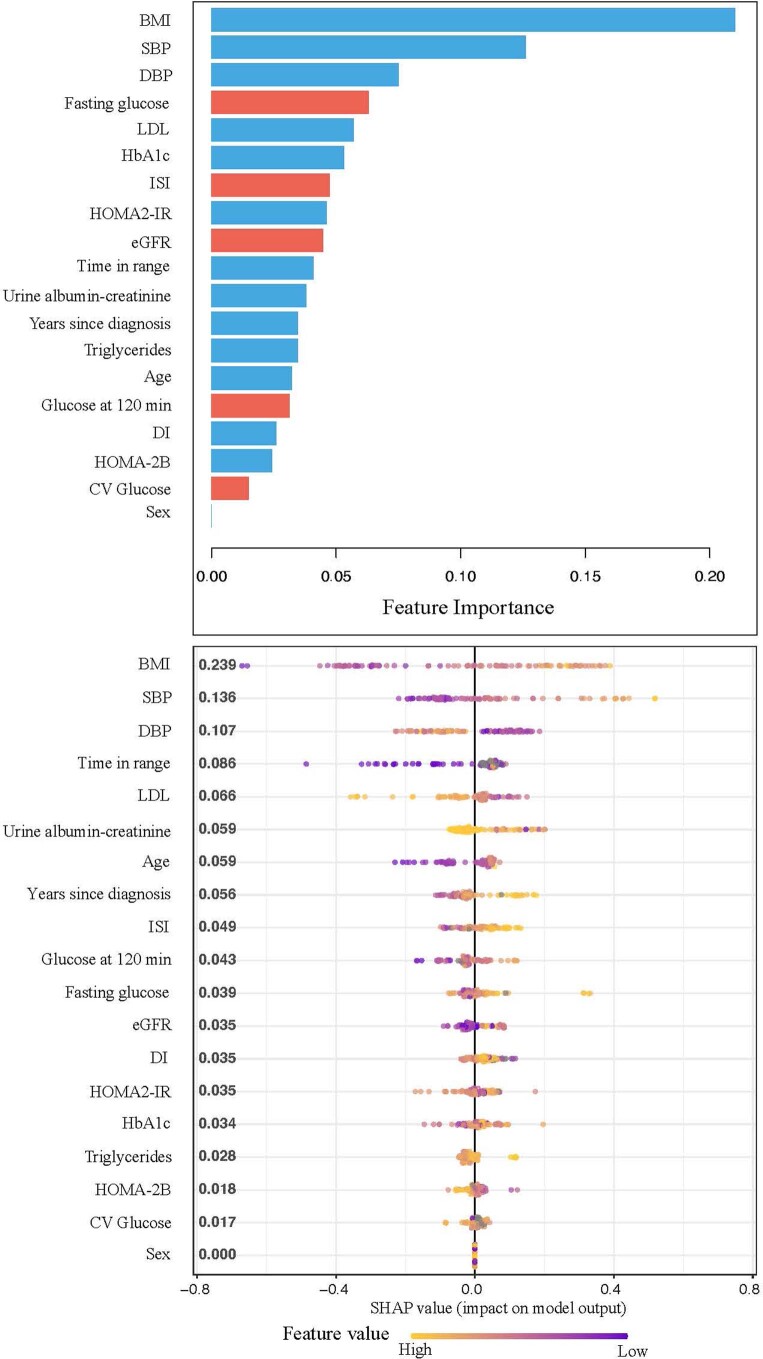
Variables predicting the change in BMI in response to semaglutide. The upper panel shows the relative importance of baseline variables (features) in predicting the change in BMI after treatment with semaglutide (n = 107). The measures of relative feature sum up to 1. All participants receiving semaglutide were included in these analyses, independent of SIDD or SIRD cluster, to investigate the continuous baseline variables across all individuals. Blue bars indicate greater reduction in BMI with higher values of the baseline trait; red indicates greater reduction in BMI with lower values of the baseline trait. Blue indication for the sex term implies greater reduction in women; red indicates greater reduction in men. The lower panel shows the SHAP summary plot for the impact of baseline variables in predicting the change in BMI in response to semaglutide. The upper panel is arranged according to the relative importance of the variable predicting the outcome across all individuals, that is denotes how important a variable is on average in predicting the treatment response across all participants. The lower panel is arranged based on the mean of the absolute SHAP values for each variable, where individual values with large impact in a specific participant can influence the mean for that particular variable, while the variable across all participants may be of less relative importance. BMI is body mass index; SBP systolic blood pressure; DBP diastolic blood pressure; eGFR estimated glomerular filtration rate; DI disposition index; ISI insulin sensitivity index; Glucose at 120 min refers to glucose measured at 120 minutes. Time in range (the fraction of time with glucose between 3.9 and 10.0 mmol/l) and CV glucose (coefficient of variance of glucose) were obtained from continuous glucose monitoring during two weeks prior to treatment allocation. Source data

**Extended Data Fig. 6:**
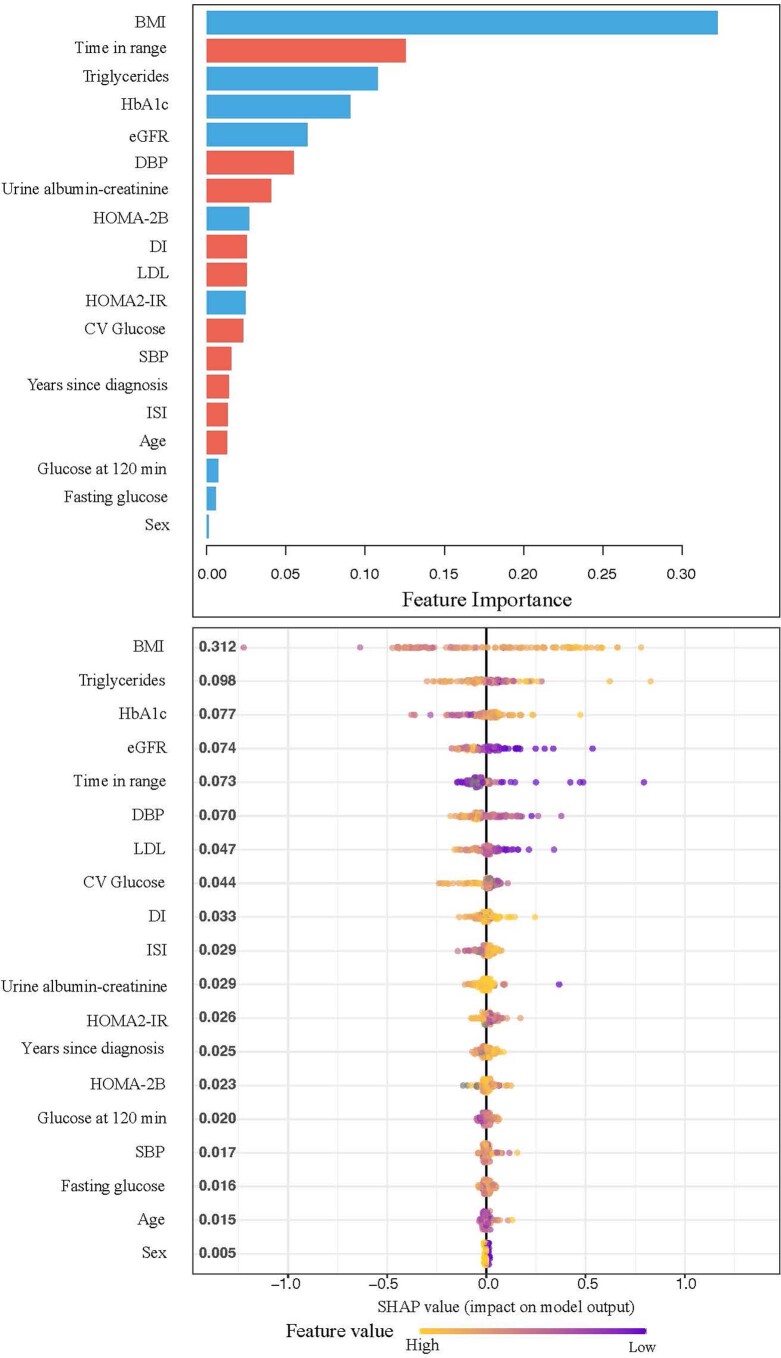
Variables predicting the change in BMI in response to dapagliflozin. The panels are arranged and bars indicated in blue or red as described for Extended Data Fig. 5 (n = 113). Source data

**Extended Data Fig. 7:**
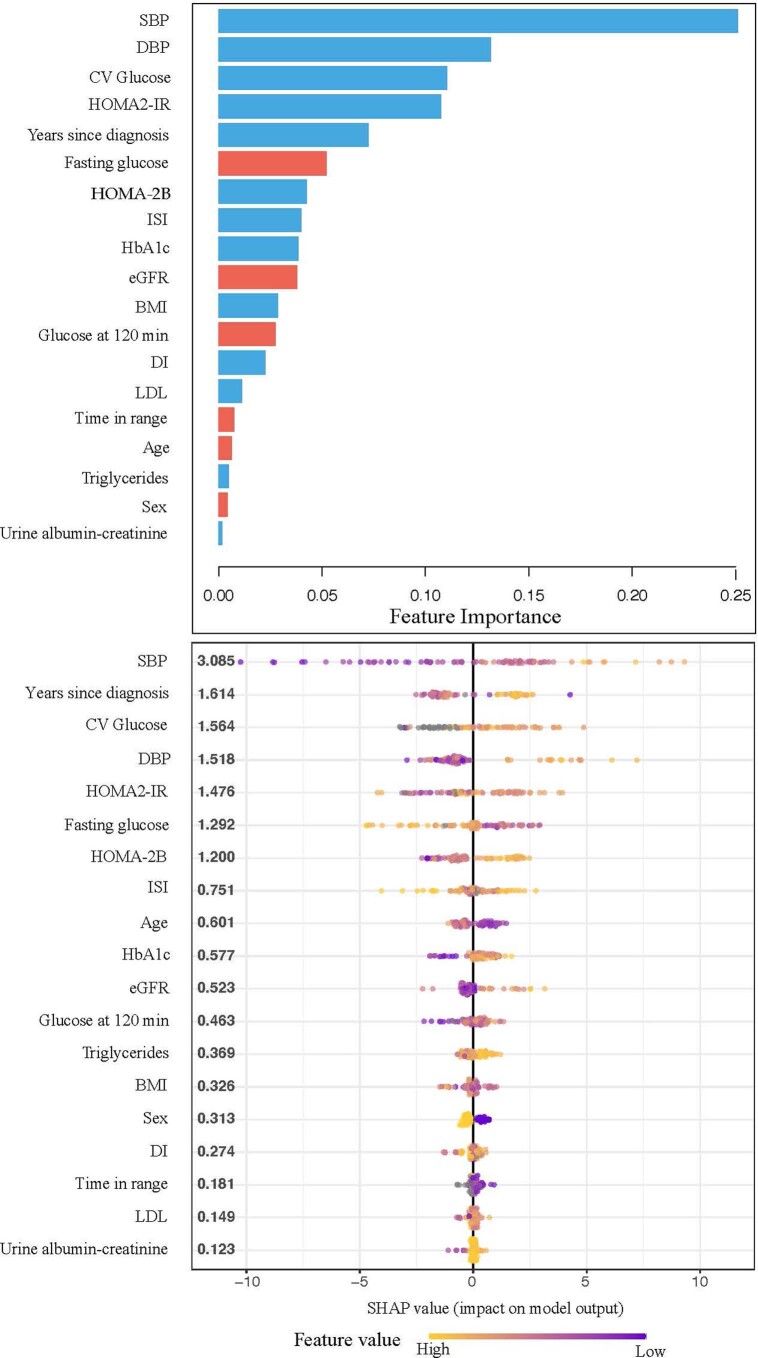
Variables predicting the change in systolic blood pressure in response to semaglutide. The panels are arranged and bars indicated in blue or red as described for Extended Data Fig. 5 (n = 107). Source data

**Extended Data Fig. 8:**
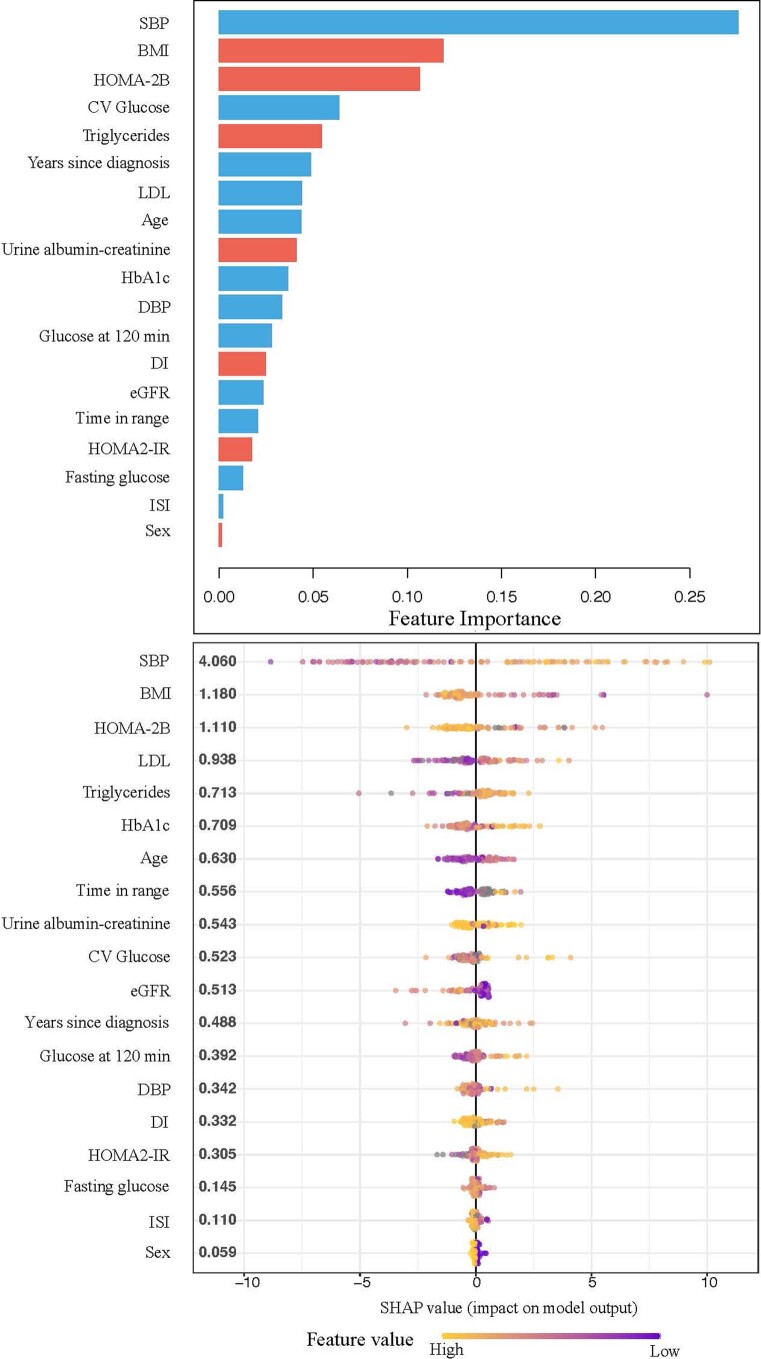
Variables predicting the change in systolic blood pressure in response to dapagliflozin. The panels are arranged and bars indicated in blue or red as described for Extended Data Fig. 5 (n = 113). Source data

**Extended Data Fig. 9:**
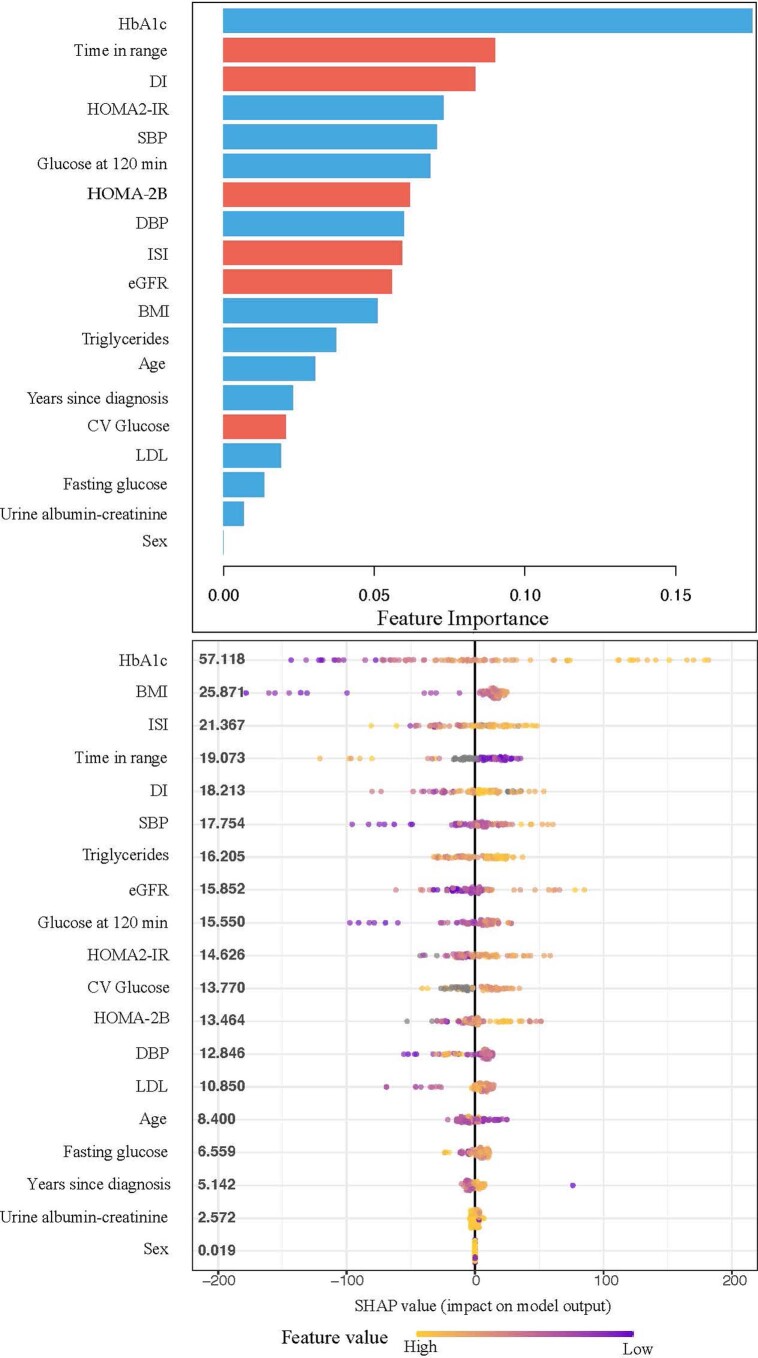
Variables predicting the combined change in HbA1c, BMI and systolic blood pressure in response to semaglutide. The panels are arranged as described for Extended Data Fig. 5 (n = 107). Source data

**Extended Data Fig. 10:**
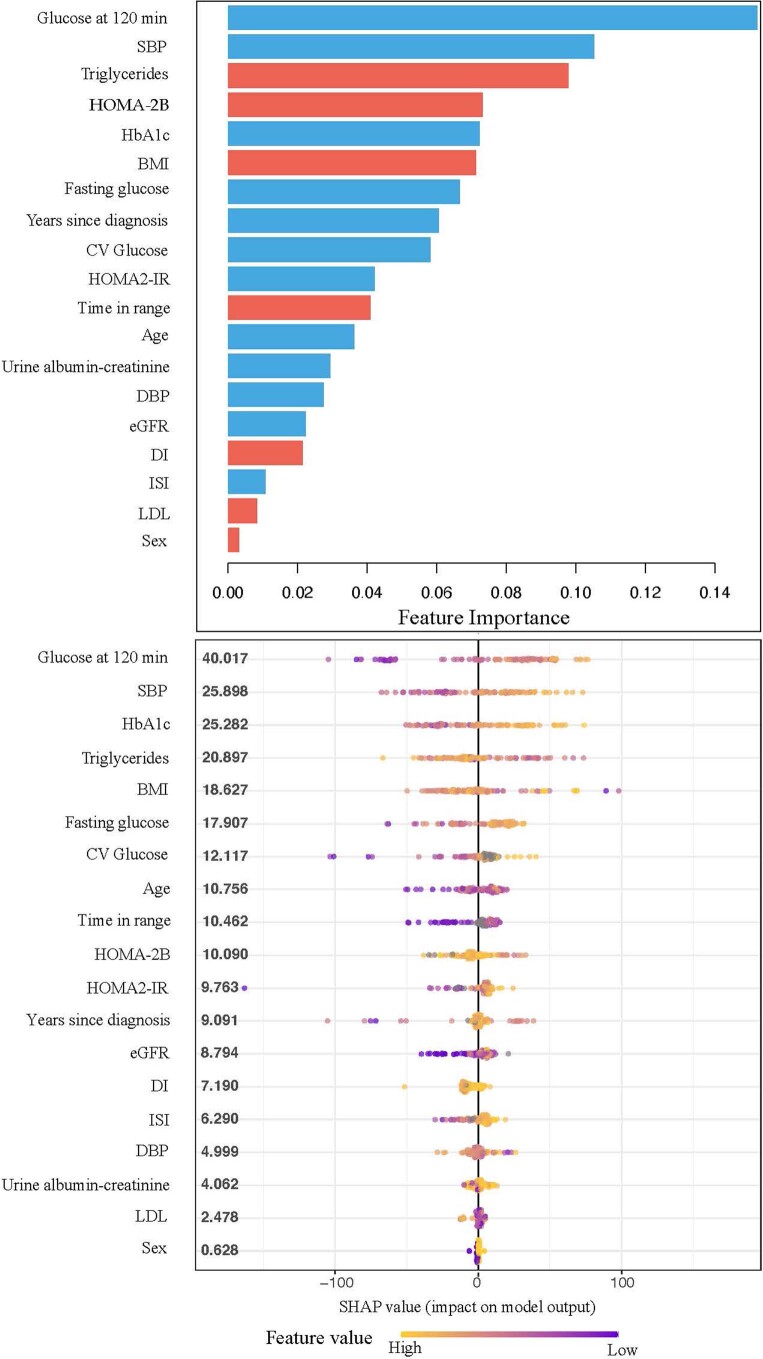
Variables predicting the combined change in HbA1c, BMI and systolic blood pressure in response to dapagliflozin. The panels are arranged as described for Extended Data Fig. 5 (n = 113). Source data

Several factors, including comorbidities, could make one drug preferred over the other. Even in those cases, it is clinically important, considering side effects and costs, to estimate the likely metabolic response before medication is instigated (for example, ~30% of participants receiving dapagliflozin had no glycaemic improvement). Therefore, we analysed the association between baseline traits and treatment response using linear regression (Supplementary Table 11). In addition, we used machine learning based on decision trees to evaluate the combined influence of baseline variables on the variation in treatment response, also considering nonlinear effects. This is a supportive analysis that should be validated in additional cohorts. We focused on the outcomes of HbA1c, BMI and systolic blood pressure, which are important risk factors for cardiovascular and renal diseases^1^. This analysis included the full cohort, independent of the subgroup, and showed that the strongest predictors of the change in HbA1c in response to semaglutide were baseline HbA1c, fasting glucose, low-density lipoprotein (LDL) cholesterol, time in range and HOMA2-B (Extended Data Figs. 3 and 4). In contrast, the change in HbA1c in response to dapagliflozin was mainly predicted by HbA1c, BMI and systolic blood pressure (the latter probably due to altered sympathetic activity in response to the SLGT2i^20–22^). The main predictors of the change in BMI and systolic blood pressure are shown in Extended Data Figs. 5–8. Taken together, these observations indicate that the study participants with the largest improvements in HbA1c, BMI and systolic blood pressure in response to semaglutide were those with high HbA1c, fasting glucose and blood pressure and low estimates of insulin secretion at baseline. In parallel, those with high baseline HbA1c, blood pressure, BMI and estimated glomerular filtration rate (eGFR) had the greatest response to dapagliflozin. This was further supported using a composite measure for the change in HbA1c, BMI and systolic blood pressure (Extended Data Figs. 9 and 10).

The trial represents a systematic test of stratified treatment in subgroups using a GLP1ra and an SGLT2i, and compares stratification with an alternative approach based on continuous variables. The results showed no interaction between the subgroup and the effect of semaglutide and dapagliflozin on glycaemic improvement. In contrast, continuous pathophysiological variables can predict the likely treatment response to these common drugs and provide more information than stratified subgroups.

The overall reductions in HbA1c in response to the study compounds are similar to those reported in phase 2 or 3 trials including patients with corresponding baseline HbA1c^23–26^. In the present study, semaglutide treatment resulted in a larger glycaemic improvement in participants with SIDD than in those with SIRD. Although there has been no precedent to this observation, post hoc analyses of earlier trials with semaglutide have shown a larger glycaemic effect in patients with high baseline HbA1c^24^, whereas the influence of baseline BMI remains unclear^23,27^. In parallel, post hoc analyses of studies of SGLT2i drugs have reported greater glycaemic efficacy in patients with high baseline HbA1c and eGFR, which is expected from the mechanism of action; however, no consistent influence of sex or age (when adjusting for eGFR) has been shown^28^. In contrast to these post hoc analyses, the present pathophysiological assessments are more comprehensive and may also be more representative of the real clinical scenario, as they are data-driven and consider the combined effects of baseline variables, including data on insulin secretion and insulin resistance, instead of manual cut-offs of baseline traits as in previously published trials. In addition to the influence of baseline HbA1c on the glycaemic efficacy of the compounds, the results highlight clinical and pathophysiological traits that add new information and could further improve the overall assessment of which patients will benefit most from these common drugs.

Although semaglutide induced a greater average improvement in HbA1c than dapagliflozin across all participants, the effect of semaglutide was particularly pronounced compared with dapagliflozin in individuals with high HbA1c, elevated fasting and postprandial glucose, low time in range and poor insulin secretion. In parallel, the response to dapagliflozin was relatively more pronounced compared with semaglutide (albeit lower in absolute terms) in those with low BMI, high time in range and high insulin secretion.

Considering side effects and costs, it is often essential to estimate the likely metabolic response before medication is initiated. In this context, it is notable that patients with the largest improvements in HbA1c, BMI and systolic blood pressure in response to semaglutide were characterized by high HbA1c, fasting glucose and blood pressure and low insulin secretion at baseline. In parallel, patients with high baseline HbA1c, BMI, eGFR and blood pressure were most likely to respond favourably to dapagliflozin. Therefore, assessment of these variables in conjunction with cardiovascular risk factors, renal status and patient preferences may be used collectively to provide a continuous and patient-specific model for personalized treatment.

International guidelines emphasize the need to instigate cotreatment in addition to metformin at an early stage in patients with an increased risk of treatment failure and in those who are likely to experience large reductions in cardiovascular risk factors (for example, HbA1c, BMI and blood pressure)^1^. The study results point to the usefulness of C-peptide determination, which is not consistently part of clinical routine, to support such evaluations in the individual case. The availability of C-peptide data allows for estimating beta-cell function (HOMA2-B), which was shown to convey information on the likely treatment response. Deteriorating beta-cell function is the main determinant of disease progression^1,29,30^, and the present data emphasize the need to identify and treat patients with poor beta-cell function more intensely.

The study’s strength is that it tests the concept of personalized treatment for diabetes in a randomized trial specifically designed to investigate the metabolic response in patients with different pathophysiology. The finding that subgroups do not effectively inform on the glycaemic response to GLP1ra or SGLT2i drugs is of considerable clinical and conceptual importance, particularly because the data highlight a more effective approach based on continuous variables. The trial investigates both GLP1ra and SGLT2i drugs in the same randomized setting^1^. International guidelines recommend GLP1ra or SGLT2i treatment in patients with established cardiovascular disease, heart failure or chronic kidney disease, but this encompasses only ~20% of patients with type 2 diabetes^1^. Thus, it is generally unclear which patients will benefit most from these drugs. The trial results add further knowledge on the overall effects of GLP1ra and SGLT2i drugs, in addition to considerations based on cardiorenal comorbidities. This is essential given the high costs of these drugs.

The study also has some limitations. Several other drug combinations are possible. The rationale for choosing a GLP1ra and an SGLTi includes their cardiovascular benefits, the clinical need for improved knowledge of their efficacy in patients with different disease characteristics and their increased usage. We focused on the SIDD and SIRD subgroups because they represent patients with different pathophysiological features and an increased risk of complications. However, this is also a limitation, as the study participants do not represent all identified subgroups. The requirement for metformin monotherapy at inclusion (to allow for strict comparisons of semaglutide versus dapagliflozin) excludes patients with more severe disease progression. Extensive evidence has shown that improved control of HbA1c, blood pressure and BMI reduces macrovascular and microvascular events^1,31^. However, the study duration does not allow for specific analysis of long-term complications, which should be the focus of future studies.

In summary, we found that semaglutide induces a larger reduction in HbA1c than dapagliflozin, but there is no differential response between the two subgroups. In contrast, continuous pathophysiological variables or tertiles of HbA1c provide more information on the treatment response to GLP1ra and SGLT2i drugs than stratified subgroups. The findings show that systematic evaluation of glycaemic control, BMI, systolic blood pressure, insulin secretion and insulin resistance can be used to identify patients who are likely to receive the greatest metabolic benefit from semaglutide or dapagliflozin, and can provide a fuller picture of the overall effects than what is currently available. Thus, the results could help guide further clinical and scientific efforts in precision medicine and allow for more informed use of antidiabetic drugs.

## Methods

### Trial design and oversight

We conducted an investigator-initiated, randomized, parallel-arm trial at Clinical Studies Sweden, Forum South, Lund University Hospital, Lund, Sweden. The trial started on 10 August 2020 and was conducted in accordance with the principles of the Declaration of Helsinki and Good Clinical Practice. The protocol was approved by the Swedish Ethical Review Authority (2020-01353) and the Medical Products Agency (EudraCT 2020-000109-33). The study was conducted by academic investigators, and funders had no role in data interpretation. The trial was monitored by an independent monitor group before, during and after its completion to ensure that it was carried out according to the protocol. All authors had access to the data, were involved in the writing and editing of the manuscript, vouched for the completeness and accuracy of the data, and agreed to submit the manuscript for publication. The trial is registered at ClinicalTrials.gov (NCT04451837).

### Participants

Study participants were recruited from the All New Diabetics in Scania (ANDIS) cohort^3^. ANDIS aims to register all incident cases of diabetes in Scania, one of the largest regions in Sweden, with 1,200,000 inhabitants in both rural and urban areas and a wide distribution of socioeconomic backgrounds. Approximately 27,000 persons with diabetes (>90% of the estimated number of eligible cases in the region) have been included from 2008 to 2022. Most patients with type 2 diabetes are managed in primary care.

A data-driven cluster analysis has been performed in ANDIS based on six variables measured at diagnosis: HbA1c, GAD antibodies, age, BMI, HOMA2-IR and HOMA2-B (the last two variables were derived from fasting glucose and C-peptide measurements)^3^. This highlighted five clusters of patients with diabetes, each with different pathophysiological characteristics^3,16,17^. Cluster assignment of any patient can be done clinically with an algorithmic tool that uses age at diagnosis, GAD antibodies, BMI, HbA1c, fasting glucose and C-peptide as input variables, and determines the shortest distance between the individual values and the five different cluster centroids based on the distribution of these variables in the full ANDIS cohort^3^.

Patients with diabetes mellitus were invited through letters or advertisements. Those who belonged to the SIDD or SIRD group according to the clustering algorithm, had received metformin monotherapy at a constant dose for at least 3 months, and had HbA1c levels of ≥42 and <91 mmol mol^−1^ were eligible for enrolment. A complete list of the inclusion and exclusion criteria is provided below. All participants provided written informed consent before study entry. Sex was determined based on self-report and the official social security number. Participants received travel reimbursement but no other financial compensation.

### Inclusion criteria

Participants were eligible to be included in the trial if all the following criteria applied:

- Diabetes mellitus based on prior documentation or treatment with an antihyperglycaemic medication or diagnosed according to the World Health Organization criteria (random plasma glucose >11.1 mmol l^−1^ or fasting glucose >7.0 mmol l^−1^ or HbA1c ≥ 6.5%), and disease characteristics typical of SIDD or SIRD according to the ANDIS clustering.
- Ongoing metformin therapy at a constant dose in the last 90 days.
- Age ≥18 years.
- HbA1c ≥42 and <91 mmol mol^−1^.
- Women who were not postmenopausal and who had not undergone surgical sterilization must have no current pregnancy, assessed by a pregnancy test; must take precautions to avoid pregnancy throughout the study and for 4 weeks after the last dose; and must be willing to use highly effective birth control methods.
- Willingness to receive injectable and oral medications.
- Written informed consent.

### Exclusion criteria

Participants were excluded from the trial if any of the following applied:

- Type 1 diabetes, latent autoimmune diabetes in adults, maturity-onset diabetes of the young, secondary diabetes or history of diabetic ketoacidosis.
- Antidiabetic treatment other than metformin within 90 days before inclusion.
- A known acute cardiovascular event within 90 days before inclusion.
- Heart failure of New York Heart Association class IV.
- History of acute or chronic pancreatitis.
- Liver cirrhosis.
- Blood pressure >170/110 mm Hg.
- Current chronic daily treatment with an orally administered steroid at a dose equivalent to ≥10 mg of orally administered prednisolone.
- Pregnancy or breastfeeding.
- Known galactose intolerance, total lactase deficiency or glucose–galactose malabsorption.
- Inability to understand the study information.
- Involvement in the planning and/or conduct of the study.
- Participation in other clinical trials, which may affect the outcome of the present study.
- Any condition or treatment that, in the judgement of the investigator, makes it difficult or unsafe to participate in the study.
- eGFR < 45 ml min^−1^ 1.73 m^−2^ or unstable or rapidly progressing renal disease.
- An aspartate aminotransferase (ASAT) or alanine aminotransferase (ALAT), alkaline phosphatase (ALP) or bilirubin level of more than three times the upper limit of the normal range.

### Trial procedures

The metformin dose at inclusion (as prescribed by the primary care physician) was maintained throughout the study. After a screening visit, participants wore a Libre Pro sensor (Abbott Diabetes Care) for continuous glucose monitoring for 2 weeks. This was followed by a first study visit for baseline measurements, including an OGTT with venous blood sampling at 0, 30, 60, 90 and 120 min after ingesting 75 g of glucose. At the end of this visit, the participants were randomized to receive either semaglutide (Ozempic, Novo Nordisk) or dapagliflozin (Forxiga, AstraZeneca) in addition to metformin (Extended Data Fig. 1). Three months after randomization, the participants attended a visit for measurement of HbA1c and safety variables and application of another glucose sensor to wear for 2 weeks. Six months after randomization, they attended a final study visit for measurement of primary and secondary variables, including an OGTT. Participants who discontinued before the scheduled 6-month visit were invited to attend an immediate final visit before suspending their study medication.

Semaglutide was injected at a dose of 0.25 mg subcutaneously once weekly during the first 4 weeks, followed by 0.5 mg weekly for the subsequent 4 weeks and, finally, 1.0 mg weekly throughout the trial (the maximal dose approved for diabetes treatment). A dose reduction to 0.5 mg was allowed if the participant experienced unacceptable side effects at 1.0 mg. Those randomized to dapagliflozin treatment received 10 mg orally once daily (the maximal dose approved for diabetes treatment). Participants on semaglutide were instructed to adjust the administration schedule such that the last dose was taken 3 days before their final visit to standardize administration in relation to the final OGTT. In parallel, participants on dapagliflozin were instructed to take their tablet in the evening at least 3 days before their final visit, including the evening before the visit. These considerations were based on the pharmacokinetics and routes of administration of the study drugs to provide an average steady-state condition for both drugs before the OGTT.

The randomization was generated by independent statisticians using a computer-based block randomization algorithm with balanced blocks. Randomization was stratified for SIDD and SIRD to obtain an approximate distribution of semaglutide and dapagliflozin in a 1:1 ratio in both subgroups. Allocation was concealed (through sealed envelopes) to participants and study personnel until the end of the first visit, after measurements of baseline variables had been completed. Thus, random sequence generation, participant enrolment by study personnel and allocation to randomization groups were clearly separated. After randomization, the assignment became open label, and data collection and analysis were not performed blind to the conditions of the experiments.

### Outcomes

The primary variable was the change from baseline in HbA1c. The secondary variables were the change from baseline in BMI, waist circumference, urinary albumin/creatinine index, blood pressure, LDL cholesterol, triglycerides, HOMA2-B, HOMA2-IR, disposition index, insulin sensitivity index and glucose at 0 and 120 min measured from the OGTT in participants with SIDD versus those with SIRD using intraindividual comparisons. By continuous glucose monitoring for 2 weeks both before and at 3 months of the intervention, the change in glucose variability (estimated as the coefficient of variance of glucose concentration), average glucose concentration and time in range (glucose between 3.9 and 10 mmol l^−1^) from baseline were also analysed. Patient-reported outcomes were analysed at baseline and after treatment. In addition, plasma sodium, potassium, albumin, and creatinine and cystatin C (both used to calculate eGFR) were analysed as safety variables. Primary, secondary and safety endpoints were reported from the full analysis set.

### Study analyses

Venous blood samples were taken in the morning (between 7.30 and 10.00). Participants were instructed to fast from 10 p.m. of the previous day. They were also instructed to avoid nicotine use on the same day, as well as alcohol consumption and strenuous physical activity within 24 h of the visit. Fasting blood glucose was measured at the study centre using a HemoCue glucose system (HemoCue). All other blood analyses were performed at the central hospital laboratory (Lund, Sweden). HbA1c was analysed according to the International Federation of Clinical Chemistry standard using a Capillarys 3 TERA HbA1c kit. C-peptide, insulin, LDL cholesterol and triglycerides were measured on a Cobas analyser (Roche Diagnostics). Urinary albumin/creatinine index was measured using the Atellica CH Microalbumin 2 (μALB_2) assay (Siemens Healthcare Diagnostics). Creatinine and cystatin C, plasma sodium, potassium and albumin were measured on an Atellica analyser (Siemens Healthcare Diagnostics).

HOMA2-IR and HOMA2-B were calculated based on the C-peptide concentration (which performs better than insulin concentration in individuals with type 2 diabetes) using the HOMA calculator (University of Oxford, Oxford, UK)^32^.

Blood glucose and venous plasma insulin were analysed at 0, 30, 60, 90 and 120 min of the OGTT. The insulin sensitivity index was calculated as 10,000 divided by the square root of fasting glucose times fasting insulin times the average glucose times the average insulin concentration during the OGTT. The disposition index, which reflects insulin secretion adjusted for insulin resistance, was determined as the product of the insulin sensitivity index and the amount of insulin secreted relative to the glucose concentration during the OGTT.

The NAFLD liver fat score was calculated as previously described, based on the presence of type 2 diabetes and metabolic syndrome, fasting insulin concentration, ASAT level and the ASAT/ALAT ratio^33^. We used a cut-off of >−1.413, which has been shown to predict NAFLD with 95% accuracy^33^.

Blood pressure was measured using a standardized cuff adapted to the size of the participant’s arm after the participant had rested in a sitting position for at least 5 min. Height was recorded in centimetres and body weight in kilograms, to one decimal place, while the participant was wearing light clothing and no shoes. Waist circumference was measured in centimetres as the minimal abdominal circumference located midway between the lower rib margin and the iliac crest.

The participants brought samples of first-morning urine to the first and last visits. A 10-ml aliquot was sent to the hospital laboratory for analysis of the albumin/creatinine index. Stool sampling before each study visit was optional; those samples will be used for exploratory analyses in subsequent projects.

A Freestyle Libre Pro sensor was used to monitor glucose continuously in study participants. The study personnel placed a pad on the participants’ arm. The participants wore the pad for 2 weeks at baseline and when they had been on the study medication for 3 months. The sensor had no connected reader, which means that the participants did not receive any feedback on their glucose values.

Glucose variability (measured as the coefficient of variance of glucose concentration), time in range (glucose between 3.9 and 10 mmol l^−1^) and average glucose concentrations were analysed.

### Control of Eating Questionnaire

We used the Control of Eating Questionnaire with 19 items assessing craving control, positive mood, craving for savoury foods and craving for sweet foods^34^. This is relevant because both dapagliflozin and semaglutide may affect appetite^35^. Participants were asked to respond according to their experience over the previous 7 days at baseline and their final visit after treatment. Each item was evaluated using 10-cm visual analogue scales. The scores of each item were averaged across the study participants and are reported in Supplementary Table 5. The individual items, scored from left (0, ‘not at all’) to right (10, ‘extremely’), were as follows:

1. How hungry have you felt?
2. How full have you felt?
3. How strong was your desire to eat sweet foods? How strong was your desire for nonsweet tasty foods (French fries, potato chips, hamburgers, pizza)?
4. How happy have you felt?
5. How anxious have you felt?
6. How alert have you felt?
7. How contented have you felt?
8. During the last 7 days, how often have you had food cravings (not at all/very often)?
9. How strong have any food cravings been?
10. How difficult has it been to resist any food cravings?
11. How often have you eaten in response to food cravings (not at all/after every one)?

How often have you had cravings for the following (graded from not at all to extremely often):

1. Chocolate or chocolate-flavoured foods?
2. Other sweet foods (cakes, pastries, chocolate, etc.)?
3. Fruit or fruit juice? Dairy foods (cheese, yogurt, milk, etc.)?
4. Starchy foods (bread, rice, pasta, etc.)?
5. Tasty foods that are not sweet (French fries, potato chips, burgers, pizza, etc.)?
6. Generally, how difficult has it been to control your eating (not at all difficult/extremely difficult)?

### Diabetes Treatment Satisfaction Questionnaire

The Diabetes Treatment Satisfaction Questionnaire, which measures treatment satisfaction with six items, was completed by participants at baseline and at the final visit after treatment^36^. The responses to the items, on a Likert scale ranging from 0 to 6, were summed and used for analysis. The total score ranges from 0 to 36, with larger values indicating higher satisfaction with treatment. In addition, it has one item assessing subjective experiences of unacceptably high blood glucose and one item assessing the experience of unacceptably low blood glucose. These scales range from 0 to 6, with higher scores indicating a higher frequency of unacceptably high or low blood glucose.

### Identification of continuous baseline variables predicting drug response

We used XGBoost (extreme gradient boosting), an ensemble machine learning technique based on several sequential decision trees, to identify continuous baseline variables predicting the change in HbA1c, BMI and systolic blood pressure after treatment with the study drugs. The method develops a multivariable ensemble of prediction models used to identify the strongest predictors. The optimal values for hyperparameters for each outcome were detected by performing a grid search on several possible combinations of different variables. The hyperparameters included the number of trees, learning rate, minimal loss to expand on a leaf node, maximum tree depth and subsample proportion. All the other parameters were used at their default values. The package XGBoost version 1.6.0.1 was used in R 4.1.0.

We computed the relative importance of each variable predicting the outcome using *F* scores in XGBoost, calculated as the sum of Gini improvement among the corresponding splits within a tree, averaged over all trees. In addition, we implemented SHapley Additive exPlanations (SHAP) for easy interpretation of the machine learning model output. The SHAP value in our study was the mean of the absolute individual feature-level impact on the model. The training set in our models consisted of a randomly selected subset of 80% of the study participants, and the testing set was composed of the remaining 20%. The model was based on data from the training set; the testing set was independent of the training process and was used only for performance evaluation after the model was established. The composite outcome measure of the change in HbA1c, BMI and systolic blood pressure was calculated by multiplying the normalized values of each outcome variable.

### Statistical analysis

The primary endpoint was the intraindividual change in HbA1c from baseline in response to semaglutide or dapagliflozin in participants with SIDD versus those with SIRD. It was analysed using an analysis of covariance model. The model included the metformin dose and HbA1c at baseline and an interaction term for the study drug (semaglutide or dapagliflozin) and subgroup (SIDD or SIRD). In similar models, we also evaluated the interaction between the variables used for cluster designation (HbA1c, BMI, age, sex, HOMA2-B and HOMA2-IR) and the study drug. The data met the assumptions of the statistical tests used.

Analyses using *z* scores normalized the individual change in HbA1c based on means and s.d. values within each treatment group (semaglutide or dapagliflozin), and were used in comparisons of the relative efficacy of the drugs after adjustment for baseline HbA1c.

Secondary endpoints included the change in secondary variables; intraindividual delta values were obtained by comparing secondary variables between the baseline and last visits for each participant. The intraindividual delta values were then analysed across all participants using independent *t* tests.

The full analysis set included all participants who had at least one HbA1c measurement after randomization, independent of compliance, duration of participation or potential dose reduction of semaglutide from 1.0 to 0.5 mg. Missing data were not imputed.

The study was designed to have 80% power to detect a treatment effect between the clusters, assuming that the true difference in treatment effect between clusters was 3 mmol mol^−1^ (which would be clinically relevant given previous reports on the association between HbA1c and vascular complications^31^). This applied to both dapagliflozin and semaglutide. The s.d. of the change in HbA1c over 6 months was 4.9 mmol mol^−1^ (as observed in the ANDIS cohort). At an alpha of 0.05, at least 86 participants with SIDD and 86 participants with SIRD were required, and we planned to recruit a total of 100 participants with SIDD and 100 participants with SIRD. This also means 80% power to detect a treatment-by-subgroup interaction of 3.5 mmol mol^−1^.

Two-sided *P* values of ≤0.05 were considered to indicate statistical significance. Summary statistics are generally presented as point estimates with 95% CIs. The *P* values from the hypothesis tests and the widths of the intervals have not been adjusted for multiplicity. Statistical analyses were performed using SPSS (version 26, IBM) or R 4.1.0.

### Reporting summary

Further information on research design is available in the Nature Portfolio Reporting Summary linked to this article.

### Supplementary information

Supplementary Information Supplementary Tables 1–11.

Reporting Summary

Supplementary Data 1 Study protocol and statistical analysis plan.

Supplementary Data 2 CONSORT checklist.

### Source data

Source Data Fig. 1 Statistical source data for Fig. 1.

Source Data Fig. 2 Statistical source data for Fig. 2.

Source Data Extended Data Fig. 1 Statistical source data for Extended Data Fig. 1 with study overview.

Source Data Extended Data Fig. 2 Statistical source data for Extended Data Fig. 2 with changes over time in eGFR and liver parameters.

Source Data Extended Data Fig. 3 Statistical source data for Extended Data Fig. 3 with continuous baseline variables predicting the change in HbA1c.

Source Data Extended Data Fig. 4 Statistical source data for Extended Data Fig. 4 with baseline variables predicting the change in HbA1c in response to dapagliflozin or semaglutide.

Source Data Extended Data Fig. 5 Statistical source data for Extended Data Fig. 5 with variables predicting the change in BMI in response to semaglutide.

Source Data Extended Data Fig. 6 Statistical source data for Extended Data Fig. 6 with variables predicting the change in BMI in response to dapagliflozin.

Source Data Extended Data Fig. 7 Statistical source data for Extended Data Fig. 7 with variables predicting the change in systolic blood pressure in response to semaglutide.

Source Data Extended Data Fig. 8 Statistical source data for Extended Data Fig. 8 with variables predicting the change in systolic blood pressure in response to dapagliflozin.

Source Data Extended Data Fig. 9 Statistical source data for Extended Data Fig. 9 with variables predicting the combined change in HbA1c, BMI and systolic blood pressure in response to semaglutide.

Source Data Extended Data Fig. 10 Statistical source data for Extended Data Fig. 10 with variables predicting the combined change in HbA1c, BMI and systolic blood pressure in response to dapagliflozin.

## Data Availability

Data requests should be submitted to the corresponding author (anders.rosengren@gu.se). Access to anonymized data will be granted following review (time frame <20 office days) to ensure compliance with relevant ethical and legal considerations. The study protocol is appended with the paper and available online. Source data are provided with this paper.
