# Supplementary material for: Randomized open-label trial of semaglutide and dapagliflozin in patients with type 2 diabetes of different pathophysiology
Source: Nat Metab. 2024 Jan 4;6(1):50–60. doi: 10.1038/s42255-023-00943-3 (PMC10822775; doi:10.1038/s42255-023-00943-3)
Supplement: Supplementary file 1 — Supplementary Tables 1–11. [file 42255_2023_943_MOESM1_ESM.pdf]

nature metabolism

Letter

https://doi.org/10.1038/s42255-023-00943-3

Randomized open-label trial of semaglutide and dapagliflozin in patients with type 2 diabetes of different pathophysiology

In the format provided by the authors and unedited

SUPPLEMENTARY TABLES

Supplementary Table 1. Adverse events in participants who discontinued without any outcome data*

**Drug**	**SIDD**	**SIRD**	**Total**
**Semaglutide**	7	6	13
**Gastrointestinal symptoms**	4	6	10
**Urinary symptoms**	0	0	0
**Other events****	3	0	3
**Dapagliflozin**	1	5	6
**Gastrointestinal symptoms**	0	1	1
**Urinary symptoms**	0	1	1
**Other events****	1	3	4

*Participants who discontinued without any measurement of the primary outcome variable after baseline (n=19; 7 women, 12 men) were not included in the full analysis set. Of those, 13 participants reported adverse events as summarized in the table.

**Other reported events were sleep disorder, fatigue, loss of appetite and balance problems.

Supplementary Table 2. Adverse events in all participants who discontinued at any point during the study*			
**Drug**	**SIDD**	**SIRD**	**Total**
**Semaglutide**	18 (5 f, 13 m)	13 (8 f, 5 m)	31 (13 f, 18 m)
**Gastrointestinal symptoms**	14	11	25
**Urinary symptoms**	0	0	0
**Other reasons****	4	2	6
**Dapagliflozin**	6 (2 f, 4 m)	8 (2 f, 6 m)	14 (4 f, 10 m)
**Gastrointestinal symptoms**	1	4	5
**Urinary symptoms**	0	1	1
**Other reasons****	5	3	8

*A total of 50 participants (18 women, 32 men) discontinued at any point during the study. Of those, 45 (17 women, 28 men) reported adverse events as summarized in the table. Number of female (f), and male (m) participants in parentheses.

**Other reported events were sleep disorder, fatigue, loss of appetite and balance problems.

Supplementary Table 3. Any reported adverse events in study participants.*						
	Semaglutide			Dapagliflozin		
	SIDD (n=63)	SIRD (n=57)	Total (n=120)	SIDD (n=63)	SIRD (n=56)	Total (n=119)
**Nausea**	23	22	45	2	4	6
**Diarrhea**	8	13	21		3	3
**Obstipation**	4	3	7	2	2	4
**Vomiting**	9	10	19	1	1	2
**Abdominal pain**	3	3	6	2	2	4
**Urinary infection**	0	1	1	1	7	8
**Genital infection**	0	0	0	2	0	2
**Increased urination**	0	0	0	7	4	11
**Genital symptoms without infection**	0	0	0	6	8	14
**Other infection**	1	1	2	6	1	7
**Sleep disorder**	1	0	1	1	0	1
**Kidney failure**	2	1	3	1	0	1
**Covid-19**	1	0	1	1	2	3
**Dermatological symptoms**	1	0	1	0	2	2
**Eye symptoms**	2	0	2	0	0	0
**Skeletal pain**	2	2	4	7	5	12
**Vertigo****	6	6	12	2	3	5
**Hypertension**	0	0	0	1	0	1
**Fatigue**	5	4	9	3	2	5
**Anemia**	2	1	3	0	0	0
**Hypoglycaemia**	0	1	1	0	0	0
**Chest pain requiring hospitalization****	1	0	1	0	1	1
**Hip luxation****	0	1	1	0	0	0
**Gastrointestinal disorders*****	34 (12 f, 22 m)	32 (17 f, 15 m)	66 (29 f, 37 m)	6 (3 f, 3m)	9 (3 f, 6 m)	15 (6 f, 9 m)
**Urinary disorders******	0	1 (1 f)	1 (1 f)	14 (3f, 11 m)	14 (6 f, 8 m)	28 (9 f, 19 m)

*Data from all 239 enrolled participants (74 women, 165 men).

** Four participants had serious adverse events with vertigo (one female participant with SIRD on semaglutide), chest pain (two male participants), or hip luxation requiring hospitalization (one male participant).

***Total number of participants reporting nausea, diarrhoea, obstipation, vomiting or abdominal pain (number of female, f, and male, m, participants in parentheses).

****Total number of participants reporting urinary tract infection, genital infection, increased urination and genital symptoms without infection (number of female, f, and male, m, participants in parentheses).

**Supplementary Table 4. Effect of study drugs on primary and secondary endpoints.***						
	Semaglutide (n=107; 36 women, 71 men)			Dapagliflozin (n=113; 31 women, 82 men)		
**Endpoint**	**SIDD (n=56)**	**SIRD (n=51)**	**Mean difference (95% CI)****	**SIDD (n=62)**	**SIRD (n=51)**	**Mean difference (95% CI)**
**Change in HbA1c – mmol/mol**	-13.4	-10.5	-2.9 (-5.7 to -0.1)	-4.7	-2.7	-2.0 (-4.4 to 0.3)
**Change in fasting glucose – mmol/l*****	-2.7	-1.9	-0.9 (-1.6 to -0.2)	-1.6	-1.0	-0.6 (-1.3 to 0.1)
**Change in glucose at 120 min – mmol/l*****	-6.7	-5.0	-1.7 (-2.8 to -0.6)	-2.8	-1.5	-1.3 (-2.2 to -0.4)
**Change in HOMA2-B**	66.2	70.5	-4.3 (-24.6 to 16.0)	20.6	10.8	9.8 (-5.1 to 24.8)
**Change in HOMA2-IR**	0.1	0.1	0.1 (-0.3 to 0.4)	-0.2	-0.4	0.2 (-0.2 to 0.5)
**Change in disposition index**	185	166	19 (-88 to 125)	31	0	31 (-37 to 100)
**Change in insulin sensitivity index**	0.7	0.5	0.1 (-0.2 to 0.5)	0.2	-0.1	0.4 (-0.1 to 0.8)
**Change in time in range******	0.22	0.03	0.19 (0.06 to 0.33)	0.11	0.07	0.04 (-0.06 to 0.14)
**Change in average glucose – mmol/l**	-2.9	-1.8	-1.1 (-2-0 to -0.1)	-1.3	-1.0	-0.3 (-1.1 to 0.4)
**Change in coefficient of variance of glucose**	-0.02	-0.02	-0.01(-0.03 to 0.02)	0.02	0.02	0 (-0.02 to 0.02)
**Change in body mass index**	-1.9	-2.4	0.5 (-0.1 to 1.0)	-1.1	-1.3	0.2 (-0.1 to 0.4)
**Change in waist circumference – cm**	-5.8	-5.4	-0.4 (-1.9 to 1.1)	-4.1	-4.4	0.3 (-1.4 to 2.0)
**Change in systolic blood pressure – mm Hg**	-3	-6	2 (-1 to 6)	-3	0	-3 (-7 to 1)
**Change in diastolic blood pressure – mm Hg**	2	0	2 (-1 to 6)	-2	0	-2 (-5 to 1)
**Change in LDL – mmol/l**	-0.3	-0.3	0.1 (-0.3 to 0.3)	0.0	0.1	-0.1 (-0.3 to 0.0)
**Change in triglycerides – mmol/l**	-0.2	-0.3	0.1 (-0.2 to 0.4)	0.0	0.1	-0.1 (-0.3 to 0.1)
**Change in eGFR – ml/min/1.73 m^2**	-0.6	0.0	-0.6 (-3.0 to 1.8)	-3.9	-2.2	-1.7 (-3.8 to 0.5)
**Change in eGFR relative to baseline (%)**	-0.4	0.4	-0.8 (-4.5 to 3.0)	-5.1	-3.1	-2.0 (-4.9 to 0.8)
**Change in urine albumin/ creatinine index – g/mol**	-0.3	-0.4	0.1 (-0.6 to 0.9)	0.0	0.3	-0.3 (-1.2 to 0.6)
**NAFLD liver fat score**	0.2	-0.6	0.8 (0.0 to 1.6)	-0.2	-0.8	0.7 (-0.7 to 2.0)

**Change in HbA1c in per protocol set – mmol/mol*******	-14.3	-11.6	-2.8 (-6.1 to 0.6)	-5.1	-3.2	-1.9 (-4.4 to 0.6)
**Change in primary outcome variable in response to study drugs disaggregated for women and men********						
	**Semaglutide in female participants**			**Dapagliflozin in female participants**		
	**SIDD (n=16)**	**SIRD (n=20)**	**Mean difference (95% CI)**	**SIDD (n=16)**	**SIRD (n=15)**	**Mean difference (95% CI)**
**Change in HbA1c – mmol/mol**	-13.7	-11.2	-2.5 (-8.7 to 3.6)	-3.1	-2.1	-1.0 (-5.1 to 3.1)
	**Semaglutide in male participants**			**Dapagliflozin in male participants**		
	**SIDD (n=40)**	**SIRD (n=31)**	**Mean difference (95% CI)**	**SIDD (n=46)**	**SIRD (n=36)**	**Mean difference (95% CI)**
**Change in HbA1c – mmol/mol**	-13.2	-10.1	-3.2 (-6.3 to 0.0)	-5.3	-3.0	-2.4 (-5.2 to 0.5)

*Changes relative to baseline in primary and secondary endpoints in response to semaglutide and dapagliflozin, respectively, in the full analysis set of participants with SIDD or SIRD.

**Estimated mean differences of values in SIDD minus SIRD participants (two-sided, unadjusted for multiple comparisons) were analyzed by an ANCOVA model and are presented as means with 95% confidence intervals.

***The effect on fasting glucose in response to dapagliflozin was 57% to that of semaglutide, while the effect on glucose at 120 minutes of the OGTT in response to dapagliflozin was 38% to that of semaglutide. In absolute terms, the change of 120-minute glucose was on average 3.6 mmol/mol larger than the change of fasting glucose in response to semaglutide. In parallel, the change of 120-minute glucose was 0.9 mmol/mol larger than the change of fasting glucose in response to dapagliflozin (mean difference 2.7 mmol/mol between the drugs [95% CI 2.0 to 3.3]).

****Time in range (fraction of time with glucose between 3.9 and 10.0 mmol/l), average glucose and coefficient of variance of glucose obtained from continuous glucose monitoring during two weeks.

*****The change in HbA1c in participants treated according to the protocol, with full doses and complete study visits (n=178; 54 women, 124 men), instead of the full analysis set.

******Effect of study drugs in subgroups disaggregated by female and male participants.

Supplementary Table 5. Patient-reported outcomes between SIDD and SIRD groups at baseline and after treatment with the study drugs*			
	**Baseline (n=107 SIDD [30 f, 77 m] and 91 SIRD [30 f, 61 m])**	**After treatment with semaglutide (n=46 SIDD [13 f, 33 m] and 43 SIRD [16 f, 27 m])**	**After treatment with dapagliflozin (n=61 SIDD [17 f, 44 m] and 48 SIRD [14 f, 34 m])**
**COEQ1****	0.50 (-0.12 to 1.1)	0.81 (0.05 to 1.57)	-0.07 (-0.82 to 0.69)
**COEQ2**	-0.46 (-1 to 0.09)	0.52 (-0.4 to 1.44)	-0.01 (-0.74 to 0.73)
**COEQ3**	0.28 (-0.56 to 1.11)	0.96 (-0.13 to 2.05)	-0.58 (-1.59 to 0.44)
**COEQ4**	0.26 (-0.48 to 0.99)	0.30 (-0.87 to 1.45)	-0.76 (-1.65 to 0.15)
**COEQ5**	0.24 (-0.36 to 0.83)	-0.31 (-2.91 to 2.29)	-0.18 (-0.89 to 0.55)
**COEQ6**	0.39 (-0.26 to 1.03)	0.15 (-0.71 to 1)	-0.15 (-0.98 to 0.68)
**COEQ7**	0.44 (-0.15 to 1.02)	0.93 (0.06 to 1.81)	0.15 (-0.61 to 0.9)
**COEQ8**	0.37 (-0.24 to 0.98)	1.36 (0.51 to 2.21)	0.05 (-0.66 to 0.76)
**COEQ9**	0.08 (-0.57 to 0.72)	0.58 (-0.31 to 1.47)	-0.56 (-1.33 to 0.22)
**COEQ10**	0.15 (-0.5 to 0.78)	0.60 (-0.19 to 1.37)	-0.59 (-1.34 to 0.18)
**COEQ11**	-0.01 (-0.73 to 0.72)	1.22 (0.36 to 2.07)	-0.69 (-1.49 to 0.12)
**COEQ12**	0.22 (-0.56 to 1)	1.33 (-0.51 to 3.17)	0.09 (-0.9 to 1.06)
**COEQ13**	0.15 (-0.65 to 0.93)	0.48 (-0.67 to 1.61)	-0.45 (-1.42 to 0.52)
**COEQ14**	-0.21 (-0.97 to 0.57)	-0.3 (-1.68 to 1.09)	-0.33 (-1.26 to 0.61
**COEQ15**	-0.84 (-1.61 to -0.08)	-0.34 (-1.42 to 0.75)	-0.74 (-1.71 to 0.24)
**COEQ16**	-0.39 (-1.17 to 0.39)	0.31 (-0.78 to 1.39)	-0.09 (-0.99 to 0.81)
**COEQ17**	0.13 (-0.57 to 0.83)	0.98 (0.08 to 1.88)	-0.17 (-1.02 to 0.70)
**COEQ18**	0.15 (-0.55 to 0.83)	0.63 (-0.33 to 1.58)	-0.01 (-0.8 to 0.78)
**COEQ19**	-0.04 (-0.76 to 0.69)	0.31 (-0.61 to 1.21)	-0.30 (-1.1 to 0.51)
**High blood glucose*****	0.67 (0.14 to 1.21)	0.58 (-0.04 to 1.18)	0.36 (-0.19 to 0.89)
**Low blood glucose******	-0.22 (-0.53 to 0.09)	-0.16 (-0.67 to 0.35)	-0.01 (-0.36 to 0.35)

*The items from the Control of eating questionnaire (COEQ1-19) and the Diabetes treatment satisfaction questionnaire (DTSQ) were assessed at baseline and after treatment with the study drugs and scored. Data show estimated mean differences of the scores in SIDD minus SIRD participants and are presented as means with 95% confidence intervals at baseline and after treatment with semaglutide or dapagliflozin. The number of female (f) and male (m) participants are indicated.

**COEQ1-19 refers to the items as described under the Patient-reported outcomes section in Methods.

***Item of DTSQ that assesses frequency of unacceptably high blood glucose, rated from 0 (never) to 6 (most of the time).
**** Item of DTSQ that assesses frequency of unacceptably low blood glucose, rated from 0 (never) to 6 (most of the time).

**Supplementary Table 6. Overall treatment satisfaction score between SIDD and SIRD groups at baseline and after treatment with the study drugs***			
	**SIDD (n=100; 26 women, 74 men)**	**SIRD (n=83; 26 women, 57 men)**	**Mean differences****
**Overall treatment satisfaction at baseline**	25.18±6.49	27.61±6.44	-2.44 (-4.30 to -0.55)
**Overall treatment satisfaction after semaglutide (n=81; 25 women, 56 men)**	30.81±5.19	31.68±4.97	-0.86 (-3.07 to 1.36)
**Overall treatment satisfaction after dapagliflozin (n=102; 27 women, 75 men)**	27.83±6.26	30.18±5.43	-2.35 (-4.69 to -0.02)
**Change of treatment satisfaction relative to baseline in response to semaglutide*****	5.93 (3.40 to 8.45)	5.22 (2.97 to 7.47)	0.70 (-2.77 to 4.18)
**Change of treatment satisfaction relative to baseline in response to dapagliflozin*****	2.40 (0.37 to 4.44)	2.41 (0.29 to 4.52)	0 (-3.02 to 3.02)
**Mean difference of the change in response to semaglutide vs dapagliflozin******	3.52 (0.26 to 6.79)	2.82 (-0.33 to 5.96)	Interaction P value 0.7*****

*The items from the Diabetes treatment satisfaction questionnaire (DTSQ) were assessed at baseline and after treatment with the study drugs and scored. Total score of DTSQ (six items) ranges from 0 to 36, with larger values indicating higher satisfaction with treatment.
**Data show estimated mean differences of the scores in SIDD minus SIRD participants, as analyzed by two-sided t-test unadjusted for multiple comparisons, and are presented as means with 95% confidence intervals at baseline and after treatment with semaglutide or dapagliflozin. Data also show estimated mean differences in SIDD minus SIRD participants of the change of treatment satisfaction in response to each drug.
***Change of overall treatment satisfaction after treatment with semaglutide or dapagliflozin relative to baseline values with 95% confidence intervals, as analyzed by two-sided t-test.
****Mean difference of the change in treatment satisfaction in response to semaglutide minus dapagliflozin, analyzed by two-sided t-test.
*****The P value for the interaction term for study drug (semaglutide/dapagliflozin) and subgroup (SIDD/SIRD) with the change of treatment satisfaction in response to study drugs as dependent variable was analyzed using ANCOVA.

Supplementary Table 7. Change in clinical variables from diagnosis of diabetes to study inclusion.*				
	**SIDD well-regulated (n=58)**	**SIDD dysregulated (n=68)**	**SIRD well-regulated (n=65)**	**SIRD dysregulated (n=48)**
**Male – no (%)**	42 (72)	49 (72)	38 (75)	36 (58)
**Age at diagnosis – yr**	58 (56 to 60)	57 (55 to 60)	64 (62 to 65)	65 (63 to 67)
**Body mass index at diagnosis****	29.4 (28.2 to 30.7)	28.6 (27.4 to 29.7)	34.4 (33.1 to 35.7)	33.8 (32.4 to 35.2)
**HbA1c at diagnosis – mmol/mol**	94.1 (89.8 to 98.4)	100.5 (95.9 to 105.1)	55 (51.6 to 58.4)	55.8 (51.5 to 60.1)
**Fasting glucose at diagnosis – mmol/l**	11.4 (10.1 to 12.6)	12.3 (11.3 to 13.3)	7.1 (6.7 to 7.4)	7.8 (7 to 8.7)
**HOMA2-B at diagnosis**	51.0 (44.5 to 57.5)	47.0 (40.6 to 53.5)	150.6 (139.4 to 161.9)	139.6 (126.9 to 152.3)
**HOMA2-IR at diagnosis**	3.3 (2.8 to 3.9)	3.7 (3.2 to 4.1)	4.6 (4.3 to 5)	5.5 (4.6 to 6.3)
**Time between diagnosis and study inclusion – yr**	4.1 (3.2 to 4.9)	5.8 (4.8 to 6.8)	3.5 (2.7 to 4.2)	5.2 (4.3 to 6.1)
**Change in HbA1c between diagnosis and study inclusion – mmol/mol**	-45.4 (-49.9 to -40.9)	-35.3 (-40.7 to -29.9)	-6.9 (-10.3 to -3.5)	4.9 (0.5 to 9.3)
**Change in body mass index between diagnosis and study inclusion****	-1 (-1.6 to -0.5)	0.3 (-0.8 to -0.3)	-0.2 (-0.7 to 0.4)	0.5 (-0.3 to 1.2)
**Change in fasting glucose between diagnosis and study inclusion – mmol/l**	-4.3 (-5.7 to -2.9)	-2.1 (-3.2 to -1)	-0.4 (-0.8 to 0.2)	1.3 (0.4 to 2.2)
**Change in HOMA2-B between diagnosis and study inclusion**	34.8 (23.2 to 46.4)	4.7 (-3.8 to 13.1)	0.1 (-18.9to 19.1)	-42.9 (-55.9 to -29.9)
**Change in HOMA2-IR between diagnosis and study inclusion**	-1.1 (-1.6 to -0.5)	-0.8 (-1.2 to -0.5)	-0.5 (-0.8 to -0.2)	-1 (-2.1 to 0.2)

* Data are means with 95% CI. HbA1c denotes glycated hemoglobin, and HOMA2-IR and HOMA2-B denote homeostasis model assessment-2 estimates of insulin resistance and beta-cell function. Data at diagnosis were obtained from the All New Diabetics In Scania (ANDIS) cohort. The table also reports the change in variables from diagnosis to inclusion in this trial (before the addition of study drugs). Data are shown for study participants with SIDD or SIRD who at study inclusion had an HbA1c at 53 mmol/mol or below (termed well-regulated) and above 53 mmol/mol (termed dysregulated), respectively, on metformin monotherapy.

**The body-mass index is the weight in kilograms divided by the square of the height in meters.

Supplementary Table 8. Change in clinical variables in dysregulated participants in response to study drugs.*				
	**Semaglutide in SIDD (n=34)**	**Semaglutide in SIRD (n=28)**	**Dapagliflozin in SIDD (n=34)**	**Dapagliflozin in SIRD (n=20)**
**Male – no (%)**	24 (71)	20 (71)	25 (74)	16 (80)
**Age at study inclusion – yr**	63 (59 to 67)	72 (69 to 75)	65 (62 to 68)	68 (65 to 71)
**Baseline HbA1c in study – mmol/mol**	64.4 (61.7 to 67.2)	60.0 (57.9 to 62.2)	65.0 (61.9 to 68.2)	61.3 (58.4 to 64.1)
**Baseline BMI in study****	28.4 (27 to 29.7)	33.8 (31.7 to 35.9)	28.8 (26.9 to 30.7)	34.3 (31.4 to 37.1)
**Baseline HOMA2-B in study**	50.3 (40.2 to 60.4)	102.7 (85.4 to 120)	53.7 (45.8 to 61.6)	85.4 (69.1 to 101.7)
**Baseline HOMA2-IR in study**	2.8 (2.4 to 3.2)	4.7 (4 to 5.3)	2.9 (2.5 to 3.2)	4.4 (3.8 to 5.1)
**Change in HbA1c during study – mmol/mol**	-17.4 (-20.2 to -14.6)	-14.6 (-17.3 to -11.9)	-7.1 (-9.7 to -4.6)	-7.5 (-10.3 to -4.8)
**Change in BMI during study****	-1.9 (-2.3 to -1.6)	-2.5 (-3.1 to -1.9)	-1.1 (-1.4 to -0.9)	-1.8(-2.1 to -1.5)
**Change in HOMA2-B during study**	69.1 (49.9 to 88.3)	91 (72.4 to 109.6)	22.4 (14.1 to 30.7)	22.2 (11.9 to 32.6)
**Change in HOMA2-IR during study**	0.2 (0.2 to 0.6)	-0.1 (-0.5 to 0.5)	-0.4 (-0.6 to -0.2)	-0.6 (-1.2 to 0.1)
**Final HbA1c in study – mmol/mol**	47.5 (45.1 to 49.9)	45.3 (43.4 to 47.2)	57.9 (55.5 to 60.3)	54.4 (50.9 to 58.0)
**Final BMI in study****	26.5 (25 to 27.9)	31.4 (29.4 to 33.4)	27.7 (25.9 to 29.5)	32.5 (29.8 to 35.2)
**Final HOMA2-B in study**	119.3 (96.7 to 142)	193.6 (165.1 to 222.2)	75.9 (65.9 to 86.0)	107.6 (93.1 to 122.1)
**Final HOMA2-IR in study**	2.9 (2.4 to 3.5)	4.6 (3.8 to 5.4)	2.5 (2.1 to 2.9)	3.8 (3.2 to 4.4)

* Data are means with 95% CI. HbA1c denotes glycated hemoglobin, and HOMA2-IR and HOMA2-B denote homeostasis model assessment-2 estimates of insulin resistance and beta-cell function. The table shows data in participants who were dysregulated (HbA1c above 53 mmol/mol) on metformin monotherapy at study inclusion. Data from baseline and final visit as well as the change in variables from baseline to final visit are reported for participants with SIDD or SIRD who were randomized to semaglutide or dapagliflozin.

**The body-mass index is the weight in kilograms divided by the square of the height in meters.

Supplementary Table 9. Change in clinical variables in well-regulated participants in response to study drugs.*				
	**Semaglutide in SIDD (n=29)**	**Semaglutide in SIRD (n=29)**	**Dapagliflоxin in SIDD (n=29)**	**Dapagliflozin in SIRD (n=36)**
**Male – no (%)**	21 (72)	14 (48)	21 (72)	24 (83)
**Age at study inclusion – yr**	64 (61 to 67)	66 (62 to 70)	61 (57 to 65)	68 (66 to 70)
**Baseline HbA1c in study – mmol/mol**	49.2 (48.1 to 50.3)	48.0 (46.7 to 49.3)	48.7 (47.3 to 50.1)	48.3 (47.5 to 49.1)
**Baseline BMI in study****	28.4 (26.4 to 30.4)	34.8 (33.2 to 36.3)	29.5 (27.8 to 31.2)	33.6 (31.6 to 35.6)
**Baseline HOMA2-B in study**	88.2 (70.1 to 106.3)	133.6 (119.5 to 147.7)	83.5 (73.8 to 93.3)	153.5 (125.2 to 181.7)
**Baseline HOMA2-IR in study**	2.6 (2.1 to 3.1)	3.8 (3.4 to 4.2)	2.3 (2.0 to 2.7)	4.2 (3.8 to 4.5)
**Change in HbA1c during study – mmol/mol**	-8.4 (-10.0 to -6.7)	-6.6 (-8.2 to -4.9)	-1.9 (-3.2 to -0.6)	-0.1 (-1.4 to 1.2)
**Change in BMI during study****	-2.0 (-2.6 to -1.4)	-2.3 (-3.0 to -1.7)	-1.2 (-1.4 to -0.9)	-1.0 (-1.3 to -0.8)
**Change in HOMA2-B during study**	62.8 (41.7 to 83.8)	49.0 (32.2 to 65.8)	18.6 (9.7 to 27.6)	3.6 (-19.9 to 27.2)
**Change in HOMA2-IR during study**	0.0 (-0.3 to 0.3)	0.1 (-0.3 to 0.4)	-0.1 (-0.4 to 0.2)	-0.3 (-0.6 to 0.0)
**Final HbA1c in study – mmol/mol**	40.1 (38.6 to 41.7)	40.3 (38.8 to 41.8)	46.9 (45.2 to 48.7)	48.6 (46.9 to 50.2)
**Final BMI in study****	26.4 (24.4 to 28.4)	32.4 (31.0 to 33.8)	28.3 (26.7 to 30.0)	32.5 (30.5 to 34.6)
**Final HOMA2-B in study**	151.0 (119.7 to 182.2)	182.6 (162.4 to 202.9)	102.2 (90.2 to 114.2)	157.1 (138.9 to 175.3)
**Final HOMA2-IR in study**	2.6 (2.2 to 3.0)	3.8 (3.3 to 4.4)	2.2 (1.8 to 2.7)	3.9 (3.6 to 4.2)

* Data are means with 95% CI. HbA1c denotes glycated hemoglobin, and HOMA2-IR and HOMA2-B denote homeostasis model assessment-2 estimates of insulin resistance and beta-cell function. The table shows data in participants who were well-regulated (HbA1c at 53 mmol/mol or below) on metformin monotherapy at study inclusion. Data from baseline and final visit as well as the change in variables from baseline to final visit are reported for participants with SIDD or SIRD who were randomized to semaglutide or dapagliflozin.

**The body-mass index is the weight in kilograms divided by the square of the height in meters.

**Supplementary Table 10. Association between baseline variables and change of HbA1c in response to the drugs.***				
	**Semaglutide**		**Dapagliflozin**	
**Baseline variable****	**Beta**	**P pvalue**	**Beta**	**P value**
**Age – yr**	0.02	ns	0	ns
**Body mass index*****	0.09	ns	0.26	0.006
**HbA1c – mmol/mol**	-0.78	7E-23	-0.64	1E-14
**HOMA2-B**	0.49	1E-6	0.35	0.0003
**HOMA2-IR**	-0.05	ns	0	ns
**GAD antibody titer kE/l******	0	ns	0	ns

* Data from linear regression with baseline variables as the independent variable and change of HbA1c in response to semaglutide or dapagliflozin as the dependent variable. Standardized beta coefficients and P values (two-sided, unadjusted for multiple comparisons) from each regression analysis are presented. ns denotes non-significant P values (above 0.05).

** The variables corresponding to the clinical measures used for cluster designation.

*** The body-mass index is the weight in kilograms divided by the square of the height in meters.

**** Analyzed by ELISA from RSR Limited, Cardiff, UK,

Supplementary Table 11. Linear regression of baseline traits and the change of HbA1c in response to study drugs*		
**Independent variable**	**Beta coefficient of the association between independent variable and the change of HbA1c in response to semaglutide**	**Beta coefficient of the association between independent variable and the change of HbA1c in response to dapagliflozin**
**HbA1c**	**-0.63 (P=7E-23)****	**-0.41 (P=1E-14)****
**Age**	0.012	-0.003
**Sex**	-0.43	1.7
**Body mass index**	0.12	**0.27 (P=0.006)****
**Diabetes duration**	**-0.52 (P=0.02)****	**-0.52 (P=0.0006)****
**Systolic blood pressure**	0.008	**-0.09 (P=0.048)****
**Diastolic blood pressure**	-0.03	-0.07
**LDL**	0.28	0.08
**Triglycerides**	-0.91	0.41
**Estimated glomerular filtration rate**	-0.08	**-0.12 (P=0.01)****
**Urinary albumin/ creatinine index**	0.16	-0.18
**Fasting glucose**	**-2.2 (P=1E-12)****	**-1.7 (P=2E-11)****
**Glucose 120 minutes**	-0.96	-0.82
**HOMA2-B**	**0.78 (P=1E-6)****	**0.37 (P=0.0003)****
**HOMA2-IR**	-0.28	-0.50
**Insulin sensitivity index**	1.1	-0.18
**Disposition index**	**0.03 (P=1E-6)****	**0.01 (P=0.008)****
**Time in range**	**19.5 (P=2E-9)****	**14.3 (P=5E-6)****
**Coefficient of variance of glucose**	7.7	12.9

*Linear regression using each indicated baseline trait as independent variable and the change of HbA1c in response to semaglutide or dapagliflozin as the dependent variable. The unstandardized beta coefficients from each of the regression analyses are shown disaggregated for semaglutide and dapagliflozin, respectively.

**Beta coefficients with $P<0.05$ for the regression term are indicated in bold.
