# Supplementary material for: Randomized open-label trial of semaglutide and dapagliflozin in patients with type 2 diabetes of different pathophysiology
Source: Nat Metab. 2024 Jan 4;6(1):50–60. doi: 10.1038/s42255-023-00943-3 (PMC10822775; doi:10.1038/s42255-023-00943-3)
Supplement: Supplementary file 2 — Reporting Summary [file 42255_2023_943_MOESM2_ESM.pdf]

nature portfolio

Corresponding author(s): Rosengren Anders

Last updated by author(s): Aug 26, 2023

Reporting Summary

Nature Portfolio wishes to improve the reproducibility of the work that we publish. This form provides structure for consistency and transparency in reporting. For further information on Nature Portfolio policies, see our Editorial Policies and the Editorial Policy Checklist.

Statistics

For all statistical analyses, confirm that the following items are present in the figure legend, table legend, main text, or Methods section.

n/a	Confirmed	
☐	☒	The exact sample size (*n*) for each experimental group/condition, given as a discrete number and unit of measurement
☒	☐	A statement on whether measurements were taken from distinct samples or whether the same sample was measured repeatedly
☐	☒	The statistical test(s) used AND whether they are one- or two-sided<br>*Only common tests should be described solely by name; describe more complex techniques in the Methods section.*
☐	☒	A description of all covariates tested
☐	☒	A description of any assumptions or corrections, such as tests of normality and adjustment for multiple comparisons
☐	☒	A full description of the statistical parameters including central tendency (e.g. means) or other basic estimates (e.g. regression coefficient) AND variation (e.g. standard deviation) or associated estimates of uncertainty (e.g. confidence intervals)
☐	☒	For null hypothesis testing, the test statistic (e.g. *F*, *t*, *r*) with confidence intervals, effect sizes, degrees of freedom and *P* value noted<br>*Give P values as exact values whenever suitable.*
☒	☐	For Bayesian analysis, information on the choice of priors and Markov chain Monte Carlo settings
☒	☐	For hierarchical and complex designs, identification of the appropriate level for tests and full reporting of outcomes
☒	☐	Estimates of effect sizes (e.g. Cohen's *d*, Pearson's *r*), indicating how they were calculated

Our web collection on statistics for biologists contains articles on many of the points above.

Software and code

Policy information about availability of computer code

Data collection	No software was used
Data analysis	Statistical analyses were performed using SPSS (version 26, IBM) or R 4.1.0. The package XGBoost version 1.6.0.1 was used in R 4.1.0.

For manuscripts utilizing custom algorithms or software that are central to the research but not yet described in published literature, software must be made available to editors and reviewers. We strongly encourage code deposition in a community repository (e.g. GitHub). See the Nature Portfolio guidelines for submitting code & software for further information.

Data

Policy information about availability of data

All manuscripts must include a data availability statement. This statement should provide the following information, where applicable:

- Accession codes, unique identifiers, or web links for publicly available datasets
- A description of any restrictions on data availability
- For clinical datasets or third party data, please ensure that the statement adheres to our policy

Source data for main figures and Extended Data figures are appended with the paper. Further data requests should be submitted to the corresponding author (at anders.rosengren@gu.se). Access to anonymized data will be granted following review (time frame <20 office days) to ensure compliance with relevant ethical and legal considerations. The study protocol is appended with the paper and available online.

Research involving human participants, their data, or biological material

Policy information about studies with human participants or human data. See also policy information about sex, gender (identity/presentation), and sexual orientation and race, ethnicity and racism.

Reporting on sex and gender	The study enrolled both women and men, and the results are applicable to both women and men. Gender distribution is presented for data on enrollment, treatment allocation, discontinuation and adverse effects. Data on gender distribution has also been added to all figures and tables. In addition, the results of the primary outcome are presented disaggregated by gender. Gnder was determined by self-report and the social security number (no discrepancies).
Reporting on race, ethnicity, or other socially relevant groupings	The study includes participants living in southern Sweden in both rural and urban areas and a wide distribution of socioeconomic background.
Population characteristics	Participants had type 2 diabetes and were 18 years or above (age span of enrolled participants was 35-86 years)
Recruitment	Participants were recruited via letters or advertisements and they responded to invitation by self-selection. The self-selection procedure may result in a selection of individuals that are not entirely representative of the full population of patients with type 2 diabetes. The randomized treatment allocation should however minimize any systematic differences between randomization groups.
Ethics oversight	The protocol was approved by the Swedish Ethical Review Authority (2020-01353) and the Medical Products Agency (EUDRA-CT 2020-000109-33).

Note that full information on the approval of the study protocol must also be provided in the manuscript.

Field-specific reporting

Please select the one below that is the best fit for your research. If you are not sure, read the appropriate sections before making your selection.

☒ Life sciences ☐ Behavioural & social sciences ☐ Ecological, evolutionary & environmental sciences

For a reference copy of the document with all sections, see nature.com/documents/nr-reporting-summary-flat.pdf

Life sciences study design

All studies must disclose on these points even when the disclosure is negative.

Sample size	The study was designed to have 80% power to detect a treatment effect between the clusters, assuming that the true treatment effect was 3 mmol/mol. This applied to both dapagliflozin and semaglutide. The standard deviation of change in HbA1c over 6 months is 4.9 mmol/mol (as observed in the ANDIS cohort). At alpha 0.05, at least 86 SIDD and 86 SIRD patients were required, and we planned to recruit totally 100 SIDD and 100 SIRD patients.
Data exclusions	The full analysis set includes all participants who have at least one measurement of HbA1c after randomization, independent of compliance, duration of participation or potential dose reduction of semaglutide from 1.0 mg to 0.5 mg. Missing data were not imputed.
Replication	This was a clinical trial and as all trials has to be replicated in the future
Randomization	The randomization was generated by independent statisticians using a computer-based block randomization algorithm with balanced blocks. Randomization was stratified for SIDD and SIRD, respectively, to get an approximate distribution of semaglutide and dapagliflozin in a 1:1 ratio in both SIDD and SIRD. Allocation was concealed (via sealed envelopes) to both participants and study personnel until end of the first visit, after measurements of baseline variables had been completed. Thus, the generation of the random sequence, participant enrolment by study personnel, and the allocation to randomization groups were clearly separated. After randomization, the assignment was open-label.
Blinding	No blinding was possible since one drug was oral and one an injectable. It was therefore obvious, after treatment allocation, to both participants and staff (who also provided injection instructions to those randomized to semaglutide), which treatment the participant was receiving.

Reporting for specific materials, systems and methods

We require information from authors about some types of materials, experimental systems and methods used in many studies. Here, indicate whether each material, system or method listed is relevant to your study. If you are not sure if a list item applies to your research, read the appropriate section before selecting a response.

Materials & experimental systems

n/a	Involved in the study	
☒	☐	Antibodies
☒	☐	Eukaryotic cell lines
☒	☐	Palaeontology and archaeology
☒	☐	Animals and other organisms
☐	☒	Clinical data
☒	☐	Dual use research of concern
☒	☐	Plants

Methods

n/a	Involved in the study	
☒	☐	ChIP-seq
☒	☐	Flow cytometry
☒	☐	MRI-based neuroimaging

Clinical data

Policy information about clinical studies

All manuscripts should comply with the ICMJE guidelines for publication of clinical research and a completed CONSORT checklist must be included with all submissions.

Clinical trial registration	NCT04451837
Study protocol	Appended with the paper
Data collection	Clinical Studies Sweden, Forum South, Lund University Hospital, Lund, Sweden from August 2020 to 2021
Outcomes	The primary variable was the change from baseline in HbA1c. HbA1c was analyzed according to International Federation of Clinical Chemistry (IFCC) standard by a Capillary 3 TERA Hemoglobin A1c Kit. The secondary variables were the change from baseline in BMI, waist circumference, urinary albumin/creatinine index, blood pressure, LDL cholesterol, triglycerides, HOMA2-B, HOMA2-IR, disposition index, insulin sensitivity index and glucose at 0 and 120 minutes measured from the OGTT in SIDD versus SIRD patients using intraindividual comparisons. Via continuous glucose monitoring for two weeks both before and at 3 months of intervention, the change in glucose variability (estimated as the coefficient of variance of glucose), average glucose concentration and time in range (3.9-10 mmol/l) from baseline was also analysed. Patient-reported outcomes (Control of Eating Questionnaire and Diabetes treatment satisfaction questionnaire) were analysed at baseline and after treatment. In addition, plasma sodium, potassium, albumin as well as creatinine and cystatin C (both used to calculate estimated eGFR) were analysed as safety variables.
