# Supplementary material for: Randomized open-label trial of semaglutide and dapagliflozin in patients with type 2 diabetes of different pathophysiology
Source: Nat Metab. 2024 Jan 4;6(1):50–60. doi: 10.1038/s42255-023-00943-3 (PMC10822775; doi:10.1038/s42255-023-00943-3)
Supplement: Supplementary file 3 — Study protocol and statistical analysis plan. [file 42255_2023_943_MOESM3_ESM.pdf]

Study protocol and statistical analysis plan

This supplement contains the following items:

1. Original protocol, final protocol, summary of amendments.
2. Original statistical analysis plan (no amendments were done)

Study protocol

Semaglutide and dapagliflozin in diabetic patients with different pathophysiology

**Version number:**	**2** (DIAB1)
**Date:**	**22 April 2020**
**EUDRA-CT number:**	**2020-000913-33**
**Sponsor and PI**	**Anders Rosengren**

STUDY ADMINISTRATION

Table 1. Study administration

**Function**	**Responsible person**
Sponsor and Principal Investigator	Anders Rosengren, MD PhD Professor<br>Endokrinologiska kliniken Skånes Universitetssjukhus, Region Skåne /<br>Institutionen för neurovetenskap och fysiologi, Box 432, 40530 Göteborg<br>Tel: 070-5316704<br>Email: anders.rosengren@gu.se
Study location	Prövningsenheten, Skåne University hospital, Region Skåne, Sweden

ABBREVIATIONS

AE	Adverse Event
ALP	Alkaline phosphatase
ALAT	Alanine aminotransferase
ANDIS	All New Diabetic In Skåne
AR	Adverse Reaction
ASAT	Aspartate aminotransferase
AUC	Area Under Curve
BMI	Body Mass Index
CRF	Case Report Form
DSUR	Development Safety Update Report
eGFR	Estimated glomerular filtration rate
GCP	Good Clinical Practice
GLP1	glucagon-like peptide 1
HbA1c	Glycosylated haemoglobin A1c
ICH	International Conference on Harmonisation
OGTT	Oral glucose tolerance tes
PRO	Patient-reported outcomes
SAE	Serious Adverse Event
SGLT2	sodium-glucose cotransporter 2
SIDD	Severe Insulin-Deficient Diabetes
SIRD	Severe Insulin-Resistant Diabetes
SUSAR	Suspected Unexpected Serious Adverse Reaction
T2D	Type 2 diabetes
WHO	World Health Organization

SPONSOR AND PRINCIPAL INVESTIGATOR SIGNATURE

It is my responsibility that this protocol contains all essential parts to conduct the study. I am aware of my responsibility that the personnel working with the study are informed about the protocol and other relevant study-related information and are properly trained. The study will be conducted in accordance with the study protocol, the informed consent, ICH GCP, the Declaration of Helsinki and relevant national and international laws and regulations. I am also aware that quality control of the study will be conducted by monitor and potentially also by inspections.

Anders Rosengren	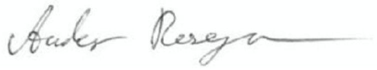	2020-04-22
Sponsor and Principal Investigator	Signature	Date

SYNOPSIS

**Study name**	Semaglutide and dapagliflozin in diabetic patients with different pathophysiology
**Version number**	2
**EudraCT number**	2020-000913-33
**Sponsor and Investigator**	Anders Rosengren
**Background and purpose**	Current anti-diabetic treatment fails to stop the progressive course of the disease. Recent studies have revealed a surprisingly high variability in the diabetic phenotype. We therefore propose that anti-diabetic treatment should ideally target the underlying pathophysiology of each individual patient. We will therefore test whether the effect of two approved anti-diabetic drugs differs between individuals at different ends of the pathophysiological spectrum: 1) patients with poor insulin secretion, here termed SIDD and 2) patients with high insulin resistance, here termed SIRD. The study may open up a new avenue for more precise treatment of diabetic patients that would be of immediate clinical relevance.
**Primary objective**	The primary objective is to study whether the anti-diabetic effect of semaglutide and dapagliflozin, respectively, differs between patients with poor insulin secretion and high insulin resistance, respectively. The primary endpoint will be the intraindividual change of HbA1c in response to semaglutide or dapagliflozin relative to baseline in the two patient groups.
**Secondary objectives**	The secondary endpoints will be the effect of semaglutide and dapagliflozin, respectively, on BMI, waist circumference, PRO, urinary albumin/creatinine index, blood pressure, blood lipids, disposition index, glucose sensitivity, insulin secretory rate, insulin sensitivity index and glucose at 0 and 120 minutes measured from the OGTT in the two patient groups.
**Study design**	This is a randomized open-label parallel arm phase II trial. We will recruit 200 patients with HbA1c ≥48 mmol/mol on metformin monotherapy. Half of them will have poor insulin secretion and half will have high insulin resistance but relatively good insulin secretion. The patients will be randomized (open-label) to receive semaglutide or dapagliflozin for six months in addition to metformin.
**Study population**	Patients with type 2 diabetes
**Number of subjects**	200 (up to 250 in total to cover for replacement recruitment in cases of withdrawal)
**Inclusion criteria**	• Diabetes mellitus based on prior documentation or treatment with anti-hyperglycemic medication or diagnosed according to the WHO criteria (random plasma glucose >11.1 mmol/L or fasting glucose >7.0 mmol/L or HbA1C ≥6.5%) and disease characteristics typical for SIDD or SIRD according to the ANDIS clustering

	• Ongoing metformin therapy with constant dose the last three months<br>• Age 18 years or above<br>• HbA1c ≥48 and <91 mmol/mol<br>• Women who are not postmenopausal and who have not undergone surgical sterilization must have no current pregnancy, which will be assessed by pregnancy test, must take precautions to avoid pregnancy throughout the study and for 4 weeks after intake of the last dose and must be willing to use highly effective birth control methods. Methods that can achieve a failure rate of less than 1% per year when used consistently and correctly are considered as highly effective birth control methods.<br>• Willingness to take injectable and oral medication<br>• Written informed consent
**Exclusion criteria**	• Type 1 diabetes, LADA, MODY, secondary diabetes or history of diabetic ketoacidosis<br>• Anti-diabetic treatment other than metformin within 90 days prior to randomization or changed metformin dose within 90 days prior to randomization<br>• Known acute cardiovascular event, e.g. transient ischemic attack, stroke, acute coronary syndrome, decompensated heart failure, coronary by-pass surgery or other coronary vessel intervention within 90 days prior to screening.<br>• Heart failure NYHA class IV<br>• History of acute or chronic pancreatitis<br>• Known liver cirrhosis<br>• Blood pressure above 170/110 mm Hg<br>• A level of aspartate aminotransferase (ASAT) or alanine aminotransferase (ALAT), ALP or bilirubin of more than three times the upper limit of the normal range<br>• Current chronic daily treatment with an oral steroid at a dose equivalent to oral prednisolone ≥10 mg (e.g., betamethasone ≥1.2 mg, dexamethasone ≥1.5 mg, hydrocortisone ≥40 mg)<br>• Pregnancy or breast-feeding<br>• Known galactose intolerance, total lactase deficiency or glucose-galactose malabsorption.<br>• Estimated glomerular filtration rate <45 ml/min/1,73 m^2 or unstable or rapidly progressing renal disease<br>• Participant unable to understand the study information herself or himself<br>• Involvement in the planning and/or conduct of the study<br>• Participation in other clinical trial which may affect the outcome of the present study<br>• Any condition or treatment that in the judgment of the investigator makes it difficult or unsafe to participate in the study.
**Study medication**	Dapagliflozin (Forxiga) tablets à 10 mg once daily for 6 months

	Semaglutide (Ozempic) s.c. injections 0.25 mg once weekly for four weeks, 0.5 mg once weekly for four weeks, and finally 1.0 mg once weekly throughout the study, total treatment duration 6 months
**Primary variable**	Change from baseline in HbA1c
**Secondary variables**	The secondary variables include BMI, waist circumference, PRO, urinary albumin/creatinine index, blood pressure, blood lipids, disposition index, glucose sensitivity, insulin secretory rate, insulin sensitivity index and glucose at 0 and 120 minutes measured from the OGTT.<br><br>As an exploratory part of the study, we will also analyse proinsulin, glucagon, GLP-1, ASAT, ALAT, ALP and bilirubin, glucose variability, average postprandial blood glucose and time in range as measured by continuous glucose monitoring, blood and urine metabolites and proteins as well as gut bacteria abundance.
**Duration of study**	June 2020 – June 2022

CONTENTS

PAGE
STUDY ADMINISTRATION 2
SPONSOR AND PRINCIPAL INVESTIGATOR SIGNATURE 4
SYNOPSIS 5
CONTENTS 8
1. BACKGROUND AND RATIONALE 10
2. AIMS OF THE STUDY 11
2.1 Primary objective 11
2.2 Secondary objectives 11
2.3 Exploratory objectives 11
2.4 Primary efficacy variable 11
2.5 Secondary and exploratory efficacy variables 12
2.6 Safety variables 12
2.7 Other variables that are not study outcomes 12
3. STUDY DESIGN AND PROCEDURES 12
3.1 Overall study design 12
3.2 Screening visit 15
3.3 Visit 1 (randomization visit) 17
3.4 Between visits 18
3.5 Visit 2 18
3.6 Visit 3 19
3.7 Questionnaire 20
3.8 Blood sampling and biobank 21
3.9 Physical measures 21
3.10 Stool samples 22
3.11 Urine samples 22
3.12 Glucose monitoring 22
3.13 Number of participants 23
3.14 End of trial 23
3.15 Time plan 23
4. SELECTION AND RECRUITMENT OF STUDY PARTICIPANTS 25
4.1 Study criteria 25
4.2 Recruitment 27
4.3 Inclusion 29

5. STUDY COMPOUND 30
5.1 Semaglutide 30
5.2 Dapagliflozin 31
5.3 Rescue medication 32
5.4 Randomization 33
5.5 Packaging, labelling and handling of study medication 33
5.6 Compliance 34
6. STATISTICS 37
6.1 Statistical analyses 37
6.2 Demographics and baseline characteristics 37
6.3 Efficacy analyses 37
6.4 Sample size 38
7. DATA MANAGEMENT 39
7.1 Quality control, data and sample handling 39
7.2 Documentation / Case Report From 40
7.3 Data handling after study 40
7.4 Monitoration 41
8. HANDLING OF ADVERSE EVENTS 42
8.1 Definitions 42
8.2 Reporting and documentation of adverse events 43
9. ETHICAL ASPECTS 46
9.1 Risks - benefits 46
9.2 Study information and ethical permits 48
9.3 Insurance 48
10. REFERENCES 49

1. BACKGROUND AND RATIONALE

An alarming 300 million people have type 2 diabetes (T2D), and the number is expected to exceed half a billion by 2030.[1] Current treatment strategies fail to stop the progressive course of the disease.[2] As a consequence, the disease causes severe complications in the kidneys, eyes and the cardiovascular system, making T2D one of the greatest threats to human health.

Clinical guidelines recommend metformin as initial therapy to all patients but emphasize the need for subsequent personalized treatment with additional drugs.[3] Although this sounds attractive, it is currently implemented on trial-and-error fashion and the concept as such has in fact not been examined systematically and with scientific rigor in a real-world-situation.

We have recently analyzed 9,000 diabetic patients of the ANDIS (All New Diabetics In Skåne) cohort, which highlighted four clusters of T2D patients, each with different characteristics and risk of complications.[4] Two of these clusters are particularly aggressive: one has been coined **SIDD** (**S**evere **I**nsulin-**D**eficient **D**iabetes) and features low age at onset, low BMI and poor insulin secretion; the other, termed **SIRD** (**S**evere **I**nsulin-**R**esistant **D**iabetes), presents at higher age and associates with high BMI and high insulin resistance. Some patients are 'archetypes' of a specific cluster, whilst others have more mixed phenotype.

This sheds new light on the mounting problem of T2D by emphasizing the high variability of the pathophysiology and providing a new tool to distinguish individuals at different ends of the pathophysiological spectrum. Importantly, it leads us to propose that anti-diabetic treatment should ideally target the underlying pathophysiology of each individual patient.

As a starting point we will study whether glucagon-like peptide 1 (GLP1) receptor agonists and inhibitors of the sodium-glucose cotransporter 2 (SGLT2) have different effects on glucose control in patients with SIDD and SIRD characteristics, respectively. These are the only classes of anti-diabetic drugs with proven cardiovascular benefits[3], and it is urgent to get improved knowledge on how to use them as effectively as possible. We will focus on SIDD and SIRD, which enables us to test the feasibility of tailored treatment in a more specific setting (poor insulin secretion and high insulin resistance, respectively). If successful, it may pave the way for combinatorial tailored therapy that is applicable also to patients with more mixed disease phenotype.[13]

Because of their cardiovascular benefits, GLP1 receptor analogues and SGLT2 inhibitors have gained increased use, despite high costs, and they are currently recommended as second-line treatment after metformin.[3] However, it is currently unknown to what extent their effect depends on the underlying pathophysiological characteristics.

Interestingly, our preliminary data from treatment registries coupled with ANDIS show that GLP1-stimulatory drugs, when prescribed in routine care, produce greater glucose-lowering effect in patients who cluster within SIDD compared with SIRD (p=0.0001 for HbA1c reduction, χ^2 test; n=288 of which 219 are SIDD and 69 SIRD patients).

In light of these observations, we will test the hypothesis that patients with SIDD experience greater therapeutic benefit from the GLP1 receptor agonist semaglutide than SIRD patients. We will also study the effect of the SGLT2 inhibitor dapagliflozin in the two patient groups. Dapagliflozin lowers blood glucose by decreased renal glucose reabsorption and reduced

weight.[3] The compound has also been shown to restore dedifferentiated β-cells and improve insulin secretion, presumably indirectly via reduced hyperglycaemia.[5] It is, however, currently unclear to what extent its effect is influenced by pathophysiological features (too few patients on SGLT2 inhibitors were included in the registry to enable analysis).

2. AIMS OF THE STUDY

2.1 Primary objective

The primary objective is to study whether the anti-diabetic effect of semaglutide and dapagliflozin, respectively, differs between patients with SIDD and SIRD characteristics. The primary endpoint will be the intraindividual change from baseline in HbA1c (ΔHbA1c in mmol/mol) in response to semaglutide or dapagliflozin in SIDD versus SIRD patients, which will be analysed using an ANCOVA model with a term for the exposure and an interaction term for the exposure and subgroup.

2.2 Secondary objectives

The secondary endpoints will be the effect of semaglutide and dapagliflozin, respectively, on BMI, waist circumference, PRO (see section 3.7), urinary albumin/creatinine index, blood pressure, blood lipids, disposition index, glucose sensitivity, insulin secretory rate, insulin sensitivity index and glucose at 0 and 120 minutes measured from the OGTT in SIDD versus SIRD patients using intraindividual comparisons.

2.3 Exploratory objectives

By continuous glucose monitoring for two weeks both before and at 3 months of intervention we will assess glucose variability, average postprandial glucose and time in range.

We will, as an exploratory part of the study, also measure proinsulin, glucagon, GLP-1, liver parameters and blood and urine metabolites and proteins of relevance to cardiovascular and metabolic diseases. The metabolite and protein data will be compared between treatment groups to identify potential markers to predict treatment response and glucose control. Moreover, stool samples will be obtained for subsequent sequencing and analysis of bacterial abundance to study how gut microbiota is affected by treatment and how it predicts treatment response and glucose control. These exploratory analyses aim to open up for potential follow-up studies.

2.4 Primary efficacy variable

The primary variable is change from baseline in HbA1c.

2.5 Secondary and exploratory efficacy variables

The secondary variables include BMI, waist circumference, PRO (see section 3.7), urinary albumin/creatinine index, blood pressure, blood lipids, disposition index, glucose sensitivity, insulin secretory rate, insulin sensitivity index and glucose at 0 and 120 minutes measured from the OGTT.

Exploratory variables are proinsulin, glucagon, GLP-1, ASAT, ALAT, ALP and bilirubin, glucose variability, average postprandial blood glucose and time in range as measured by continuous glucose monitoring, blood and urine metabolites and proteins as well as gut bacteria abundance.

2.6 Safety variables

Blood pressure, pulse rate, plasma sodium, potassium, albumin as well as creatinine and cystatin C (both used to calculate eGFR) will be used as safety variables to monitor volume depletion or changes in kidney function during the study. This will be especially important for patients co-treated with dapagliflozin and diuretics.

Patients who are excluded because of that criterion will be referred to follow-up at the appropriate responsible physician with additional blood pressure measurements and addition of anti-hypertensive medication in line with clinical routines. These patients can be re-screened at a later stage and be included if blood pressure has been appropriately reduced to meet the study criteria. The procedure for follow-up of blood pressure also pertains to patients who are excluded for other reasons than blood pressure but have a blood pressure above 140/90 at screening.

Patients who meet the study criteria and are included but have a blood pressure above 140/90 at any of the visits will be instructed to re-examine their blood pressure either at a primary care unit or at the study site. We will be in close contact with those subjects to ensure that antihypertensive medication is initiated/intensified if blood pressure is repeatedly above 140/90 (defined as two additional measurements on two separate days). Treatment will then be administered using primarily ramipril, followed by amlodipine and metoprolol, but the choice should also be informed by the overall clinical picture, patient preferences and concomitant medication and diseases. If the patient has previously been prescribed anti-hypertensive compound(s) those may be dose-increased based on the overall picture. Initiation or dose elevation of diuretics should be avoided, in particular for patients on dapagliflozin.

2.7 Other variables that are not study outcomes

In addition to the outcome and safety variables we will collect data on age, gender, time since diabetes diagnosis, diabetic complications, metformin dose, Hb (to ensure that changes in Hb do not confound HbA1c measures) and lifestyle data (see Questionnaire description in section 3.7).

3. STUDY DESIGN AND PROCEDURES

3.1 Overall study design

The trial is a clinical phase II study that will be open-label with fixed stratification variables (SIDD and SIRD) to analyze if the response to anti-diabetic drugs differs between patients with distinct pathophysiology, as captured by SIDD and SIRD. The compounds used are semaglutide and dapagliflozin, which will be randomized to patients of each subgroup using a parallel group design.

The clusters (SIDD and SIRD) will be used as a practical tool to distinguish individuals who are at different ends of the pathophysiological spectrum.

We will recruit 200 patients from the ANDIS registry with HbA1c ≥48 mmol/mol on metformin monotherapy (**Fig. 1**). Half of them will have SIDD and half will have SIRD characteristics. The patients will be randomized (open-label) to receive semaglutide or dapagliflozin for six months in addition to metformin.

We will recruit participants on metformin monotherapy with stable dose for the last three months. Metformin dose at inclusion (as prescribed by their regular physician) is maintained throughout the study; we will correct for metformin dose in the analyses. Patients randomized to add semaglutide will receive injection training at the study site and inject 0.25 mg subcutaneously once weekly during the first four weeks, followed by 0.5 mg weekly for the subsequent four weeks and finally 1.0 mg weekly throughout the study. Those randomized to dapagliflozin will receive 10 mg orally once daily in addition to metformin. The participants will attend a screening visit followed by three study visits at 0, 3, 6 months. At the first and last study visit they will undergo an OGTT. HbA1c will be measured at all study visits. Moreover, stool samples will be collected and extra blood and urine will be stored for analyses of metabolites and proteins as exploratory parts of the study. During two periods of the study (before randomization and after three months of treatment) the participants will wear a Libre Pro sensor for continuous glucose monitoring for 2 weeks (**Fig. 1**).

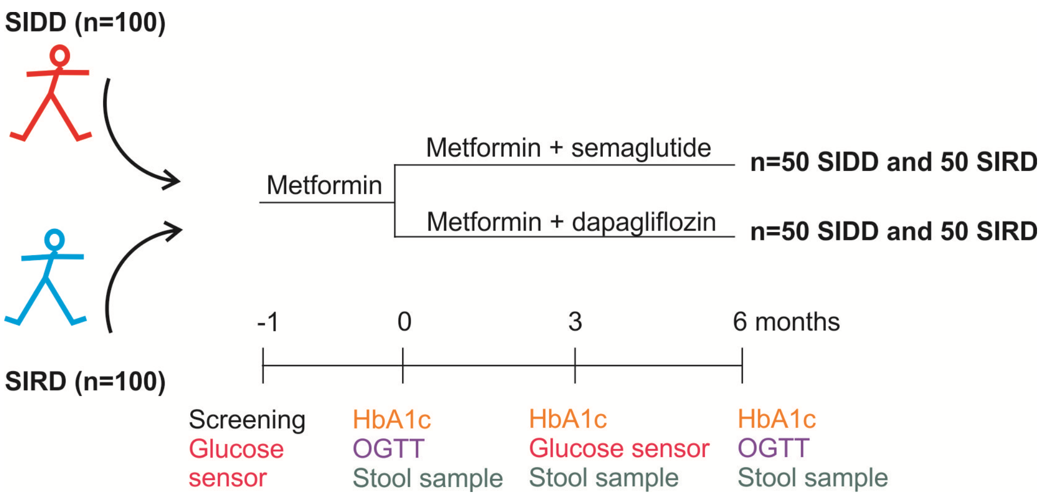

Figure 1. *Schematic of the intervention.*

Table 1. *Summary of study procedures*

	Screening	Visit 1	Telephone contact	Visit 2	Visit 3
**ID check**	X	X		X	X
**Informed consent**	X				
**Patient receives screening ID**	X				
**Patient receives study ID**		X			
**Capillary HbA1c**	X				
**Inclusion & exclusion criteria**	X				
**Medical data recorded**	X				
**Non-fasted venous sampling**	X			X	
**Pregnancy test premenopausal women**	X				
**Hand out glucose sensor**	X			X	
**Hand out tube for stool sample**	X	X		X	
**Hand out tube for urine sample**	X			X	
**Note in medical record**	X	X		X	X
**Note changes in disease/medication**		X	X	X	X
**Collect fecal samples**		X		X	X
**Collect urine sample**		X			X
**Collect glucose sensor**		X			X
**Record AEs**		X	X	X	X
**Height, weight, waist circumference**		X			X
**Blood pressure and pulse rate**	X	X		X	X
**Fasted venous sampling**		X			X
**OGTT**		X			X

**Questionnaire**		X			X
**Randomisation**		X			
**Hand out medication instruction**		X			
**Hand out medication for 3 months**		X		X	
**Hand out diary**		X			
**Follow up compliance**			X	X	X
**Book times for visits 2&3 and tele call**		X			
**Hand out study card to patients**		X			
**Collect diary**					X
**Collect and count remaining medication**					X
**End of study**					X

3.2 Screening visit

Participants who are interested in taking part in the study will attend a screening visit (**Table 1**).

After receiving oral and written information and signing the informed consent the patient will undergo screening process. As a first step the capillary HbA1c will be measured and analysed on-site (appr. 6 min). Only those who have HbA1c ≥48 and <91 mmol/mol proceed to further control of study criteria, and if they are fulfilled we sample venous blood. This procedure is to make screening as effective as possible, as we from previous studies with patients recruited from ANDIS have experienced that HbA1c is the study criteria that is most likely to result in screening failure. Re-screening will be allowed once. A physician will obtain written informed consent and check study criteria while capillary and venous sampling will be done by research nurses or authorized biomedical scientists.

The following will be done at the screening visit after study information and informed consent:

- The participant gets a screening number
- Capillary HbA1c is analysed on-site (appr. 6 min)

If capillary HbA1c ≥48 and <91 mmol/mol we proceed with the following (at the same visit):

- Additional inclusion and exclusion criteria are checked (by physician)
- Relevant medical data are collected, including time since diabetes diagnosis, diabetic complications (retinopathy, neuropathy, nephropathy and other complications) and current medication (name and dose).
- Blood pressure and pulse rate are measured.
- Venous blood samples are drawn for analysis of eGFR, Na, K, ASAT, ALAT, ALP, bilirubin. Total blood volume is estimated to 5 ml.
- A pregnancy test is done for premenopausal women.
- The participant is scheduled for visit 1, which will be within one month after the screening visit.
- The participant receives a Freestyle Libre glucose sensor pad.
- The participant receives tubes for urine and stool samples and is instructed how to sample at home.

If any necessary information is unknown to the patient at screening, e.g. on relevant medical data, it may be complemented by asking for the patient's permission to obtain the information from the medical journal.

After screening visit:

- If eGFR, ASAT, ALAT, ALP or bilirubin do not meet the study criteria the patient will be contacted and study participation cancelled.
- A letter is sent to the patient's physician managing his/her diabetes with information on the study and that the patient will be randomized to semaglutide or dapagliflozin. The letter will also state that we will be responsible for the diabetes medication while the patient participates in the study and that the antidiabetic medication should not be modified during that time.

Data from the screening visit are recorded on the CRF, except for blood data analyzed at the hospital laboratory, which will be entered into a secure database. The participant receives a screening number (S1001, S1002, etc.). The code list for screening number and personal ID is kept at the study site. Only subjects who have signed the informed consent form should be included on the logs and receive a screening number.

Screening failures

Those who attend the screening visit, sign the informed consent but are not eligible to participate will continue to be managed by their regular physician. If necessary, the subject will be referred to relevant healthcare clinic to follow up abnormalities discovered at the screening visit. All reasons for screening failure should be documented, incl. those that occur between screening visit and visit 1. See also 2.6 for follow-up of blood pressure abnormalities.

3.3 Visit 1 (randomization visit)

The participants are recommended not to conduct intense physical activity or drink alcohol 24 h before the visit. They should be fasting since 10pm the previous day. Nicotine users should not have used nicotine the same day.

The following will be done at visit 1:

- Personal ID is checked
- Information on any changes of medication or disease status since last visit is collected
- The participant receives a study ID (different from the screening ID)
- The stool sample brought from home is received and frozen
- The samples with morning urine is collected. One tube is sent for analysis of albumin/creatinine index at the local hospital laboratory and 2x20 ml is frozen for subsequent analyses of urine biomarkers.
- The Libre sensor pad is collected.
- Recording of AEs
- Length, weight, waist circumference, blood pressure and pulse rate are measured
- Venous fasting blood samples are drawn for analysis of HbA1c, Hb, eGFR, Na, K, ASAT, ALAT, ALP, bilirubin, and lipids (HDL, LDL, total cholesterol, triglycerides). Two extra tubes (2x3,5 ml) are stored for subsequent analysis of metabolites and proteins that are relevant for T2D. Total blood volume is estimated to 15 ml.
- An oral glucose tolerance test (OGTT) is conducted at which patients drink 75 g glucose dissolved in water. Venous blood samples are drawn at 0, 30, 60, 90 and 120 minutes for glucose, insulin and c-peptide. An extra plasma tube (total 3.5 ml) is stored from each time point during the OGTT for subsequent analysis of metabolites and proteins. Total blood volume at each time point is 7 ml, making a total of 35 ml for the OGTT.
- Participants complete a questionnaire on lifestyle habits and PRO (see 3.7).
- Any fresh stool sample is collected.
- The patient is randomized to receive semaglutide or dapagliflozin.
- The patient receives study medication for the coming 3-month period and information about the medication. The patient also receives a diary to mark each time they have taken a dose. Patients randomized to semaglutide will receive injection instructions.
- A note is made in the medical record about the visit.
- The patient receives a tube for stool sampling to be collected before visit 2
- A time is scheduled for telephone contact 2 weeks later, for visit 2 three

months later and for visit 3 six months later. The patient also receives a card about the study to show in contacts with healthcare professionals, including the regular physician and nurse managing their diabetes.

Data from the visit are recorded on the CRF, except for blood data analyzed at the hospital laboratory, which will be entered into a secure database.

Subsequent visits should ideally be scheduled on the same weekday as visit 1. If that is not possible, e.g. because of public holidays or unavailability of the participant to come on an assigned day, the subsequent visit will be scheduled as soon as possible three months after the previous visit. Reasonable measures should be taken to schedule the visits at three month intervals but a delay of up to three weeks is acceptable to accommodate for travels, sudden illness etc. The participant will be reminded by email or mobile text message before next visit.

3.4 Between visits

The participant continues taking all their regular treatment during the study. They are instructed not to change their metformin dose or general lifestyle habits (e.g. overall dietary pattern) during the study and contact us before any such changes are done.

The study team will contact the participants by phone appr. 2 weeks after the initiation of the treatment to record any AEs and to check compliance. Adverse events are noted, and appropriate follow-up is initiated. Reasonable efforts should be undertaken to get in contact with the participants by calling back if no reply and/or sending emails. Participants could contact us at their own initiative in case they have any suspected side effects of the treatment or experience problems with the semaglutide injections. Patients may also be contacted by phone at additional time points to follow-up on adverse events, compliance or injection problems.

Patients who at Visit 1 have total cholesterol ≥7.0 mM, LDL cholesterol ≥5.0 mM or triglycerides ≥5.6 mM will be followed up with an additional test of fasting lipid levels. If any value is still above these limits appropriate medication will be initiated/dose-increased. Atorvastatin should be the primary drug of choice but the overall clinical picture, patient preferences and concomitant medication and diseases should also be taken into account. See also point 2.6 for follow-up of blood pressure abnormalities.

3.5 Visit 2

The participants do not need to be fasting at visit 2 (visits can be done in the afternoon).

The following will be done at visit 2:

- ID is checked
- Information on any changes of medication or disease status since last visit is collected

- Stool samples are received and frozen
- Recording of AEs
- Blood pressure and pulse rate are measured
- Venous blood samples are drawn for analysis of HbA1c, eGFR, albumin, Na, K. Two extra tubes (2x3,5 ml) are stored for subsequent analysis of metabolites and proteins that are relevant for T2D. Total blood volume is estimated to 15 ml.
- Any fresh stool sample is collected.
- The participant receives a Freestyle Libre glucose sensor pad to wear for two weeks.
- Patient brings remaining study medication and diary, which are assessed. Any discrepancies are discussed to reinforce compliance, and the participant receives medication for the next 3-month period.
- The participant receives tubes for stool and urine to be sampled before visit 3
- The time scheduled for visit 3 three months later is checked.
- A note is made in the medical record about the visit.

Data from the visit are recorded on the CRF, except for blood data analyzed at the hospital laboratory, which will be entered into a secure database.

3.6 Visit 3

The participants are recommended not to conduct intense physical activity or drink alcohol 24 h before the visit. They should be fasting since 10pm the day before. Nicotine users should not have used nicotine the same day. The last dapagliflozin tablet should be taken the evening before Visit 3 and the last semaglutide injection 3 days before Visit 3 (see also 5.1 and 5.2).

The following procedures will be done at visit 3:

- ID is checked
- Information on any changes of medication since last visit is collected
- Stool samples are received and frozen
- The samples with morning urine is collected. One tube is sent for analysis of albumin/creatinine index at the local hospital laboratory and 2x20 ml is frozen for subsequent analyses of urine biomarkers.
- The Libre sensor pad is collected
- The diary is returned
- Remaining study medication is returned, counted and stored temporarily before destruction

- Recording of AEs
- Weight, waist circumference, blood pressure and pulse rate are measured
- Venous fasting blood samples are drawn for analysis of HbA1c, Hb, eGFR, Na, K, ASAT, ALAT, ALP, bilirubin, and lipids (HDL, LDL, total cholesterol, triglycerides). Two extra tubes (2x3,5 ml) are stored for subsequent analysis of metabolites and proteins that are relevant for T2D. Total blood volume is estimated to 15 ml.
- An oral glucose tolerance test (OGTT) is conducted at which patients drink 75 g glucose dissolved in water. Venous blood samples are drawn at 0, 30, 60, 90 and 120 minutes for glucose, insulin and c-peptide. An extra plasma tube (total 3.5 ml) is stored from each time point during the OGTT for subsequent analysis of metabolites and proteins. Total blood volume at each time point is 7 ml, making a total of 35 mL for the OGTT.
- Participants complete a questionnaire on lifestyle habits and PRO (see 3.7).
- A note is made in the medical record about the visit.
- End of study for the participant.

Data from the visit are recorded on the CRF, except for blood data analyzed at the hospital laboratory, which will be entered into a secure database.

3.7 Questionnaire

The participants are instructed not to make any major lifestyle changes during the study (e.g. major changes of dietary pattern, initiation of weight reduction programmes etc.). We will assess lifestyle habits by a questionnaire, which will assess the following:

- Physical activity using the short IPAQ (international physical activity questionnaire), which is a commonly used scale to assess physical activity.
- Dietary habits using items that have been validated in Stockholm health questionnaire 2010 (Stockholms folkhälsoenkät 2010).
- Tobacco and alcohol, by validated items previously used by the national Health questionnaires in Sweden (Folkhälsoinstitutets nationella folkhälsoenkäter).

We will also assess patient-reported outcomes by

- Diabetes Treatment Satisfaction Questionnaire[6]
- Control of Eating Questionnaire (CoEQ).[7] It comprises 21 items designed to assess the intensity and type of food cravings, as well as subjective sensations of appetite and mood. We will use 19 of its items to assess the subscales of craving control, positive mood, craving for savoury and craving for sweet.

This is of relevance because both dapagliflozin and semaglutide may affect appetite.[16]

The questionnaires take appr. 15-20 minutes to complete.

3.8 Blood sampling and biobank

The Investigator should make an assessment of the available results with regard to clinically significant abnormalities. The laboratory reports should be signed and retained as source data for laboratory variables.

Venous HbA1c, Hb, creatinine, cystatin C, Na, K, albumin, ASAT, ALAT, ALP, bilirubin, lipids (HDL, LDL, total cholesterol, triglycerides), insulin as well as urine albumin/creatinine index are analysed at the accredited hospital laboratory at Skåne University hospital. Venous blood glucose is analysed directly via a Hemocue glucose reader. Capillary HbA1c at screening visit is analysed by an Alere Afinion AS100 reader according to the manufacturer's instructions. All these samples are analysed within the same day as the visit and destroyed immediately. The extra blood tubes that are obtained for later analyses will be handled according to the Biobank law and regulations. The samples will be pseudonymized using a study ID. The code list for study ID/personal ID will be stored securely and separately at the study site and after the study by the sponsor to prevent unauthorized persons to access them.

If a patient withdraws consent to the use of donated biological samples, the samples will be destroyed, and the action documented. If samples are already analysed, the sponsor is not obligated to destroy the results of this research.

3.9 Physical measures

Blood pressure and pulse rate

Blood pressure and pulse rate will be measured using a standardized cuff adapted to the size of the patient's arm after the patient has been sitting and resting for least 5 minutes. The subject should not talk during the measurement.

Body weight, height and waist circumference

The patient's height will be recorded at Visit 1 in centimeters, with no shoes.

The patient's body weight will be recorded in kilograms, to 1 decimal place, with light clothing and no shoes and recorded at Visits 1 and 3.

The waist circumference will be measured in cm as the minimal abdominal circumferences located midway between the lower rib margin and the iliac crest. The subject should be standing with arms down their side and feet together. The tape should touch the skin but not compress soft tissue and twists in the tape should be avoided. The subject should be asked to breathe normally and the measurement should be taken when the subject is breathing out gently.

3.10 Stool samples

Stool sampling is optional. The patient will receive a tube and written instructions on how to sample. Briefly, they are instructed to collect a stool sample within 24 h before their study visit, store the sample at room temperature and bring it to the study site. They may also bring it to the study site up to two days after their visit as long as it is less than 24 h since sampling. The sample will be frozen at the study site. The date and time of receiving the sample and the time of freezing should be entered into a log.

They are also asked to collect a second stool sample at the study visit, if possible, for immediate freezing. This is to enable analysis of a subset of samples that have been frozen immediately to study the difference in quality between samples stored at up to 24 h at room temperature and those frozen immediately. That will be important for evaluating the quality of microbiota data from this and subsequent studies.

After the study the stool samples will be sequenced to analyzed gut bacterial abundance.[8] Also gut bacterial metabolites will be analysed. The aims of these analyses are to

- study any differences in gut bacterial composition that distinguish SIDD and SIRD patients
- study the effect of the drugs on gut bacterial composition
- predict drug responders and non-responders
- predict disease deterioration and complications (see 7.3).

3.11 Urine samples

The patient receives tubes for urine sampling to sample first morning urine and bring to visit 1 and 3. From that, a 10 ml aliquot is sent for analysis of albumin/creatinine index at the local hospital laboratory and 2x20 ml is frozen for subsequent analyses of urine biomarkers.

3.12 Glucose monitoring

A Freestyle Libre Pro, provided by Abbott, will be used to monitor glucose continuously in study participants. The study personnel will put a pad on the participant's arm (this has to be done by healthcare personnel for the Pro sensor as opposed to the standard Libre sensor) and the participant wears the pad during two weeks. There is no connected reader (as opposed to the standard Libre sensor), which is an advantage in this case because the patients will not receive any feedback on their glucose values, which could otherwise lead to dietary adjustments and recording biases. Included participants wear the sensor for two weeks from the screening visit, then take it off themselves and return it to the study center, either by a posted envelope or at their subsequent visit. They do not wear any sensor between Visit 1 and 2. At Visit 2, when they have been on study medication for three months, they receive a new

sensor, wear it for two weeks and return it to the study center.

Each sensor has a serial number that will be connected with a study ID and recorded in a separate document at the study site. The sensor can store the equivalent of two weeks' recordings and holds the data indefinitely. Upon return the sensor data is transferred from the pad to a Freestyle Libre Pro Reader, which is available at the study center, and is then transferred (by a USB cable) to a stationary computer. Glucose variability, time in range and postprandial glucose concentrations will after the study be analyzed and compared before and after treatment for each individual. Study ID will be used in these analyses.

If a study participant is already wearing a standard Freestyle Libre sensor (e.g. prescribed by healthcare) he/she will be allowed to wear it during the study but will still receive a Freestyle Libre Pro sensor by us to collect study data.

If the Libre Pro sensor pad comes to loose earlier than two weeks, the patient can either return the pad (although it will contain data from a shorter time period) or visit us to receive a replacement pad. This will be determined case by case by the study personnel depending on the timing of subsequent visits. If a patient experiences skin reactions to the sensor he/she will be instructed to contact us. The pad may in those cases be removed at the discretion of the Investigator.

3.13 Number of participants

200 participants will participate in the study; 100 with SIDD and 100 with SIRD. They will be randomized to semaglutide or dapagliflozin at a 1:1 ratio. Participants with both SIDD and SIRD will be recruited simultaneously.

In case of withdrawals or exclusions during the study we will recruit new participants to get the required number of participants for the full analysis set (see also 6.4).

3.14 End of trial

End of trial is defined by last subject, last visit. For this type of trial we have not identified any stopping criteria for the entire study as it is using approved drugs. Should the study anyway be terminated prematurely, e.g. in case of force majeure, the PI will immediately inform the participants and ensure proper follow-up. Relevant authorities will also be notified, latest within 15 days. The decision on termination is taken by sponsor.

Latest 90 days after end of trial, the Medical Product Agency will be informed by a Declaration of End of Trial Notification.

3.15 Time plan

First recruitment activities: June 2020

First subject in: August 2020

Last subject in: December 2020

Last subject last visit: June 2021

Final report: latest June 2022

4. SELECTION AND RECRUITMENT OF STUDY PARTICIPANTS

4.1 Study criteria

Inclusion criteria

- Diabetes mellitus based on prior documentation or treatment with anti-hyperglycemic medication or diagnosed according to the WHO criteria(random plasma glucose >11.1 mmol/L or fasting glucose >7.0 mmol/L or HbA1C ≥6.5%) and disease characteristics typical for SIDD or SIRD according to the ANDIS clustering
- Ongoing metformin therapy with constant dose the last 90 days
- Age 18 years or above
- HbA1c ≥48 and <91 mmol/mol
- Women who are not postmenopausal and who have not undergone surgical sterilization must have no current pregnancy, which will be assessed by pregnancy test, must take precautions to avoid pregnancy throughout the study and for 4 weeks after intake of the last dose and must be willing to use highly effective birth control methods. Methods that can achieve a failure rate of less than 1% per year when used consistently and correctly are considered as highly effective birth control methods. Such methods include:
  - combined (estrogen and progestogen containing) hormonal contraception associated with inhibition of ovulation: oral, intravaginal or transdermal
  - progestogen-only hormonal contraception associated with inhibition of ovulation: oral, injectable or implantable
  - intrauterine device
  - intrauterine hormone-releasing system
  - bilateral tubal occlusion
  - vasectomised partner
  - sexual abstinence
- Willingness to take injectable and oral medication
- Written informed consent

Exclusion criteria

- Type 1 diabetes, LADA, MODY, secondary diabetes or history of diabetic ketoacidosis
- Anti-diabetic treatment other than metformin within 90 days prior to inclusion
- Known acute cardiovascular event, e.g. transient ischemic attack, stroke, acute coronary syndrome, decompensated heart failure, coronary by-pass surgery or

other coronary vessel intervention within 90 days prior to inclusion.

- Heart failure NYHA class IV
- History of acute or chronic pancreatitis
- Liver cirrhosis
- Blood pressure above 170/110 mm Hg
- Current chronic daily treatment with an oral steroid at a dose equivalent to oral prednisolone ≥10 mg (e.g., betamethasone ≥1.2 mg, dexamethasone ≥1.5 mg, hydrocortisone ≥40 mg)
- Pregnancy or breast-feeding
- Known galactose intolerance, total lactase deficiency or glucose-galactose malabsorption.
- Participant unable to understand the study information herself or himself, (information will only be available in Swedish and we have no interpreter resources)
- Involvement in the planning and/or conduct of the study
- Participation in other clinical trial which may affect the outcome of the present study
- Any condition or treatment that in the judgment of the investigator makes it difficult or unsafe to participate in the study.

Patients will be excluded and should not be randomized if the following are observed from laboratory tests analysed after screening visit:

- Estimated glomerular filtration rate <45 ml/min/1,73 m^2 or unstable or rapidly progressing renal disease
- A level of aspartate aminotransferase (ASAT) or alanine aminotransferase (ALAT), ALP or bilirubin of more than three times the upper limit of the normal range

<u>Criteria for withdrawal</u>

- The participant can withdraw from the study at any time without further motivation and without any consequences for his/her future treatment.
- The participant will be withdrawn should he/she develop any condition or begin taking any compound (apart from the study medication) that is part of the exclusion criteria. If blood pressure at any visit is above 170/110 the measurement will be repeated at least twice at two different days before withdrawal to ensure that it is not a chance finding.
- A participant can be withdrawn if he/she does not adhere to the procedures as specified in the protocol.
- If the participant experiences serious adverse events and there are reasons to

think that the event is related to the study, he/she will be immediately withdrawn and prompt action will be taken to assist the participant as necessary.

- Acute renal insufficiency or worsened chronic renal insufficiency (a decrease of eGFR of 15 mL/minute/1.73m^2 or greater compared with baseline) as verified by repeat eGFR values. The re-test should be scheduled within 4 days, whenever possible.

  If an unexpected, acute decline in kidney function is observed, the patient should be promptly evaluated. Urinary tract infection and urinary obstruction should be considered. Several drugs may cause a decline in kidney function, especially non-steroidal anti-inflammatory drugs (NSAID) and certain antibiotics such as trimethoprim. If any drug is suspected of causing or contributing to worsening kidney function, their use should be re-considered.

A patient who is withdrawn and have taken the study medication for at least 3 months with 80-120% compliance will be asked to attend a final visit, which will then follow the procedures outlined for Visit 3. We will inform them that any such visit is voluntary.

A patient who decides and/or is recommended by treating physician to permanently discontinue study medication, will be asked about the reason(s) and the presence of any AEs. AEs will be followed up according to clinical routine also for patients who are withdrawn (see also section 8.2). The primary reason for discontinuation of study medication must be specified in the CRF unless the patient declines to motivate the reason.

Female subjects must be instructed to notify the investigator immediately if they become pregnant during the trial.

All participants who are withdrawn will continue their regular medication and their ordinary physician will be notified as appropriate.

4.2 Recruitment

Patients with T2D will be recruited from ANDIS (Alla Nya Diabetiker I Skåne / Ethical approval nr. 584/2006), which registers diagnosed diabetic patients in Region Skåne (southern Sweden) since 2008 (http://andis.ludc.med.lu.se/).

Procedures in ANDIS

When a patient in Region Skåne is diagnosed with diabetes they are asked by their regular physician to be included in the ANDIS registry. Patients who want to be included sign an informed consent, and a blood sample is drawn for analysis of fasting glucose, C-peptide (measure of insulin secretion) and GAD antibodies (sign of autoimmune diabetes). These data as well as data on age, BMI, HbA1c, fasting glucose and basic clinical characteristics are stored within Region Skåne. Patients who are registered in ANDIS are managed by their regular physician. There are no follow-up visits in ANDIS, but on the ANDIS informed consent form patients are asked

whether they would like to be contacted for recruitment to other studies of relevance for diabetes. ANDIS is led by a steering group with representatives from academia and healthcare. Prof Leif Groop is scientific PI and founder of ANDIS. Anders Rosengren is clinically responsible for ANDIS since 2014.

Cluster analysis in ANDIS

In a recent collaboration, we have obtained data that suggest a need for a diagnostic refinement to better reflect the variable patient characteristics.[4] We performed a data-driven k-means cluster analysis of 9,000 diabetes patients in the ANDIS registry based on six variables measured at diagnosis: GAD antibodies (antibodies to pancreatic beta-cells), age, BMI, HbA1c, HOMA2-B (reflecting insulin secretion and calculated from glucose and c-peptide concentrations) and HOMA2-IR (reflecting insulin resistance and calculated from glucose and insulin concentrations). Four clusters of T2D patients were highlighted, each with different characteristics and risk of complications (a separate group corresponding to type 1 diabetes with elevated GAD antibodies and poor insulin secretion was also identified). The findings were replicated in three additional cohorts.[4]

Recruitment from ANDIS in this study

Here we will recruit patients with T2D who cluster within SIDD or SIRD in the ANDIS registry and who have accepted to be contacted with information on diabetes-related studies. We have previously recruited patients from ANDIS e.g to the studies "Randomized study with yohimbine in type 2 diabetes patients with or without a genetic risk variant" (EUDRA-CT 2010-018604-85, Ethical permit nr. 2011/587) and Effect of broccoli in type 2 diabetes (Ethical permit nr. 2015/395).

We will reach out to patients via 1) letters and 2) advertisements. For the letters, the PI or by him assigned person receives a list from the ANDIS database manager (Jasmina Kravic) with patients clustering within SIDD or SIRD. The list of potentially eligible patients is generated using coded data in the ANDIS database. The list is then uncoded (by Jasmina Kravic) in order to obtain the patients' personal ID so that we can reach them. This list is stored at a local server behind the hospital firewall. Next, letters are sent to these patients with the study information. If we do not get any response within one month a reminder letter may be sent out. We will also recruit via advertisements (see advertisement text as appendix). Patients who become interested in the study, either via letters or advertisements, go to a dedicated website, which has a simple booking interface. The website has the necessary IT security and the participants will not be able to see which other persons have booked a time (they will only see available and non-available slots). They enter their name, personal number, telephone number and email address and receive a confirmation email with instructions for the subsequent procedure. They can also contact us by email or phone if they have any questions.

The ANDIS registry covers a large number of diabetic patients in Skåne (more than 20,000 patients since appr. 100 primary care centres and 9 hospitals in Skåne are involved in ANDIS) but there are also patients with diabetes in the region who are not included in the ANDIS registry (exact numbers are difficult to estimate but based on

prevalence data there should be appr. 40,000 T2D patients in Skåne). These patients may still see the advertisement and become interested in the study. In that case they have to be registered in ANDIS in order for us to analyse their cluster assignment (to know if they have SIDD or SIRD or belong to any of the other three clusters that are not assessed in this study). For mathematical reasons, the patients need to be part of ANDIS as the cluster analysis is based on how they relate to other patients in terms of GAD antibodies, age, BMI, HbA1c, HOMA2-B and HOMA2-IR, using the ANDIS clustering algorithm as in ref. 4.

We will therefore check whether patients who sign up for a screening visit are already included in ANDIS and if so, whether they have SIDD or SIRD. Patients who are not in the ANDIS registry will be contacted and will be asked to be included in ANDIS. These patients will receive specific study information in which we explain that participation in the ANDIS registry is voluntary but a prerequisite to take part in this study. If they want to take part, they will book a time to a nurse at the Clinical Research Center, Skåne university hospital Malmö, for a visit according to the standard procedures in ANDIS. Once they have given informed consent, blood will be drawn and ANDIS data obtained for analysis of cluster assignment (see Procedures in ANDIS above). If they cluster within SIDD or SIRD they will be given the opportunity to attend a screening visit for the present study following the same procedure as all other patients. Patients in other clusters will not be able to take part in this study but will remain in ANDIS (unless they withdraw their ANDIS consent). We inform them beforehand that inclusion in ANDIS does not automatically mean that they can be enrolled in the present study as that depends on their cluster assignment and fulfilment of other study criteria. They are also informed that they can withdraw their consent to ANDIS or the present study at any point.

4.3 Inclusion

If the participants have given informed consent and fulfil all study criteria they will be formally included at visit 1. Included participants receive a study ID, which is different from the screening number. The code list for study ID and personal ID is kept with the Investigator at Skåne University hospital. Only coded data, based on the study ID, will be used for analyses.

5. STUDY COMPOUND

The patients will remain on the metformin dose they had at inclusion and are instructed not to change this dose during the study. They continue with their current medication throughout the study and all concurrent medication is entered on the CRF and any changes are documented. All background medication is considered to be non-investigational medicinal product. We have no placebo treatment in this study. The study medications are semaglutide and dapagliflozin, as described in more detailed below.

5.1 Semaglutide

Semaglutide (Ozempic, manufactured by NovoNordisk) is a human GLP-1 receptor agonist for once weekly subcutaneous administration.[3,9]

The start dose is 0.25 mg once weekly during the first four weeks, then 0.5 mg weekly during four weeks and finally 1.0 mg weekly throughout the study. Dose reduction to 0.5 mg will be allowed if the participant experiences unacceptable side effects at 1.0 mg. In those cases we will adjust for dose reduction in the analyses. If a dose is missed it should be taken as soon as possible or at least within 5 days after the missed dose after which the patients continue on their regular weekly schedule. If more than five days have elapsed the dose will be counted as a missed dose and the regular schedule continues.

The participant is instructed to adjust the administration schedule such that the last semaglutide dose is taken 3 days before Visit 3. This is to standardize administration in relation to the final OGTT.

It is injected subcutaneously in the abdomen, thigh or upper arm at any time of day, irrespective of meals. It should not be injected intravenously or intramuscularly.

Semaglutide preparations (both not in-use and in-use) must not be exposed to excessive heat or direct sunlight. Semaglutide preparations which have been frozen must not be used. It must not be used if it does not appear clear and colourless.

Storage conditions for semaglutide are as follows:

Not in-use:

- Store in a refrigerator (2°C to 8°C).
- Do not freeze.
- Protect from light.

In-use:

- Store below 30°C or in a refrigerator (2°C to 8°C).
- Use within 1 month.
- Do not freeze.
- Protect from light.

These instructions will be given to the patients.

The study site must ensure the availability of proper storage conditions and record and evaluate the temperature. The temperatures during storage should be monitored by a calibrated, stationary and continuously recording system. A temperature log must be kept to document storage within the right temperature interval and storage facilities should be checked frequently. Fifteen minutes outside the indicated range is negligible and allowed, and should not be recorded as a deviation.

Returned trial products (unused, partly used or used including empty packaging material) must be stored separately from non-allocated trial products before being sent for destruction.

No dose adjustment is necessary with regard to age, kidney or liver function.

Acute pancreatitis has been observed after the use of GLP1 receptor agonists.[3] The patients will be informed of the characteristic symptoms of acute pancreatitis and medication should be temporarily stopped if it is suspected and permanently stopped if it is verified. Patients with previous pancreatitis should not be included in the study (see 4.1 Exclusion criteria).

The most commonly reported side effects are gastrointestinal (nausea, diarrhoea and vomiting), typically mild to moderate and of short duration. In previous studies a total of 6.1% and 8.7% of patients interrupted their medication on 0.5 and 1.0 mg semaglutide, respectively, because of side effects, as compared with 1.5% in the placebo groups. The risk for hypoglycaemia is small in this study since the patients do not have concomitant insulin or sulfonylurea medication (the frequency of hypoglycaemia in previous studies was 0.001 cases per patient year). Erythema at the site of injection has been reported among 0.5% of the patients, typically of mild nature.

In subjects treated with GLP-1 receptor agonists AEs such as nausea, vomiting and diarrhoea may lead to significant dehydration and secondary acute renal impairment. Subjects with gastrointestinal AEs are recommended to drink plenty of fluids to avoid volume depletion. As a safety measure, markers of kidney function will be monitored throughout the trial.

Semaglutide has not been shown to affect the exposure or pharmacodynamics of warfarin. However, it is still recommended to assess PK-INR after initiation of semaglutide in patients on warfarin treatment. This will be handled by the investigator case-by-case.

5.2 Dapagliflozin

Dapagliflozin (Forxiga) is manufactured by AstraZeneca and will be provided as tablets of 10 mg.[3,10] The dose is the recommended clinical dose for type 2 diabetes patients. No dose adjustment is necessary with regard to kidney function, but the effect on glucose control is decreasing with reduced glomerular filtration rate (GFR). Estimated GFR (eGFR) below 45 ml/min/1,73 m^2 will therefore be an exclusion criterion and rapidly progressing renal insufficiency will be reason for withdrawal (see 4.1). Dose reduction is necessary if liver function is severely deteriorated, and severe liver disease (known cirrhosis) is therefore an exclusion criterion. No dose adjustment

is recommended based on age. The patients who are randomized to dapagliflozin will take one tablet daily for 6 months. It can be taken at any time of day with or without food. The patients are however instructed to take dapagliflozin in the evening three days prior to Visit 3. This is to standardize administration in relation to the final OGTT.

The tablets are to be stored at room temperature.

If the patient suffers from dehydration, e.g. because of a gastrointestinal infection, dapagliflozin medication should be temporarily paused until the volume loss has been corrected. The patients are recommended to contact us should this occur, and proper action is taken by the PI case by case. Temporary pause of treatment will also be considered if the patient develops pyelonephritis or urosepsis.

Rare cases of Fournier’s gangrene have been reported and the patients will be instructed to contact healthcare in case they develop edema or pain in the perineal region.

Dapagliflozin is not recommended during pregnancy, especially during the second and third trimester. We screen for pregnancy in premenopausal participants and recommend immediate contact with us in case of pregnancy.

These instructions are given in both oral and written form at the randomization visit.

Studies have not shown any increased frequency of hypoglycemic events when dapagliflozin is taken in combination with metformin (unless combined with sulfonylurea or insulin).[10] Urogenital infections are common side effects (4.7% compared with 3.5% with placebo in previous studies) and will be managed in accordance with clinical routines and the judgement of the Investigator. Usually, dapagliflozin treatment can continue in those cases.

The tablets contain lactose, and known galactose intolerance, total lactase deficiency or glucose-galactose malabsorption are therefore exclusion criteria.

If the patient misses a dose he/she should take only one tablet as usual the next day. Patients mark their daily intake in a diary, which is to be returned at the final visit.

5.3 Rescue medication

If the participant develops HbA1c ≥91 mmol/mol or diabetic ketoacidosis (see below for definition) rescue medication will be initiated in the form of injectable insulin. The participant will be asked to attend a study visit as soon as possible, which will then follow the procedures outlined for Visit 3. These patients will be included in the full analysis set but not in the per protocol analyses. These participants will then end the study and will be referred to healthcare for continued follow-up.

Diabetic Ketoacidosis (DKA) definition

A diagnosis of Diabetic Ketoacidosis should only be made in a clinical setting consistent with DKA (based on patient history, symptoms, and physical exam) and in the absence of more likely alternative diagnoses and causes of acidosis (such as lactic acidosis). The following biochemical data should support diagnosis:

• Ketonaemia ≥3.0 mmol/L and/or significant ketonuria (more than 2+ on standard urine sticks) and at least one of the following criteria suggesting high anion gap metabolic acidosis:

a) Arterial or Venous pH ≤7.3

b) Serum bicarbonate ≤18 mEq/L

c) Anion gap [Na – (Cl + HCO_3)] >10

5.4 Randomization

The randomization will be organized via an independent statistician using a computer-based block randomization algorithm with balanced blocks. Randomization will be stratified for SIDD and SIRD, respectively, so that we get an approximate distribution of 1:1 semaglutide:dapagliflozin in both SIDD and SIRD.

The randomization list will be numbered 1-200 for SIDD patients and 201-400 for SIRD patients with each number corresponding to either semaglutide or dapagliflozin. Sealed envelopes will be prepared for each participant with study ID printed on the outside. Each envelope contains a card informing about the assigned study medication. The envelope corresponding to the patient's study ID will be opened at visit 1 by the site staff (information is blinded to study personnel, Investigator and participant before randomization). The patient receives corresponding study medication.

After visit 1 the assignment is open-label. The study medication taken by each patient will be entered in the medical journal and in a letter to be sent to the physician normally managing the patient's diabetes.

The full randomization list (in a sealed envelope) is kept at the study site during the entire study until final analyses. No emergency envelopes are needed as the study is open-label and the assigned study medication is entered in the medical record.

If a participant is withdrawn during the study, that study ID will not be reused and the participant is not allowed to take part again.

5.5 Packaging, labelling and handling of study medication

Dapagliflozin (Forxiga) will be provided by AstraZeneca and semaglutide (Ozempic) will be provided by NovoNordisk using similar manufacturing, quality controls and packaging as for routine clinical use. The study medication will be sent to Tamro AB in Sweden, where the packages will be labelled with study-specific labels (in addition to the standard labelling of the drug package from the manufacturer).

Study labels will be prepared by Tamro AB in accordance with Good Manufacturing Practice (GMP) and local regulatory guidelines. The labels will fulfil GMP Annex 13 requirements for labelling. The labels will contain the following information:

Trial medication, study name and EudraCT number, name and contact details of Investigator and storage conditions.

Tamro AB will send study medication to the study site, where it will be stored under appropriate conditions. As participants become randomized they receive study medication in a quantity corresponding to the coming 3-month period. The study personnel manually write (with a ballpen) the participant's study ID on the packages that are handed out as well as the order of packages to be used (of relevance only for the dose escalation of semaglutide). The participant also receives written information about the study medication and a diary. In the diary, the study personnel write the date of the first dose to be taken and the participant then makes a note every time they take a dose. Participants randomized to semaglutide will receive injection instructions at visit 1.

<u>The participants randomized to semaglutide will receive the following:</u>

At visit 1:

1x Ozempic 0,25 mg, 1 x 4 doses

1x Ozempic 0,5 mg, 1 x 4 doses

1x Ozempic 1,0 mg, 1 x 4 doses

At visit 2:

3x Ozempic 1,0 mg, 1 x 4 doses

Participants who need additional medication, because of e.g. failure or misuse of injection pens or delayed visits, may at the discretion of the Investigator receive additional medication. Dose reduction to 0.5 mg will be allowed if the participant experiences unacceptable side effects at 1.0 mg.

<u>The participants randomized to dapagliflozin will receive the following:</u>

At visit 1:

1x Forxiga 1 x 98 tablets à 10 mg

At visit 2:

1x Forxiga 1 x 98 tablets à 10 mg

Participants who need additional medication, because of e.g. delayed visits may at the discretion of the Investigator receive additional medication. That will be provided as packages containing Forxiga 1 x 28 tablets à 10 mg.

5.6 Compliance

At each visit and telephone contact the investigator will remind the subject to adhere

to trial procedures described in the protocol.

Subject compliance will be assessed by monitoring of drug accountability. Prior to visit 2 and 3 the subject will be asked to return all used, partly used and unused trial products. The investigator must assess the amount of trial products returned compared to what was dispensed at the last dispensing visit and if a subject is discovered to be non-compliant, the investigator must inform the subject of the importance of taking trial product as directed.

Patients are also instructed to bring their diary to the visits. Patients judged to have questionable compliance (defined as taking less than 80% or more than 120% of the study medication) will continue in the study, but should be counselled on the importance of taking their medication as prescribed.

5.7 Costs and reimbursement

The participants receive the study medication without any cost. They will also receive travel reimbursement. This amount is subject to tax. Patients withdrawing from the study will receive payment for the visits that have taken place. Travel reimbursement will also be paid in cases of screening failure.

5.8 Destruction of study medication

The patients return any unused or partly used study medication at their final visit. The remaining medication is counted and then destructed via Tamro AB.

5.9 Treatment after study

All subjects should be assessed at end of study (Visit 3 or the latest visit for subjects who discontinue) concerning treatment effect. It should also be assessed if the subject would benefit from ongoing additional anti-diabetic medication in addition to metformin. Choice of treatment is based on current HbA1c, AEs and the patient's view and should then be prescribed for an adequate time by the Principal Investigator. In parallel, a formal letter of referral should be sent to the ordinary care giver at end of study describing the response to treatment and decisions made concerning future treatment until the responsible care giver once again has reasonable opportunity to resume responsibility for the patient. The Principal Investigator is responsible for the anti-diabetic treatment until the ordinary care giver has responded that s/he resumes responsibility.

In addition to the post-study anti-diabetic treatment as described above, if the patient at end of study is severely dysregulated in blood pressure or blood lipids, in the sense that blood pressure is above 140/90, total cholesterol ≥7.0 mM, LDL cholesterol ≥5.0 mM or triglycerides ≥5.6 mM, we will initiate rescue medication based on the considerations for treatment choice as described in 2.6 and 3.4, and send a referral to the appropriate responsible physician. The Principal Investigator is responsible for

rescue medication until the ordinary care giver has responded that s/he takes responsibility for follow-up and further treatment.

6. STATISTICS

6.1 Statistical analyses

We will use both full analysis set and per protocol analysis of the outcome variables. The full analysis set will include all participants who have at least one value after randomization (whether from visit 2 or 3) independent of compliance. Participants who are withdrawn prematurely from the study and have taken their medication for at least 3 months will be asked to attend a study visit if possible to measure study variables. That study visit will if possible follow the procedures of Visit 3, including an OGTT. If data from visit 3 are not available, data from visit 2 will be used.

Participants with data of the primary variable from all study visits and least 80% compliance overall and >80% during the last month will be included in the per protocol analysis.

6.2 Demographics and baseline characteristics

Demographic and baseline characteristics will be summarized, using frequency distributions and summary statistics based on the full data set, for each treatment group as well as for all patients combined.

Additional summaries of demographic and baseline characteristics may be performed for specific subgroups, including participants with HbA1c at 48 mmol/mol or above.

6.3 Efficacy analyses

The primary endpoint will be the intraindividual change from baseline in HbA1c (ΔHbA1c in mmol/mol) in response to semaglutide or dapagliflozin in SIDD versus SIRD patients, which will be analysed using an ANCOVA model with a term for the exposure and an interaction term for the exposure and subgroup. HbA1c at baseline will be used as a covariate. Thus, treatment, SIDD/SIRD assignment and the interaction will be analysed. In a supportive analysis we will also correct for metformin dose using a continuous measure of metformin dose (in mg). If the metformin dose, despite our instructions, has been changed during the study the participant will not be included in the PP analysis.

To better reflect the continuous nature of the disease variables, we will also include a measure of cluster centeredness, as individuals near the centre of a cluster, ‘archetypes’, are more likely to exhibit the typical characteristics of SIDD and SIRD than those at the periphery. We will therefore make a subanalysis based on HOMA-B, HOMA-IR, age, BMI and HbA1c (the variables used to cluster patients into SIDD and SIRD) to analyze how cluster centricity influences treatment efficacy.

The secondary variables will be analyzed similar to the primary efficacy variable. The secondary variables will be compared between between visit 1 and 3 to obtain intraindividual delta values for each participant. The intraindividual delta values will then be analysed across all subjects using independent t-tests.

Missing data will not be imputed.

6.4 Sample size

The standard deviation of ΔHbA1c is 4.9 mmol/mol over 6 months (as observed in the DIACT cohort). With 80% power at alpha=0.05, we need 43 SIDD and 43 SIRD patients to detect a significant treatment effect between the clusters, assuming that the true treatment effect is 3 mmol/mol.[11,12] This applies to both dapagliflozin and semaglutide We aim to recruit 50 individuals to each treatment arm, totally 100 SIDD and 100 SIRD patients. If a participant is withdrawn during the study and has taken the study medication for at least 3 months with 80-120% compliance, the participant will be asked to attend a final visit, which will then, if possible, follow the procedures outlined for Visit 3. If we have not obtained any outcome data for HbA1c for a patient, we will recruit new participants to get the required number of participants for the full analysis set.

7. DATA MANAGEMENT

7.1 Quality control, data and sample handling

All samples should be taken by adequately trained study personnel. The Investigator is to ensure that all personnel involved in the study is appropriately trained. All personnel in the study must have read the protocol in detail.

Routine blood samples will be analysed directly at the hospital's central laboratory. We calculate eGFR as the mean of the relative eGFR based on creatinine (using the revised Lund-Malmö estimating equation) and the relative eGFR based on Cystatin C (using the CAPA formula). Analyses of blood and urine metabolites and proteins from stored samples and stool samples will be made using special kits or equipment (e.g. mass spectrometry for metabolites, ELISA, RIA and arrays for proteins and sequencing for stool samples). These analyses aim to

- Analyse plasma c-peptide, proinsulin, glucagon and GLP-1 to assess beta-cell function and incretin levels in response to treatment in the different patient groups
- short-term identify biomarkers that distinguish SIDD and SIRD patients and predict drug responders and non-responders
- long-term, up to several years after the present study, predict disease deterioration and complications (see 7.3).

All data from CRF and routine blood analyses will be entered manually by specific study personnel into a secure database. Quality control procedures will be applied to each stage of data handling to ensure that all data are reliable and have been processed correctly.

Questionnaires will be given in paper format and the responses will be entered manually into the database by specific study personnel. Only study ID will be used to identify individuals. The questionnaire data will be analysed using numeric variables and summed into subscales where appropriate.[6,7,16]

Data from glucose sensors will be transferred from the reader to a local disc (no cloud-based storage) as text files. Data from exploratory variables (metabolite, protein and gut microbiota analyses) will be in electronic format using study ID.

The database is stored at local servers at Clinical Research Center, Skåne University hospital, behind a firewall. Only authorized personnel can access the data and all logins are traceable. Only specific IT staff has physical access to the server facility. All stored data will be coded using study ID and analyses are done using coded data. Only the sponsor or by him assigned persons will have access to these pseudonymised data.

The study information and informed consent form describe how data are handled and the procedure if a subjects wants to view, correct or remove data.

Data and samples may also be shared with academic or industrial partners outside the University of Gothenburg or Region Skåne. In that case, any data or samples shared

will be anonymized, i.e. the study ID will be removed so that data cannot be linked back the data to the personal ID.

7.2 Documentation / Case Report From

All study data collected in the study will be registered in a paper case report form (CRF). The participant is identified through his/her study ID.

All AE, SAE and SUSAR are classified by the investigator and noted in the CRF.

The investigator, sponsor, monitor and persons assigned by the investigator have access to the CRF.

Correction of CRF is done by manually changing the data, signing and dating the correction.

Data that are specific to the study and not relevant for the care of the participant do not have to be entered into the participant's medical record.

All data in the medical record must be in agreement with the CRF. The name of the study, date for informed consent, study medication, and date for end of treatment must be noted in the medical record. The participant's study ID does not have to be entered in the medical record as the study is not blinded.

CRF and the laboratory results (in paper form) constitute source data. A document of what is classified as source data will be in the Investigator File. The Investigator must ensure that all source data are accessible for monitoring and other quality control.

The investigator must keep a log of staff and a delegation of tasks list at site.

7.3 Data handling after study

Study document and source data will be archived for at least 10 years after study report. Source data in the hospital's medical records system will be stored according to local regulations.

Data will be published in peer-reviewed medical journals, at scientific conferences, for lay audience and in media and updated in the Eudra-CT database and clinicaltrials.gov within 1 year after end of trial. Only de-identified data based on averages will be used. Data from exploratory analyses may be published at a later stage than the report(s) describing the primary and secondary variables.

The protocol may be published in part or full in accordance with scientific standard.

Study participants may after last visit get to know their routine lab data collected during the study, including Hba1c, if so requested. The sponsor has however no obligation to report other study data, including e.g. analyses of blood metabolomics or gut microbiota, to individual participants. The study participants will after publication of the results receive a brief summary of the study outcome for their information.

The ANDIS registry is continuously updated with data on diabetic complications, medication and diabetes-relevant blood variables. Since all participants will also be part of ANDIS this opens up a valuable opportunity to verify whether blood, urine or

stool biomarkers collected in the study can predict long-term disease outcomes. We will therefore after the study prospectively request information from ANDIS on HbA1c, complications (retinopathy, nephropathy, neuropathy and cardiovascular diagnoses) as well as current diabetic medication (with a particular focus on those remaining on semaglutide/dapagliflozin). The aim of this is to identify biomarkers that can predict which patients 1) have increased risk for deteriorated glucose control, 2) have increased risk for specific complications and 3) respond best to semaglutide/dapagliflozin over time in case they continue using those compounds (see also 7.1). We will inform the participants that their study data will be compared with long-term disease outcomes in the ANDIS registry. They can at any time withdraw their consent to that. In that case, no prospective analyses will be done for that participant.

7.4 Monitoration

The study will be monitored by an independent monitor before the study begins, during the study conduct, and after the study has been completed, to ensure that the study is carried out according to the protocol and that data is collected, documented, and reported according to ICH-GCP and applicable ethical and regulatory requirements. Monitoring is performed as per the study's monitoring plan and is intended to ensure that the subject's rights, safety, and well-being are met as well as data in the CRF are complete, correct, and consistent with the source data.

Failure to follow the protocol, GCP or other regulations in a way that significantly affects or likely could affect the participants or the scientific value of the study should be reported to the Medical Product Agency within 7 days. It is the responsibility of the sponsor to make this decision. Smaller aberrations that do not affect the integrity or security of the participants or the scientific value of the study should be documented by the sponsor/investigator.

It is the responsibility of the Investigator to ensure that the monitor has access to the CRF, the medical record and original laboratory data etc. to ensure that source data are relevant, without violating the integrity of the participants. The sponsor and the monitor will make a risk-based monitoring plan and the sponsor will continuously follow up the plan. An agreement is signed before monitoration to ensure that the monitor is not disclosing personal data about the participants to any third party.

Authorized representatives of a regulatory authority may perform inspections at the center. The purpose of an inspection is to systematically and independently examine all study-related activities and documents, to determine whether these activities were conducted, and data were recorded, analyzed, and accurately reported according to the protocol, ICH GCP guidelines and any applicable regulatory requirements.

Major protocol changes are only possible by approved amendments to the ethics committee and/or the Medical Products Agency. Changes should be marked carefully and protocol version updated accordingly.

8. HANDLING OF ADVERSE EVENTS

8.1 Definitions

An **adverse event** (AE) is any untoward medical occurrence in a subject administered a medicinal product, and which does not necessarily have a causal relationship with this treatment.

An AE can therefore be any unfavourable and unintended sign (including an abnormal laboratory finding), symptom or disease temporally associated with the use of a product, whether or not considered related to the product.

An AE includes:

- A clinically significant worsening of a concomitant illness.
- A clinical laboratory abnormality which is clinically significant, i.e. an abnormality that suggests a disease and/or organ toxicity and is of a severity that requires active management. Active management includes active treatment or further investigations, for example change of medicine dose or more frequent follow-up due to the abnormality.

The following should not be reported as AEs:

- Pre-existing conditions, including those found as a result of screening or other trial procedures performed before exposure to trial product (pre-existing conditions should be reported as medical history or concomitant illness).
- Pre-planned procedures unless the condition for which the procedure was planned has worsened from the first trial related activity after the subject has signed the informed consent.

A **serious adverse event** (SAE) is an experience that at any dose results in any of the following:

- Death.
- A life-threatening experience.
- In-patient hospitalisation or prolongation of existing hospitalisation.
- A persistent or significant disability or incapacity.
- Congenital anomaly/birth defect
- Important medical events that may not result in death, be life threatening or require hospitalisation may be considered an SAE when - based on appropriate medical judgement - they may jeopardise the subject and may require medical or surgical intervention to prevent one of the outcomes listed in the definition of SAE.

A **Suspected Unexpected Serious Adverse Reaction** (SUSAR) is an SAE that has

not been documented or reported in previous studies with the drugs. All SAEs must be evaluated as to whether they are unexpectedly or expectedly related to the drug or not at all related.

8.2 Reporting and documentation of adverse events

Adverse Events will be collected from the signing of informed consent throughout the study until and including the last visit/contact.

Adverse events will be collected through spontaneously reporting and by telephone contact in between visits as well as by questions from study personnel at visits followed by documentation in the CRF, including intensity and causality. AEs identified during the study will be followed up by visits to the study site for blood sampling and clinical examination, telephone contacts or appropriate referrals based on the overall clinical picture. AEs should be followed up until the patient has recovered or is taken care of by other healthcare clinics.

Any unresolved AE during the study or at end of study (at visit 3 or earlier in case of withdrawal) will be followed up by up by the Investigator for as long as medically indicated. This will include visits and the study site for blood sampling and clinical examination, telephone contacts and/or appropriate referrals based on the overall clinical picture. Adverse events should be followed up until resolved or stabilized.

Any aberrant blood values discovered at the screening visit will not be considered as AEs and will be handled as appropriate, also for individuals who are not included.

Any change to a concomitant illness should be recorded during the trial. A clinically significant worsening of a concomitant illness must be reported as an AE

The following variables will be collected for each AE:

- AE (verbatim)
- Date of start and stop
- Maximum intensity
- Whether the AE is serious or not
- Causal relationship with study drug, graded as likely, possible or unrelated
- Action
- Outcome (resolved / not resolved)

In addition, the following variables will be collected for SAEs:

- Date AE met criteria for serious AE
- Date Investigator became aware of serious AE
- AE is serious due to
- Date of hospitalization

- Date of discharge
- Detailed description of AE.

Intensity will be graded according to the following rating scale:

• **Mild** (awareness of event but easily tolerated)

• **Moderate** (discomfort enough to cause some interference with usual activity)

• **Severe** (inability to carry out usual activity)

Causality is graded by the investigator as follows:

- **Likely related**, occurring within a reasonable time after administration of the intervention. It is unlikely that the event can be attributed to underlying disease or other drugs without it most likely being caused by the trial drug and its occurrence being reasonable in connection with the use of the trial drug.
- **Possibly related**, occurring within a reasonable time after administration of the intervention. The event may be explained by the trial drug and the onset is reasonable in connection with the use of the trial drug, but there is insufficient information to establish the relationship. The event can be explained by underlying disease or other drugs.
- **Unrelated**, i.e. unlikely to be related to the intervention and may be explained by other drugs or underlying disease.

Deterioration as compared to baseline or reference values in clinical variables measured as part of the protocol should only be reported as AEs if they fulfill any of the SAE criteria or are the reason for discontinuation of treatment with the study medication.

If deterioration in clinical variables is associated with clinical signs and symptoms, the sign or symptom will be reported as an AE and the associated laboratory result/vital sign will be considered as additional information.

Hyperglycemia is not considered an AE.

Hypoglycemic episodes or symptoms of hypoglycemia should only be reported in the CRF if the event fulfills the definition of a major hypoglycemic event (requiring external assistance due to severe impairment in consciousness or behavior) or the protocol criteria for an SAE.

Dapagliflozin has a modest diuretic effect. Therefore, caution should be exercised when administering dapagliflozin to patients at risk for volume depletion due to co-existing conditions or concomitant medications, such as loop diuretics. These patients should be carefully monitored for volume status, electrolytes, and renal function.

In patients with clinical evidence of upper urinary tract infection (e.g., pyelonephritis) or urosepsis, the Investigator may consider temporarily stopping dapagliflozin treatment until the course of treatment of the infection has been completed and the patient has recovered. That is typically not necessary for lower urinary tract infections,

which should be handled according to clinical routines.

Overdose is defined as the accidental or intentional ingestion of any dose of investigational product that is considered both excessive and medically important. Once the Investigator decides that a particular occurrence is an overdose, it must be reported as an SAE and proper action taken, including necessary healthcare contacts or referrals to emergency unit.

If a patient becomes pregnant during the course of the study investigational product should be discontinued immediately. Pregnancy itself is not regarded as an adverse event unless there is a suspicion that the investigational product under study may have interfered with the effectiveness of a contraceptive medication.

SAE should be reported by the study staff to the investigator/sponsor (A. Rosengren) who completes an SAE document within 24 h of becoming informed of the SAE. The investigator/sponsor evaluates causality.

All SAEs related to Forxiga will also be submitted by the investigator/sponsor to the AstraZeneca Product Safety mailbox: AEMailboxClinicalTrialTCS@astrazeneca.com

SUSAR that are lethal or life-threatening are to be reported to the Medical Product Agency by the sponsor as soon as possible and latest 7 days after the sponsor was being informed using a CIOMS document. Other SUSARs are to be reported as soon as possible and latest within 15 days after the sponsor being informed. The Medical Product Agency will help us to report SUSAR in EUDRA-vigilance.

A Development Safety Update Report will be sent to the Medical Product Agency annually.

9. ETHICAL ASPECTS

9.1 Risks - benefits

The study will be done in accordance with applicable laws and regulation and the principles of GCP and the Helsinki Declaration.

The invasive procedures are the capillary and venous cannulations, which may give local pain. The patients also carry a continuous glucose sensor, which means that a fine needle is placed subcutaneously. Those sensors are usually tolerated well. In cases of erythema or other problems with the sensor, the patient may contact us and may remove the sensor if it causes unacceptable problems.

Both study drugs are approved for T2D at the provided doses. The drugs may be associated with AEs, but relevant precautions have been implemented in the design and planned conduct of the trial in order to minimise the risks and inconveniences of participation in the trial. These precautions include assessment of safety variables, information regarding the correct administration of the drugs, gradual dose adjustment for semaglutide as well as appropriate exclusion and withdrawal criteria.

The most common adverse reactions reported with dapagliflozin are hypoglycaemia (when used with sulphonylurea or insulin which is not relevant here), genital infections, pruritus (generalised) and increased urination. Uncommon adverse reactions are volume depletion and dysuria. There is a risk for diabetic ketoacidosis in patients treated with dapagliflozin. In the DECLARE study, 8574 patients received Forxiga 10 mg and 8569 patients received placebo for a median exposure time of 48 months. Events of DKA were reported in 27 patients in the Forxiga 10 mg group and 12 patients in the placebo group. The events occurred evenly distributed over the study period. Of the 27 patients with DKA events in the FORXIGA group, 22 had concomitant insulin treatment at the time of the event. Precipitating factors for DKA were as expected in a type 2 diabetes mellitus population.

The study participants will receive written and oral information on the signs and symptoms of DKA and are instructed to contact healthcare immediately if DKA is suspected. As a safety measure, subjects with a history of diabetic ketoacidosis will be excluded from this trial. If ketoacidosis is suspected, Forxiga will be discontinued and treatment instituted.

The most commonly reported side effects of semaglutide are gastrointestinal (nausea, diarrhoea and vomiting), typically mild to moderate and of short duration. We will apply a dose escalation regimen to minimize these side effects. Acute pancreatitis has been reported in subjects treated with GLP-1 receptor agonists. As a precaution, subjects with a history of acute or chronic pancreatitis will not be enrolled in the trial. Also, subjects will be informed about the symptoms of acute pancreatitis.

There are several benefits for the participants. They get a better characterization of their disease and general health and we will follow up medically relevant abnormal values. Treatment with study medication is expected to provide clinically relevant improvements in glycaemic control. It is also expected that the subjects will benefit from participation through the contacts with the study site, with close follow-up of

their T2D and a careful medical examination, all of which will most likely result in an intensified management. All subjects in this trial will receive trial products and auxiliary supplies free of charge.

Improved knowledge of which patients benefit most from these clinically approved drugs could open up for more specific and effective treatment in a rather near future, which could benefit both the participants and T2D patients in general.[13-15]

In conclusion, the potential risk to the subjects in this trial is considered low and acceptable in view of the anticipated benefits the study medication will provide to participants.

Risk/benefit assessment in relation to the Covid-19 epidemic

In light of the current Covid-19 epidemic the sponsor should continuously reassess the overall risk and feasibility to conduct the study as the situation develops. This reassessment should be documented. The sponsor should continue safety reporting in adherence to EU and national legal frameworks and should be particularly attentive to reporting of symptoms or signs that could potentially be related to Covid-19.

The Investigator should continuously assess the risk for each participant, based on age, underlying diseases and social situation, and implement measures which prioritize subject safety. Participation for an individual subject may be postponed or inhibited if the Investigator finds it unsafe or otherwise inappropriate to participate. It should be emphasized that subjects have their right to withdraw at any time.

The participants will be instructed to adhere to updated general national recommendations, e.g. not attend the study site in case of infectious symptoms.

It will be ensured that there is an appropriate stock of study material, blood glucose devices and IMP. Any protocol deviations because of Covid-19-related issues should be documented carefully. Actions and substantial changes to the overall assessment should be communicated to the MPA and Ethics committee.

There is a risk that subjects become exposed to Covid-19 by attending study visits, but we do not see that these visits should lead to higher risk of exposure compared to other public places or hospital areas since there is no reason to believe that there is an overrepresentation of SARS-CoV-2 carriers at the study site. The risk is likely lower compared with e.g. waiting rooms at primary care centres.

The IMP is in this case targeting the subjects' diabetic condition (as compared with trials using IMPs testing research questions unrelated to the subjects' underlying disease). Both IMPs are likely to lower the subjects' blood glucose and we have no placebo group. Improved control and management of a chronic condition like diabetes is likely to be beneficial to the subjects during the current circumstances by improving their overall health status and ability to cope with serious infections. The visits will also lead to assessment and better management of other factors of relevance for Covid-19 risk, including elevated blood pressure.

Based on the current risk/benefit evaluation the study is considered feasible to

conduct. The evaluation will however be continuously reassessed.

9.2 Study information and ethical permits

The participants must give written informed consent before included in the study. The original of the consent form will be stored with the Investigator and the participants will receive a copy.

The participants will receive objective and neutral information about the study both in the invitation letter and at the study visits.

The study may start when there are approvals from the Ethical committee, the MPA and the biobank.

Substantial changes should be approved as Amendments by the Ethical committee and/or the MPA.

All correspondence with the Ethical committee and MPA should be saved.

The study will be reported at clinicaltrials.gov.

The Principal Investigator will:

- Ensure each patient is given full and adequate oral and written information about the nature, purpose, possible risk and benefit of the study
- Ensure each patient is notified that they are free to discontinue from the study at any time
- Ensure that each patient is given the opportunity to ask questions and consider the information provided
- Ensure each patient provides signed and dated informed consent before conducting any procedure
- Ensure the original, signed informed consent form is stored in the Investigator's Study File and a copy is given to the patient

Leaving stool samples is optional, and the patient can at any time freely discontinue that component of the study and yet continue with the other parts of the protocol.

9.3 Insurance

The participants are insured through Patientskadelagen and Läkemedelsförsäkringen.

10. REFERENCES

1. Worldwide trends in diabetes since 1980. The Lancet, 2016. 387(10027): p. 1513-1530.
2. Diabetes Prevention Program Research, The Lancet. 374(9702): p. 1677-1686.
3. Davies MJ et al., Diabetologia (2018) 61:2461–2498
4. Ahlqvist et al., Lancet Diabetes and Endocrinology. 2018 March 2
5. Al Jobori H et al., J Clin Endocrinol Metab. 2018 Apr 1;103(4):1402-1407.
6. Bradley C (1994) The Diabetes Treatment Satisfaction Questionnaire: DTSQ. In Bradley C (Ed) (1994) Handbook of Psychology and Diabetes: a guide to psychological measurement in diabetes research and practice.
7. Dalton M et al., European Journal of Clinical Nutrition (2015) 69, 1313–1317
8. H. Wu et al., Nature Medicine 23, 850–858 (2017)
9. Pratley RE et al., Lancet Diabetes Endocrinol. 2018 Apr;6(4):275-286.
10. Wiviott SD et al., N Engl J Med 2019;379:347-57.
11. A. J. Garber, et al., Am. J. Med. 103, 491–497 (1997).
12. R. Arechavaleta, et al., Diabetes Obes Metab 13, 160-168 (2011)
13. McCarthy M. Diabetologia (2017) 60:793–799
14. Pearson E. 2013. Diab Med.31,393-98
15. Fitipaldi H. et al., Diabetes 2018;67:1911–1922
16. Rodbard HW et al., Diabetes Care 2019;42:2272–2281

Summary of Amendments to the study protocol

Amendment 1 dated 21 September 2020

- Ability for trial site to measure HbA1c from capillary instead of venous blood in cases where venous cannulation is difficult to achieve
- Changed criterion from Hba1c at or above 48 mmol/mol to at or above 42 mmol/mol to get a larger span of participants.
- Ability for participants to leave any left-over medication at their local pharmacy rather than at the trial site.

Amendment 2 dated 7 January 2021

- Ability to make rescreening of patients initially screened and included but who were unable to attend their first study visit (the randomization visit) because of Covid-19 restrictions at the hospital (trial site). This meant that patients were rescreened at a later time point (up to six months after initial screening) before attending their first study visit. The delay because of Covid-19 was thus only between initial screening and the randomization to study drugs. Once the participants had been randomized they proceeded according to the stipulated time plan with subsequent visits after 3 and 6 months.

Amendment 3 dated 22 March 2021

- An extension of the time from initial screening to rescreening from six to nine months because of extended Covid-19 restrictions. As stated in Amendment 2, this did not affect the actually study procedures or time frames once the participants had been randomized but only affected the screening period, i.e. participants had to be rescreened to ensure they fulfilled the study criteria before the first study visit.

Study protocol

Semaglutide and dapagliflozin in diabetic patients with different pathophysiology

**Version number:**	**6** (DIAB1)
**Date:**	**22 March2021**
**EUDRA-CT number:**	**2020-000913-33**
**Sponsor and PI**	**Anders Rosengren**

STUDY ADMINISTRATION

Table 1. Study administration

**Function**	**Responsible person**
Sponsor and Principal Investigator	Anders Rosengren, MD PhD Professor<br>Endokrinologiska kliniken Skånes Universitetssjukhus, Region Skåne /<br>Institutionen för neurovetenskap och fysiologi, Box 432, 40530 Göteborg<br>Tel: 070-5316704<br>Email: anders.rosengren@gu.se
Study location	Prövningsenheten, Skåne University hospital, Region Skåne, Sweden

ABBREVIATIONS

AE	Adverse Event
ALP	Alkaline phosphatase
ALAT	Alanine aminotransferase
ANDIS	All New Diabetic In Skåne
AR	Adverse Reaction
ASAT	Aspartate aminotransferase
AUC	Area Under Curve
BMI	Body Mass Index
CRF	Case Report Form
DSUR	Development Safety Update Report
eGFR	Estimated glomerular filtration rate
GCP	Good Clinical Practice
GLP1	glucagon-like peptide 1
HbA1c	Glycosylated haemoglobin A1c
ICH	International Conference on Harmonisation
OGTT	Oral glucose tolerance tes
PRO	Patient-reported outcomes
SAE	Serious Adverse Event
SGLT2	sodium-glucose cotransporter 2
SIDD	Severe Insulin-Deficient Diabetes
SIRD	Severe Insulin-Resistant Diabetes
SUSAR	Suspected Unexpected Serious Adverse Reaction
T2D	Type 2 diabetes
WHO	World Health Organization

SPONSOR AND PRINCIPAL INVESTIGATOR SIGNATURE

It is my responsibility that this protocol contains all essential parts to conduct the study. I am aware of my responsibility that the personnel working with the study are informed about the protocol and other relevant study-related information and are properly trained. The study will be conducted in accordance with the study protocol, the informed consent, ICH GCP, the Declaration of Helsinki and relevant national and international laws and regulations. I am also aware that quality control of the study will be conducted by monitor and potentially also by inspections.

Anders Rosengren	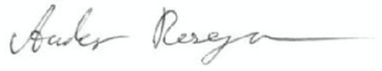	2021-03-22
Sponsor and Principal Investigator	Signature	Date

SYNOPSIS

**Study name**	Semaglutide and dapagliflozin in diabetic patients with different pathophysiology
**Version number**	6
**EudraCT number**	2020-000913-33
**Sponsor and Investigator**	Anders Rosengren
**Background and purpose**	Current anti-diabetic treatment fails to stop the progressive course of the disease. Recent studies have revealed a surprisingly high variability in the diabetic phenotype. We therefore propose that anti-diabetic treatment should ideally target the underlying pathophysiology of each individual patient. We will therefore test whether the effect of two approved anti-diabetic drugs differs between individuals at different ends of the pathophysiological spectrum: 1) patients with poor insulin secretion, here termed SIDD and 2) patients with high insulin resistance, here termed SIRD. The study may open up a new avenue for more precise treatment of diabetic patients that would be of immediate clinical relevance.
**Primary objective**	The primary objective is to study whether the anti-diabetic effect of semaglutide and dapagliflozin, respectively, differs between patients with poor insulin secretion and high insulin resistance, respectively. The primary endpoint will be the intraindividual change of HbA1c in response to semaglutide or dapagliflozin relative to baseline in the two patient groups.
**Secondary objectives**	The secondary endpoints will be the effect of semaglutide and dapagliflozin, respectively, on BMI, waist circumference, PRO, urinary albumin/creatinine index, blood pressure, blood lipids, disposition index, glucose sensitivity, insulin secretory rate, insulin sensitivity index and glucose at 0 and 120 minutes measured from the OGTT in the two patient groups.
**Study design**	This is a randomized open-label parallel arm phase II trial. We will recruit 200 patients with HbA1c ≥42 mmol/mol on metformin monotherapy. Half of them will have poor insulin secretion and half will have high insulin resistance but relatively good insulin secretion. The patients will be randomized (open-label) to receive semaglutide or dapagliflozin for six months in addition to metformin.
**Study population**	Patients with type 2 diabetes
**Number of subjects**	200 (up to 250 in total to cover for replacement recruitment in cases of withdrawal)
**Inclusion criteria**	• Diabetes mellitus based on prior documentation or treatment with anti-hyperglycemic medication or diagnosed according to the WHO criteria (random plasma glucose >11.1 mmol/L or fasting glucose >7.0 mmol/L or HbA1C ≥6.5%) and disease characteristics typical for SIDD or SIRD according to the ANDIS clustering

	• Ongoing metformin therapy with constant dose the last three months<br>• Age 18 years or above<br>• HbA1c ≥42 and <91 mmol/mol<br>• Women who are not postmenopausal and who have not undergone surgical sterilization must have no current pregnancy, which will be assessed by pregnancy test, must take precautions to avoid pregnancy throughout the study and for 4 weeks after intake of the last dose and must be willing to use highly effective birth control methods. Methods that can achieve a failure rate of less than 1% per year when used consistently and correctly are considered as highly effective birth control methods.<br>• Willingness to take injectable and oral medication<br>• Written informed consent
**Exclusion criteria**	• Type 1 diabetes, LADA, MODY, secondary diabetes or history of diabetic ketoacidosis<br>• Anti-diabetic treatment other than metformin within 90 days prior to randomization or changed metformin dose within 90 days prior to randomization<br>• Known acute cardiovascular event, e.g. transient ischemic attack, stroke, acute coronary syndrome, decompensated heart failure, coronary by-pass surgery or other coronary vessel intervention within 90 days prior to screening.<br>• Heart failure NYHA class IV<br>• History of acute or chronic pancreatitis<br>• Known liver cirrhosis<br>• Blood pressure above 170/110 mm Hg<br>• A level of aspartate aminotransferase (ASAT) or alanine aminotransferase (ALAT), ALP or bilirubin of more than three times the upper limit of the normal range<br>• Current chronic daily treatment with an oral steroid at a dose equivalent to oral prednisolone ≥10 mg (e.g., betamethasone ≥1.2 mg, dexamethasone ≥1.5 mg, hydrocortisone ≥40 mg)<br>• Pregnancy or breast-feeding<br>• Known galactose intolerance, total lactase deficiency or glucose-galactose malabsorption.<br>• Estimated glomerular filtration rate <45 ml/min/1,73 m^2 or unstable or rapidly progressing renal disease<br>• Participant unable to understand the study information herself or himself<br>• Involvement in the planning and/or conduct of the study<br>• Participation in other clinical trial which may affect the outcome of the present study<br>• Any condition or treatment that in the judgment of the investigator makes it difficult or unsafe to participate in the study.
**Study medication**	Dapagliflozin (Forxiga) tablets à 10 mg once daily for 6 months

	Semaglutide (Ozempic) s.c. injections 0.25 mg once weekly for four weeks, 0.5 mg once weekly for four weeks, and finally 1.0 mg once weekly throughout the study, total treatment duration 6 months
**Primary variable**	Change from baseline in HbA1c
**Secondary variables**	The secondary variables include BMI, waist circumference, PRO, urinary albumin/creatinine index, blood pressure, blood lipids, disposition index, glucose sensitivity, insulin secretory rate, insulin sensitivity index and glucose at 0 and 120 minutes measured from the OGTT.<br><br>As an exploratory part of the study, we will also analyse proinsulin, glucagon, GLP-1, ASAT, ALAT, ALP and bilirubin, glucose variability, average postprandial blood glucose and time in range as measured by continuous glucose monitoring, blood and urine metabolites and proteins as well as gut bacteria abundance.
**Duration of study**	June 2020 – June 2022

CONTENTS

PAGE
STUDY ADMINISTRATION 2
SPONSOR AND PRINCIPAL INVESTIGATOR SIGNATURE 4
SYNOPSIS 5
CONTENTS 8
1. BACKGROUND AND RATIONALE 10
2. AIMS OF THE STUDY 11
2.1 Primary objective 11
2.2 Secondary objectives 11
2.3 Exploratory objectives 11
2.4 Primary efficacy variable 11
2.5 Secondary and exploratory efficacy variables 12
2.6 Safety variables 12
2.7 Other variables that are not study outcomes 12
3. STUDY DESIGN AND PROCEDURES 13
3.1 Overall study design 13
3.2 Screening visit 15
3.3 Visit 1 (randomization visit) 17
3.4 Between visits 19
3.5 Visit 2 19
3.6 Visit 3 20
3.7 Questionnaire 21
3.8 Blood sampling and biobank 21
3.9 Physical measures 22
3.10 Stool samples 22
3.11 Urine samples 23
3.12 Glucose monitoring 23
3.13 Number of participants 24
3.14 End of trial 24
3.15 Time plan 24
4. SELECTION AND RECRUITMENT OF STUDY PARTICIPANTS 25
4.1 Study criteria 25
4.2 Recruitment 28
4.3 Inclusion 29

5. STUDY COMPOUND 30
5.1 Semaglutide 30
5.2 Dapagliflozin 32
5.3 Rescue medication 33
5.4 Randomization 33
5.5 Packaging, labelling and handling of study medication 34
5.6 Compliance 35
6. STATISTICS 37
6.1 Statistical analyses 37
6.2 Demographics and baseline characteristics 37
6.3 Efficacy analyses 37
6.4 Sample size 38
7. DATA MANAGEMENT 39
7.1 Quality control, data and sample handling 39
7.2 Documentation / Case Report From 40
7.3 Data handling after study 40
7.4 Monitoration 41
8. HANDLING OF ADVERSE EVENTS 42
8.1 Definitions 42
8.2 Reporting and documentation of adverse events 43
9. ETHICAL ASPECTS 46
9.1 Risks - benefits 46
9.2 Study information and ethical permits 48
9.3 Insurance 49
10. REFERENCES 49

1. BACKGROUND AND RATIONALE

An alarming 300 million people have type 2 diabetes (T2D), and the number is expected to exceed half a billion by 2030.[1] Current treatment strategies fail to stop the progressive course of the disease.[2] As a consequence, the disease causes severe complications in the kidneys, eyes and the cardiovascular system, making T2D one of the greatest threats to human health.

Clinical guidelines recommend metformin as initial therapy to all patients but emphasize the need for subsequent personalized treatment with additional drugs.[3] Although this sounds attractive, it is currently implemented on trial-and-error fashion and the concept as such has in fact not been examined systematically and with scientific rigor in a real-world-situation.

We have recently analyzed 9,000 diabetic patients of the ANDIS (All New Diabetics In Skåne) cohort, which highlighted four clusters of T2D patients, each with different characteristics and risk of complications.[4] Two of these clusters are particularly aggressive: one has been coined **SIDD** (**S**evere **I**nsulin-**D**eficient **D**iabetes) and features low age at onset, low BMI and poor insulin secretion; the other, termed **SIRD** (**S**evere **I**nsulin-**R**esistant **D**iabetes), presents at higher age and associates with high BMI and high insulin resistance. Some patients are 'archetypes' of a specific cluster, whilst others have more mixed phenotype.

This sheds new light on the mounting problem of T2D by emphasizing the high variability of the pathophysiology and providing a new tool to distinguish individuals at different ends of the pathophysiological spectrum. Importantly, it leads us to propose that anti-diabetic treatment should ideally target the underlying pathophysiology of each individual patient.

As a starting point we will study whether glucagon-like peptide 1 (GLP1) receptor agonists and inhibitors of the sodium-glucose cotransporter 2 (SGLT2) have different effects on glucose control in patients with SIDD and SIRD characteristics, respectively. These are the only classes of anti-diabetic drugs with proven cardiovascular benefits[3], and it is urgent to get improved knowledge on how to use them as effectively as possible. We will focus on SIDD and SIRD, which enables us to test the feasibility of tailored treatment in a more specific setting (poor insulin secretion and high insulin resistance, respectively). If successful, it may pave the way for combinatorial tailored therapy that is applicable also to patients with more mixed disease phenotype.[13]

Because of their cardiovascular benefits, GLP1 receptor analogues and SGLT2 inhibitors have gained increased use, despite high costs, and they are currently recommended as second-line treatment after metformin.[3] However, it is currently unknown to what extent their effect depends on the underlying pathophysiological characteristics.

Interestingly, our preliminary data from treatment registries coupled with ANDIS show that GLP1-stimulatory drugs, when prescribed in routine care, produce greater glucose-lowering effect in patients who cluster within SIDD compared with SIRD ($p=0.0001$ for HbA1c reduction, χ^2 test; $n=288$ of which 219 are SIDD and 69 SIRD patients).

In light of these observations, we will test the hypothesis that patients with SIDD experience greater therapeutic benefit from the GLP1 receptor agonist semaglutide than SIRD patients. We will also study the effect of the SGLT2 inhibitor dapagliflozin in the two patient groups. Dapagliflozin lowers blood glucose by decreased renal glucose reabsorption and reduced

weight.[3] The compound has also been shown to restore dedifferentiated β-cells and improve insulin secretion, presumably indirectly via reduced hyperglycaemia.[5] It is, however, currently unclear to what extent its effect is influenced by pathophysiological features (too few patients on SGLT2 inhibitors were included in the registry to enable analysis).

2. AIMS OF THE STUDY

2.1 Primary objective

The primary objective is to study whether the anti-diabetic effect of semaglutide and dapagliflozin, respectively, differs between patients with SIDD and SIRD characteristics. The primary endpoint will be the intraindividual change from baseline in HbA1c (ΔHbA1c in mmol/mol) in response to semaglutide or dapagliflozin in SIDD versus SIRD patients, which will be analysed using an ANCOVA model with a term for the exposure and an interaction term for the exposure and subgroup.

2.2 Secondary objectives

The secondary endpoints will be the effect of semaglutide and dapagliflozin, respectively, on BMI, waist circumference, PRO (see section 3.7), urinary albumin/creatinine index, blood pressure, blood lipids, disposition index, glucose sensitivity, insulin secretory rate, insulin sensitivity index and glucose at 0 and 120 minutes measured from the OGTT in SIDD versus SIRD patients using intraindividual comparisons.

2.3 Exploratory objectives

By continuous glucose monitoring for two weeks both before and at 3 months of intervention we will assess glucose variability, average postprandial glucose and time in range.

We will, as an exploratory part of the study, also measure proinsulin, glucagon, GLP-1, liver parameters and blood and urine metabolites and proteins of relevance to cardiovascular and metabolic diseases. The metabolite and protein data will be compared between treatment groups to identify potential markers to predict treatment response and glucose control. Moreover, stool samples will be obtained for subsequent sequencing and analysis of bacterial abundance to study how gut microbiota is affected by treatment and how it predicts treatment response and glucose control. These exploratory analyses aim to open up for potential follow-up studies.

2.4 Primary efficacy variable

The primary variable is change from baseline in HbA1c.

2.5 Secondary and exploratory efficacy variables

The secondary variables include BMI, waist circumference, PRO (see section 3.7), urinary albumin/creatinine index, blood pressure, blood lipids, disposition index, glucose sensitivity, insulin secretory rate, insulin sensitivity index and glucose at 0 and 120 minutes measured from the OGTT.

Exploratory variables are proinsulin, glucagon, GLP-1, ASAT, ALAT, ALP and bilirubin, glucose variability, average postprandial blood glucose and time in range as measured by continuous glucose monitoring, blood and urine metabolites and proteins as well as gut bacteria abundance.

2.6 Safety variables

Blood pressure, pulse rate, plasma sodium, potassium, albumin as well as creatinine and cystatin C (both used to calculate eGFR) will be used as safety variables to monitor volume depletion or changes in kidney function during the study. This will be especially important for patients co-treated with dapagliflozin and diuretics.

Patients who are excluded because of that criterion will be referred to follow-up at the appropriate responsible physician with additional blood pressure measurements and addition of anti-hypertensive medication in line with clinical routines. These patients can be re-screened at a later stage and be included if blood pressure has been appropriately reduced to meet the study criteria. The procedure for follow-up of blood pressure also pertains to patients who are excluded for other reasons than blood pressure but have a blood pressure above 140/90 at screening.

Patients who meet the study criteria and are included but have a blood pressure above 140/90 at any of the visits will be instructed to re-examine their blood pressure either at a primary care unit or at the study site. We will be in close contact with those subjects to ensure that antihypertensive medication is initiated/intensified if blood pressure is repeatedly above 140/90 (defined as two additional measurements on two separate days). Treatment will then be administered using primarily ramipril, followed by amlodipine and metoprolol, but the choice should also be informed by the overall clinical picture, patient preferences and concomitant medication and diseases. If the patient has previously been prescribed anti-hypertensive compound(s) those may be dose-increased based on the overall picture. Initiation or dose elevation of diuretics should be avoided, in particular for patients on dapagliflozin.

2.7 Other variables that are not study outcomes

In addition to the outcome and safety variables we will collect data on age, gender, time since diabetes diagnosis, diabetic complications, metformin dose, Hb (to ensure that changes in Hb do not confound HbA1c measures) and lifestyle data (see Questionnaire description in section 3.7).

3. STUDY DESIGN AND PROCEDURES

3.1 Overall study design

The trial is a clinical phase II study that will be open-label with fixed stratification variables (SIDD and SIRD) to analyze if the response to anti-diabetic drugs differs between patients with distinct pathophysiology, as captured by SIDD and SIRD. The compounds used are semaglutide and dapagliflozin, which will be randomized to patients of each subgroup using a parallel group design.

The clusters (SIDD and SIRD) will be used as a practical tool to distinguish individuals who are at different ends of the pathophysiological spectrum.

We will recruit 200 patients from the ANDIS registry with HbA1c ≥42 mmol/mol on metformin monotherapy (**Fig. 1**). Half of them will have SIDD and half will have SIRD characteristics. The patients will be randomized (open-label) to receive semaglutide or dapagliflozin for six months in addition to metformin.

We will recruit participants on metformin monotherapy with stable dose for the last three months. Metformin dose at inclusion (as prescribed by their regular physician) is maintained throughout the study; we will correct for metformin dose in the analyses. Patients randomized to add semaglutide will receive injection training at the study site and inject 0.25 mg subcutaneously once weekly during the first four weeks, followed by 0.5 mg weekly for the subsequent four weeks and finally 1.0 mg weekly throughout the study. Those randomized to dapagliflozin will receive 10 mg orally once daily in addition to metformin. The participants will attend a screening visit followed by three study visits at 0, 3, 6 months. At the first and last study visit they will undergo an OGTT. HbA1c will be measured at all study visits. Moreover, stool samples will be collected and extra blood and urine will be stored for analyses of metabolites and proteins as exploratory parts of the study. During two periods of the study (before randomization and after three months of treatment) the participants will wear a Libre Pro sensor for continuous glucose monitoring for 2 weeks (**Fig. 1**).

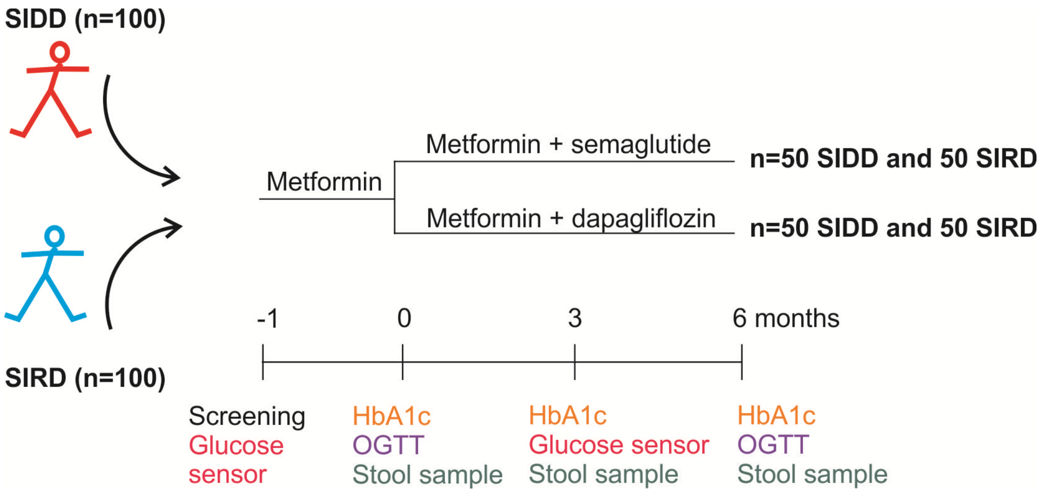

Figure 1. *Schematic of the intervention.*

Table 1. *Summary of study procedures*

	Screening	Visit 1	Telephone contact	Visit 2	Visit 3
**ID check**	X	X		X	X
**Informed consent**	X				
**Patient receives screening ID**	X				
**Patient receives study ID**		X			
**Capillary HbA1c**	X				
**Inclusion & exclusion criteria**	X				
**Medical data recorded**	X				
**Non-fasted venous sampling**	X			X	
**Pregnancy test premenopausal women**	X				
**Hand out glucose sensor**	X			X	
**Hand out tube for stool sample**	X	X		X	
**Hand out tube for urine sample**	X			X	
**Note in medical record**	X	X		X	X
**Note changes in disease/medication**		X	X	X	X
**Collect fecal samples**		X		X	X
**Collect urine sample**		X			X
**Collect glucose sensor**		X			X
**Record AEs**		X	X	X	X
**Height, weight, waist circumference**		X			X

**Blood pressure and pulse rate**	X	X		X	X
**Fasted venous sampling**		X			X
**OGTT**		X			X
**Questionnaire**		X			X
**Randomisation**		X			
**Hand out medication instruction**		X			
**Hand out medication for 3 months**		X		X	
**Hand out diary**		X			
**Follow up compliance**			X	X	X
**Book times for visits 2&3 and tele call**		X			
**Hand out study card to patients**		X			
**Collect diary**					X
**Collect and count remaining medication**					X
**End of study**					X

3.2 Screening visit

Participants who are interested in taking part in the study will attend a screening visit (**Table 1**).

After receiving oral and written information and signing the informed consent the patient will undergo screening process. As a first step the capillary HbA1c will be measured and analysed on-site (appr. 6 min). Only those who have HbA1c ≥42 and <91 mmol/mol proceed to further control of study criteria, and if they are fulfilled we sample venous blood. This procedure is to make screening as effective as possible, as we from previous studies with patients recruited from ANDIS have experienced that HbA1c is the study criteria that is most likely to result in screening failure. Re-screening will be allowed once. A physician will obtain written informed consent and check study criteria while capillary and venous sampling will be done by research nurses or authorized biomedical scientists.

The following will be done at the screening visit after study information and informed consent:

- The participant gets a screening number
- Capillary HbA1c is analysed on-site (appr. 6 min)

If capillary HbA1c ≥42 and <91 mmol/mol we proceed with the following (at the same visit):

- Additional inclusion and exclusion criteria are checked (by physician)
- Relevant medical data are collected, including time since diabetes diagnosis, diabetic complications (retinopathy, neuropathy, nephropathy and other complications) and current medication (name and dose).
- Blood pressure and pulse rate are measured.
- Venous blood samples are drawn for analysis of eGFR, Na, K, ASAT, ALAT, ALP, bilirubin. Total blood volume is estimated to 5 ml.
- A pregnancy test is done for premenopausal women.
- The participant is scheduled for visit 1, which will be within nine months after the screening visit.
- The participant receives a Freestyle Libre glucose sensor pad.
- The participant receives tubes for urine and stool samples and is instructed how to sample at home.

If any necessary information is unknown to the patient at screening, e.g. on relevant medical data, it may be complemented by asking for the patient's permission to obtain the information from the medical journal.

After screening visit:

- If eGFR, ASAT, ALAT, ALP or bilirubin do not meet the study criteria the patient will be contacted and study participation cancelled.
- A letter is sent to the patient's physician managing his/her diabetes with information on the study and that the patient will be randomized to semaglutide or dapagliflozin. The letter will also state that we will be responsible for the diabetes medication while the patient participates in the study and that the antidiabetic medication should not be modified during that time.

Data from the screening visit are recorded on the CRF, except for blood data analyzed at the hospital laboratory, which will be entered into a secure database. The participant receives a screening number (S1001, S1002, etc.). The code list for screening number and personal ID is kept at the study site. Only subjects who have signed the informed consent form should be included on the logs and receive a screening number.

For participants for whom visit 1 cannot be arranged within two months from the screening visit because of e.g. the Covid-19 situation, the following procedure applies:

Visit 1 will be rescheduled to a later time point (within 9 months after screening) and the concerned participants will receive a referral to their local primary care unit to

leave blood samples for analysis of eGFR, Na, K, ASAT, ALAT, ALP, bilirubin (i.e. the safety variables obtained at screening visit) as well as pregnancy test for premenopausal women within four weeks before visit 1. The reason is to ensure that safety variables have not changed since the original screening visit in a way that may lead to exclusion (similar criteria for exclusion apply for eGFR and liver parameters as at the screening visit). Current medication and diseases, including cardiovascular events within 90 days, which may preclude participation, will be checked by the Principal Investigator via phone within four weeks before the visit. This information will be documented in a case-report form. Blood pressure will be measured at visit 1 before they receive any study medication. No new written informed consent is necessary, as that has already been obtained at the screening visit.

Screening failures

Those who attend the screening visit, sign the informed consent but are not eligible to participate will continue to be managed by their regular physician. If necessary, the subject will be referred to relevant healthcare clinic to follow up abnormalities discovered at the screening visit. All reasons for screening failure should be documented, incl. those that occur between screening visit and visit 1. See also 2.6 for follow-up of blood pressure abnormalities.

3.3 Visit 1 (randomization visit)

The participants are recommended not to conduct intense physical activity or drink alcohol 24 h before the visit. They should be fasting since 10pm the previous day. Nicotine users should not have used nicotine the same day.

The following will be done at visit 1:

- Personal ID is checked
- Information on any changes of medication or disease status since last visit is collected
- The participant receives a study ID (different from the screening ID)
- The stool sample brought from home is received and frozen
- The samples with morning urine is collected. One tube is sent for analysis of albumin/creatinine index at the local hospital laboratory and 2x9 ml is frozen for subsequent analyses of urine biomarkers.
- The Libre sensor pad is collected.
- Recording of AEs
- Length, weight, waist circumference, blood pressure and pulse rate are measured
- Venous fasting blood samples are drawn for analysis of HbA1c, Hb, eGFR, Na, K, ASAT, ALAT, ALP, bilirubin, and lipids (HDL, LDL, total cholesterol, triglycerides). Two extra tubes (2x3,5 ml) are stored for subsequent analysis of

metabolites and proteins that are relevant for T2D. Total blood volume is estimated to 15 ml.

- An oral glucose tolerance test (OGTT) is conducted at which patients drink 75 g glucose dissolved in water. Venous blood samples are drawn at 0, 30, 60, 90 and 120 minutes for glucose, insulin and c-peptide. An extra plasma tube (total 3.5 ml) is stored from each time point during the OGTT for subsequent analysis of metabolites and proteins. Total blood volume at each time point is 7 ml, making a total of 35 ml for the OGTT.
- Participants complete a questionnaire on lifestyle habits and PRO (see 3.7).
- Any fresh stool sample is collected.
- The patient is randomized to receive semaglutide or dapagliflozin.
- The patient receives study medication for the coming 3-month period and information about the medication. The patient also receives a diary to mark each time they have taken a dose. Patients randomized to semaglutide will receive injection instructions.
- A note is made in the medical record about the visit.
- The patient receives a tube for stool sampling to be collected before visit 2
- A time is scheduled for telephone contact 2 weeks later, for visit 2 three months later and for visit 3 six months later. The patient also receives a card about the study to show in contacts with healthcare professionals, including the regular physician and nurse managing their diabetes.

In case venous samples cannot be obtained despite repeated attempts, HbA1c may be analysed from a capillary blood sample using an Alere Afinion AS100 reader and capillary blood glucose before and during the OGTT by a Hemocue glucose reader (see also 3.8). The secondary variables blood lipids, disposition index, glucose sensitivity, insulin secretory rate and insulin sensitivity index will then be missing for the participant. These missing data will not be imputed in subsequent data analysis of secondary variables.

Data from the visit are recorded on the CRF, except for blood data analyzed at the hospital laboratory, which will be entered into a secure database.

Subsequent visits should ideally be scheduled on the same weekday as visit 1. If that is not possible, e.g. because of public holidays or unavailability of the participant to come on an assigned day, the subsequent visit will be scheduled as soon as possible three months after the previous visit. Reasonable measures should be taken to schedule the visits at three month intervals but a delay of up to three weeks is acceptable to accommodate for travels, sudden illness etc. The participant will be reminded by email or mobile text message before next visit. Participants should initiate study drug treatment latest within 4 weeks after Visit 1 and visits are arranged such that total time from initiation of study drug to visit 3 is 6 months.

3.4 Between visits

The participant continues taking all their regular treatment during the study. They are instructed not to change their metformin dose or general lifestyle habits (e.g. overall dietary pattern) during the study and contact us before any such changes are done.

The study team will contact the participants by phone appr. 2 weeks after the initiation of the treatment to record any AEs and to check compliance. Adverse events are noted, and appropriate follow-up is initiated. Reasonable efforts should be undertaken to get in contact with the participants by calling back if no reply and/or sending emails. Participants could contact us at their own initiative in case they have any suspected side effects of the treatment or experience problems with the semaglutide injections. Patients may also be contacted by phone at additional time points to follow-up on adverse events, compliance or injection problems.

Patients who at Visit 1 have total cholesterol ≥7.0 mM, LDL cholesterol ≥5.0 mM or triglycerides ≥5.6 mM will be followed up with an additional test of fasting lipid levels. If any value is still above these limits appropriate medication will be initiated/dose-increased. Atorvastatin should be the primary drug of choice but the overall clinical picture, patient preferences and concomitant medication and diseases should also be taken into account. See also point 2.6 for follow-up of blood pressure abnormalities.

3.5 Visit 2

The participants do not need to be fasting at visit 2 (visits can be done in the afternoon).

The following will be done at visit 2:

- ID is checked
- Information on any changes of medication or disease status since last visit is collected
- Stool samples are received and frozen
- Recording of AEs
- Blood pressure and pulse rate are measured
- Venous blood samples are drawn for analysis of HbA1c, eGFR, albumin, Na, K. Total blood volume is estimated to 8 ml.
- Any fresh stool sample is collected.
- The participant receives a Freestyle Libre glucose sensor pad to wear for two weeks.
- Patient brings remaining study medication and diary, which are assessed. Any discrepancies are discussed to reinforce compliance, and the participant receives medication for the next 3-month period.
- The participant receives tubes for stool and urine to be sampled before visit 3

- The time scheduled for visit 3 three months later is checked.
- A note is made in the medical record about the visit.

Data from the visit are recorded on the CRF, except for blood data analyzed at the hospital laboratory, which will be entered into a secure database.

3.6 Visit 3

The participants are recommended not to conduct intense physical activity or drink alcohol 24 h before the visit. They should be fasting since 10pm the day before. Nicotine users should not have used nicotine the same day. The last dapagliflozin tablet should be taken the evening before Visit 3 and the last semaglutide injection 3 days before Visit 3 (see also 5.1 and 5.2).

The following procedures will be done at visit 3:

- ID is checked
- Information on any changes of medication since last visit is collected
- Stool samples are received and frozen
- The samples with morning urine is collected. One tube is sent for analysis of albumin/creatinine index at the local hospital laboratory and 2x9 ml is frozen for subsequent analyses of urine biomarkers.
- The Libre sensor pad is collected
- The diary is returned
- Remaining study medication is returned, counted and stored temporarily before destruction
- Recording of AEs
- Weight, waist circumference, blood pressure and pulse rate are measured
- Venous fasting blood samples are drawn for analysis of HbA1c, Hb, eGFR, Na, K, ASAT, ALAT, ALP, bilirubin, and lipids (HDL, LDL, total cholesterol, triglycerides). Two extra tubes (2x3,5 ml) are stored for subsequent analysis of metabolites and proteins that are relevant for T2D. Total blood volume is estimated to 15 ml.
- An oral glucose tolerance test (OGTT) is conducted at which patients drink 75 g glucose dissolved in water. Venous blood samples are drawn at 0, 30, 60, 90 and 120 minutes for glucose, insulin and c-peptide. An extra plasma tube (total 3.5 ml) is stored from each time point during the OGTT for subsequent analysis of metabolites and proteins. Total blood volume at each time point is 7 ml, making a total of 35 mL for the OGTT.
- Participants complete a questionnaire on lifestyle habits and PRO (see 3.7).
- A note is made in the medical record about the visit.

- End of study for the participant.

In case venous samples cannot be obtained despite repeated attempts, HbA1c may be analysed from a capillary blood sample using an Alere Afinion AS100 reader and capillary blood glucose before and during the OGTT by a Hemocue glucose reader (see also 3.8). The secondary variables blood lipids, disposition index, glucose sensitivity, insulin secretory rate and insulin sensitivity index will then be missing for the participant. These missing data will not be imputed in subsequent data analysis of secondary variables.

Data from the visit are recorded on the CRF, except for blood data analyzed at the hospital laboratory, which will be entered into a secure database.

3.7 Questionnaire

The participants are instructed not to make any major lifestyle changes during the study (e.g. major changes of dietary pattern, initiation of weight reduction programmes etc.). We will assess lifestyle habits by a questionnaire, which will assess the following:

- Physical activity using the short IPAQ (international physical activity questionnaire), which is a commonly used scale to assess physical activity.
- Dietary habits using items that have been validated in Stockholm health questionnaire 2010 (Stockholms folkhälsoenkät 2010).
- Tobacco and alcohol, by validated items previously used by the national Health questionnaires in Sweden (Folkhälsoinstitutets nationella folkhälsoenkäter).

We will also assess patient-reported outcomes by

- Diabetes Treatment Satisfaction Questionnaire[6]
- Control of Eating Questionnaire (CoEQ).[7] It comprises 21 items designed to assess the intensity and type of food cravings, as well as subjective sensations of appetite and mood. We will use 19 of its items to assess the subscales of craving control, positive mood, craving for savoury and craving for sweet. This is of relevance because both dapagliflozin and semaglutide may affect appetite.[16]

The questionnaires take appr. 15-20 minutes to complete.

3.8 Blood sampling and biobank

The Investigator should make an assessment of the available results with regard to clinically significant abnormalities. The laboratory reports should be signed and

retained as source data for laboratory variables.

Venous HbA1c, Hb, creatinine, cystatin C, Na, K, albumin, ASAT, ALAT, ALP, bilirubin, lipids (HDL, LDL, total cholesterol, triglycerides), insulin as well as urine albumin/creatinine index are analysed at the accredited hospital laboratory at Skåne University hospital. Venous blood glucose is analysed directly via a Hemocue glucose reader. Capillary HbA1c at screening visit (and in rare cases at Visit 1 and 3 – see 3.3 and 3.6) is analysed by an Alere Afinion AS100 reader according to the manufacturer's instructions. All these samples are analysed within the same day as the visit and destroyed immediately. The extra blood tubes that are obtained for later analyses will be handled according to the Biobank law and regulations. The samples will be pseudonymized using a study ID. The code list for study ID/personal ID will be stored securely and separately at the study site and after the study by the sponsor to prevent unauthorized persons to access them.

If a patient withdraws consent to the use of donated biological samples, the samples will be destroyed, and the action documented. If samples are already analysed, the sponsor is not obligated to destroy the results of this research.

3.9 Physical measures

Blood pressure and pulse rate

Blood pressure and pulse rate will be measured using a standardized cuff adapted to the size of the patient's arm after the patient has been sitting and resting for least 5 minutes. The subject should not talk during the measurement.

Body weight, height and waist circumference

The patient's height will be recorded at Visit 1 in centimeters, with no shoes.

The patient's body weight will be recorded in kilograms, to 1 decimal place, with light clothing and no shoes and recorded at Visits 1 and 3.

The waist circumference will be measured in cm as the minimal abdominal circumferences located midway between the lower rib margin and the iliac crest. The subject should be standing with arms down their side and feet together. The tape should touch the skin but not compress soft tissue and twists in the tape should be avoided. The subject should be asked to breathe normally and the measurement should be taken when the subject is breathing out gently.

3.10 Stool samples

Stool sampling is optional. The patient will receive a tube and written instructions on how to sample. Briefly, they are instructed to collect a stool sample within 24 h before their study visit, store the sample at room temperature and bring it to the study site. They may also bring it to the study site up to two days after their visit as long as it is less than 24 h since sampling. The sample will be frozen at the study site. The date and time of receiving the sample and the time of freezing should be entered into a log.

They are also asked to collect a second stool sample at the study visit, if possible, for immediate freezing. This is to enable analysis of a subset of samples that have been frozen immediately to study the difference in quality between samples stored at up to 24 h at room temperature and those frozen immediately. That will be important for evaluating the quality of microbiota data from this and subsequent studies.

After the study the stool samples will be sequenced to analyzed gut bacterial abundance.[8] Also gut bacterial metabolites will be analysed. The aims of these analyses are to

- study any differences in gut bacterial composition that distinguish SIDD and SIRD patients
- study the effect of the drugs on gut bacterial composition
- predict drug responders and non-responders
- predict disease deterioration and complications (see 7.3).

3.11 Urine samples

The patient receives tubes for urine sampling to sample first morning urine and bring to visit 1 and 3. From that, a 10 ml aliquot is sent for analysis of albumin/creatinine index at the local hospital laboratory and 2x9 ml is frozen for subsequent analyses of urine biomarkers.

3.12 Glucose monitoring

A Freestyle Libre Pro, provided by Abbott, will be used to monitor glucose continuously in study participants. The study personnel will put a pad on the participant's arm (this has to be done by healthcare personnel for the Pro sensor as opposed to the standard Libre sensor) and the participant wears the pad during two weeks. There is no connected reader (as opposed to the standard Libre sensor), which is an advantage in this case because the patients will not receive any feedback on their glucose values, which could otherwise lead to dietary adjustments and recording biases. Included participants wear the sensor for two weeks from the screening visit, then take it off themselves and return it to the study center, either by a posted envelope or at their subsequent visit. They do not wear any sensor between Visit 1 and 2. At Visit 2, when they have been on study medication for three months, they receive a new sensor, wear it for two weeks and return it to the study center.

Each sensor has a serial number that will be connected with a study ID and recorded in a separate document at the study site. The sensor can store the equivalent of two weeks' recordings and holds the data indefinitely. Upon return the sensor data is transferred from the pad to a Freestyle Libre Pro Reader, which is available at the study center, and is then transferred (by a USB cable) to a stationary computer. Glucose variability, time in range and postprandial glucose concentrations will after the study be analyzed and compared before and after treatment for each individual. Study ID will be used in these analyses.

If a study participant is already wearing a standard Freestyle Libre sensor (e.g. prescribed by healthcare) he/she will be allowed to wear it during the study but will still receive a Freestyle Libre Pro sensor by us to collect study data.

If the Libre Pro sensor pad comes to loose earlier than two weeks, the patient can either return the pad (although it will contain data from a shorter time period) or visit us to receive a replacement pad. This will be determined case by case by the study personnel depending on the timing of subsequent visits. If a patient experiences skin reactions to the sensor he/she will be instructed to contact us. The pad may in those cases be removed at the discretion of the Investigator.

3.13 Number of participants

200 participants will participate in the study; 100 with SIDD and 100 with SIRD. They will be randomized to semaglutide or dapagliflozin at a 1:1 ratio. Participants with both SIDD and SIRD will be recruited simultaneously. We aim to recruit at least 50% of participants having HbA1c ≥48.

In case of withdrawals or exclusions during the study we will recruit new participants to get the required number of participants for the full analysis set (see also 6.4).

3.14 End of trial

End of trial is defined by last subject, last visit. For this type of trial we have not identified any stopping criteria for the entire study as it is using approved drugs. Should the study anyway be terminated prematurely, e.g. in case of force majeure, the PI will immediately inform the participants and ensure proper follow-up. Relevant authorities will also be notified, latest within 15 days. The decision on termination is taken by sponsor.

Latest 90 days after end of trial, the Medical Product Agency will be informed by a Declaration of End of Trial Notification.

3.15 Time plan

First recruitment activities: June 2020

First subject in: August 2020

Last subject in: December 2020

Last subject last visit: June 2021

Final report: latest June 2022

4. SELECTION AND RECRUITMENT OF STUDY PARTICIPANTS

4.1 Study criteria

Inclusion criteria

- Diabetes mellitus based on prior documentation or treatment with anti-hyperglycemic medication or diagnosed according to the WHO criteria(random plasma glucose >11.1 mmol/L or fasting glucose >7.0 mmol/L or HbA1C ≥6.5%) and disease characteristics typical for SIDD or SIRD according to the ANDIS clustering
- Ongoing metformin therapy with constant dose the last 90 days
- Age 18 years or above
- HbA1c ≥42 and <91 mmol/mol
- Women who are not postmenopausal and who have not undergone surgical sterilization must have no current pregnancy, which will be assessed by pregnancy test, must take precautions to avoid pregnancy throughout the study and for 4 weeks after intake of the last dose and must be willing to use highly effective birth control methods. Methods that can achieve a failure rate of less than 1% per year when used consistently and correctly are considered as highly effective birth control methods. Such methods include:
    - combined (estrogen and progestogen containing) hormonal contraception associated with inhibition of ovulation: oral, intravaginal or transdermal
    - progestogen-only hormonal contraception associated with inhibition of ovulation: oral, injectable or implantable

- intrauterine device
- intrauterine hormone-releasing system
- bilateral tubal occlusion
- vasectomised partner
- sexual abstinence

- Willingness to take injectable and oral medication
- Written informed consent

Exclusion criteria

- Type 1 diabetes, LADA, MODY, secondary diabetes or history of diabetic ketoacidosis
- Anti-diabetic treatment other than metformin within 90 days prior to inclusion
- Known acute cardiovascular event, e.g. transient ischemic attack, stroke, acute coronary syndrome, decompensated heart failure, coronary by-pass surgery or other coronary vessel intervention within 90 days prior to inclusion.
- Heart failure NYHA class IV
- History of acute or chronic pancreatitis
- Liver cirrhosis
- Blood pressure above 170/110 mm Hg
- Current chronic daily treatment with an oral steroid at a dose equivalent to oral prednisolone ≥10 mg (e.g., betamethasone ≥1.2 mg, dexamethasone ≥1.5 mg, hydrocortisone ≥40 mg)
- Pregnancy or breast-feeding
- Known galactose intolerance, total lactase deficiency or glucose-galactose malabsorption.
- Participant unable to understand the study information herself or himself, (information will only be available in Swedish and we have no interpreter resources)
- Involvement in the planning and/or conduct of the study
- Participation in other clinical trial which may affect the outcome of the present study
- Any condition or treatment that in the judgment of the investigator makes it difficult or unsafe to participate in the study.

Patients will be excluded and should not be randomized if the following are observed from laboratory tests analysed after screening visit:

- Estimated glomerular filtration rate <45 ml/min/1,73 m^2 or unstable or rapidly progressing renal disease

- A level of aspartate aminotransferase (ASAT) or alanine aminotransferase (ALAT), ALP or bilirubin of more than three times the upper limit of the normal range

Criteria for withdrawal

- The participant can withdraw from the study at any time without further motivation and without any consequences for his/her future treatment.
- The participant will be withdrawn should he/she develop any condition or begin taking any compound (apart from the study medication) that is part of the exclusion criteria. If blood pressure at any visit is above 170/110 the measurement will be repeated at least twice at two different days before withdrawal to ensure that it is not a chance finding.
- A participant can be withdrawn if he/she does not adhere to the procedures as specified in the protocol.
- If the participant experiences serious adverse events and there are reasons to think that the event is related to the study, he/she will be immediately withdrawn and prompt action will be taken to assist the participant as necessary.
- Acute renal insufficiency or worsened chronic renal insufficiency (a decrease of eGFR of 15 mL/minute/1.73m^2 or greater compared with baseline) as verified by repeat eGFR values. The re-test should be scheduled within 4 days, whenever possible.

  If an unexpected, acute decline in kidney function is observed, the patient should be promptly evaluated. Urinary tract infection and urinary obstruction should be considered. Several drugs may cause a decline in kidney function, especially non-steroidal anti-inflammatory drugs (NSAID) and certain antibiotics such as trimethoprim. If any drug is suspected of causing or contributing to worsening kidney function, their use should be re-considered.

A patient who is withdrawn and have taken the study medication for at least 3 months with 80-120% compliance will be asked to attend a final visit, which will then follow the procedures outlined for Visit 3. We will inform them that any such visit is voluntary.

A patient who decides and/or is recommended by treating physician to permanently discontinue study medication, will be asked about the reason(s) and the presence of any AEs. AEs will be followed up according to clinical routine also for patients who are withdrawn (see also section 8.2). The primary reason for discontinuation of study medication must be specified in the CRF unless the patient declines to motivate the reason.

Female subjects must be instructed to notify the investigator immediately if they become pregnant during the trial.

All participants who are withdrawn will continue their regular medication and their ordinary physician will be notified as appropriate.

4.2 Recruitment

Patients with T2D will be recruited from ANDIS (Alla Nya Diabetiker I Skåne / Ethical approval nr. 584/2006), which registers diagnosed diabetic patients in Region Skåne (southern Sweden) since 2008 (http://andis.ludc.med.lu.se/).

Procedures in ANDIS

When a patient in Region Skåne is diagnosed with diabetes they are asked by their regular physician to be included in the ANDIS registry. Patients who want to be included sign an informed consent, and a blood sample is drawn for analysis of fasting glucose, C-peptide (measure of insulin secretion) and GAD antibodies (sign of autoimmune diabetes). These data as well as data on age, BMI, HbA1c, fasting glucose and basic clinical characteristics are stored within Region Skåne. Patients who are registered in ANDIS are managed by their regular physician. There are no follow-up visits in ANDIS, but on the ANDIS informed consent form patients are asked whether they would like to be contacted for recruitment to other studies of relevance for diabetes. ANDIS is led by a steering group with representatives from academia and healthcare. Prof Leif Groop is scientific PI and founder of ANDIS. Anders Rosengren is clinically responsible for ANDIS since 2014.

Cluster analysis in ANDIS

In a recent collaboration, we have obtained data that suggest a need for a diagnostic refinement to better reflect the variable patient characteristics.[4] We performed a data-driven k-means cluster analysis of 9,000 diabetes patients in the ANDIS registry based on six variables measured at diagnosis: GAD antibodies (antibodies to pancreatic beta-cells), age, BMI, HbA1c, HOMA2-B (reflecting insulin secretion and calculated from glucose and c-peptide concentrations) and HOMA2-IR (reflecting insulin resistance and calculated from glucose and insulin concentrations). Four clusters of T2D patients were highlighted, each with different characteristics and risk of complications (a separate group corresponding to type 1 diabetes with elevated GAD antibodies and poor insulin secretion was also identified). The findings were replicated in three additional cohorts.[4]

Recruitment from ANDIS in this study

Here we will recruit patients with T2D who cluster within SIDD or SIRD in the ANDIS registry and who have accepted to be contacted with information on diabetes-related studies. We have previously recruited patients from ANDIS e.g to the studies "Randomized study with yohimbine in type 2 diabetes patients with or without a genetic risk variant" (EUDRA-CT 2010-018604-85, Ethical permit nr. 2011/587) and Effect of broccoli in type 2 diabetes (Ethical permit nr. 2015/395).

We will reach out to patients via 1) letters and 2) advertisements. For the letters, the PI or by him assigned person receives a list from the ANDIS database manager (Jasmina Kravic) with patients clustering within SIDD or SIRD. The list of potentially eligible

patients is generated using coded data in the ANDIS database. The list is then uncoded (by Jasmina Kravic) in order to obtain the patients' personal ID so that we can reach them. This list is stored at a local server behind the hospital firewall. Next, letters are sent to these patients with the study information. If we do not get any response within one month a reminder letter may be sent out. We will also recruit via advertisements (see advertisement text as appendix). Patients who become interested in the study, either via letters or advertisements, go to a dedicated website, which has a simple booking interface. The website has the necessary IT security and the participants will not be able to see which other persons have booked a time (they will only see available and non-available slots). They enter their name, personal number, telephone number and email address and receive a confirmation email with instructions for the subsequent procedure. They can also contact us by email or phone if they have any questions.

The ANDIS registry covers a large number of diabetic patients in Skåne (more than 20,000 patients since appr. 100 primary care centres and 9 hospitals in Skåne are involved in ANDIS) but there are also patients with diabetes in the region who are not included in the ANDIS registry (exact numbers are difficult to estimate but based on prevalence data there should be appr. 40,000 T2D patients in Skåne). These patients may still see the advertisement and become interested in the study. In that case they have to be registered in ANDIS in order for us to analyse their cluster assignment (to know if they have SIDD or SIRD or belong to any of the other three clusters that are not assessed in this study). For mathematical reasons, the patients need to be part of ANDIS as the cluster analysis is based on how they relate to other patients in terms of GAD antibodies, age, BMI, HbA1c, HOMA2-B and HOMA2-IR, using the ANDIS clustering algorithm as in ref. 4.

We will therefore check whether patients who sign up for a screening visit are already included in ANDIS and if so, whether they have SIDD or SIRD. Patients who are not in the ANDIS registry will be contacted and will be asked to be included in ANDIS. These patients will receive specific study information in which we explain that participation in the ANDIS registry is voluntary but a prerequisite to take part in this study. If they want to take part, they will book a time to a nurse at the Clinical Research Center, Skåne university hospital Malmö, for a visit according to the standard procedures in ANDIS. Once they have given informed consent, blood will be drawn and ANDIS data obtained for analysis of cluster assignment (see Procedures in ANDIS above). If they cluster within SIDD or SIRD they will be given the opportunity to attend a screening visit for the present study following the same procedure as all other patients. Patients in other clusters will not be able to take part in this study but will remain in ANDIS (unless they withdraw their ANDIS consent). We inform them beforehand that inclusion in ANDIS does not automatically mean that they can be enrolled in the present study as that depends on their cluster assignment and fulfilment of other study criteria. They are also informed that they can withdraw their consent to ANDIS or the present study at any point.

4.3 Inclusion

If the participants have given informed consent and fulfil all study criteria they will be

formally included at visit 1. Included participants receive a study ID, which is different from the screening number. The code list for study ID and personal ID is kept with the Investigator at Skåne University hospital. Only coded data, based on the study ID, will be used for analyses.

5. STUDY COMPOUND

The patients will remain on the metformin dose they had at inclusion and are instructed not to change this dose during the study. They continue with their current medication throughout the study and all concurrent medication is entered on the CRF and any changes are documented. All background medication is considered to be non-investigational medicinal product. We have no placebo treatment in this study. The study medications are semaglutide and dapagliflozin, as described in more detailed below.

5.1 Semaglutide

Semaglutide (Ozempic, manufactured by NovoNordisk) is a human GLP-1 receptor agonist for once weekly subcutaneous administration.[3,9]

The start dose is 0.25 mg once weekly during the first four weeks, then 0.5 mg weekly during four weeks and finally 1.0 mg weekly throughout the study. Dose reduction to 0.5 mg will be allowed if the participant experiences unacceptable side effects at 1.0 mg. In those cases we will adjust for dose reduction in the analyses. If a dose is missed it should be taken as soon as possible or at least within 5 days after the missed dose after which the patients continue on their regular weekly schedule. If more than five days have elapsed the dose will be counted as a missed dose and the regular schedule continues.

The participant is instructed to adjust the administration schedule such that the last semaglutide dose is taken 3 days before Visit 3. This is to standardize administration in relation to the final OGTT.

It is injected subcutaneously in the abdomen, thigh or upper arm at any time of day, irrespective of meals. It should not be injected intravenously or intramuscularly.

Semaglutide preparations (both not in-use and in-use) must not be exposed to excessive heat or direct sunlight. Semaglutide preparations which have been frozen must not be used. It must not be used if it does not appear clear and colourless.

Storage conditions for semaglutide are as follows:

Not in-use:

- Store in a refrigerator (2°C to 8°C).
- Do not freeze.
- Protect from light.

In-use:

- Store below 30°C or in a refrigerator (2°C to 8°C).
- Use within 1 month.
- Do not freeze.
- Protect from light.

These instructions will be given to the patients.

The study site must ensure the availability of proper storage conditions and record and evaluate the temperature. The temperatures during storage should be monitored by a calibrated, stationary and continuously recording system. A temperature log must be kept to document storage within the right temperature interval and storage facilities should be checked frequently. Fifteen minutes outside the indicated range is negligible and allowed, and should not be recorded as a deviation.

Returned trial products (unused, partly used or used including empty packaging material) must be stored separately from non-allocated trial products before being sent for destruction.

No dose adjustment is necessary with regard to age, kidney or liver function.

Acute pancreatitis has been observed after the use of GLP1 receptor agonists.[3] The patients will be informed of the characteristic symptoms of acute pancreatitis and medication should be temporarily stopped if it is suspected and permanently stopped if it is verified. Patients with previous pancreatitis should not be included in the study (see 4.1 Exclusion criteria).

The most commonly reported side effects are gastrointestinal (nausea, diarrhoea and vomiting), typically mild to moderate and of short duration. In previous studies a total of 6.1% and 8.7% of patients interrupted their medication on 0.5 and 1.0 mg semaglutide, respectively, because of side effects, as compared with 1.5% in the placebo groups. The risk for hypoglycaemia is small in this study since the patients do not have concomitant insulin or sulfonylurea medication (the frequency of hypoglycaemia in previous studies was 0.001 cases per patient year). Erythema at the site of injection has been reported among 0.5% of the patients, typically of mild nature.

In subjects treated with GLP-1 receptor agonists AEs such as nausea, vomiting and diarrhoea may lead to significant dehydration and secondary acute renal impairment. Subjects with gastrointestinal AEs are recommended to drink plenty of fluids to avoid

volume depletion. As a safety measure, markers of kidney function will be monitored throughout the trial.

Semaglutide has not been shown to affect the exposure or pharmacodynamics of warfarin. However, it is still recommended to assess PK-INR after initiation of semaglutide in patients on warfarin treatment. This will be handled by the investigator case-by-case.

5.2 Dapagliflozin

Dapagliflozin (Forxiga) is manufactured by AstraZeneca and will be provided as tablets of 10 mg.[3,10] The dose is the recommended clinical dose for type 2 diabetes patients. No dose adjustment is necessary with regard to kidney function, but the effect on glucose control is decreasing with reduced glomerular filtration rate (GFR). Estimated GFR (eGFR) below 45 ml/min/1,73 m^2 will therefore be an exclusion criterion and rapidly progressing renal insufficiency will be reason for withdrawal (see 4.1). Dose reduction is necessary if liver function is severely deteriorated, and severe liver disease (known cirrhosis) is therefore an exclusion criterion. No dose adjustment is recommended based on age. The patients who are randomized to dapagliflozin will take one tablet daily for 6 months. It can be taken at any time of day with or without food. The patients are however instructed to take dapagliflozin in the evening three days prior to Visit 3. This is to standardize administration in relation to the final OGTT.

The tablets are to be stored at room temperature.

If the patient suffers from dehydration, e.g. because of a gastrointestinal infection, dapagliflozin medication should be temporarily paused until the volume loss has been corrected. The patients are recommended to contact us should this occur, and proper action is taken by the PI case by case. Temporary pause of treatment will also be considered if the patient develops pyelonephritis or urosepsis.

Rare cases of Fournier's gangrene have been reported and the patients will be instructed to contact healthcare in case they develop edema or pain in the perineal region.

Dapagliflozin is not recommended during pregnancy, especially during the second and third trimester. We screen for pregnancy in premenopausal participants and recommend immediate contact with us in case of pregnancy.

These instructions are given in both oral and written form at the randomization visit.

Studies have not shown any increased frequency of hypoglycemic events when dapagliflozin is taken in combination with metformin (unless combined with sulfonylurea or insulin).[10] Urogenital infections are common side effects (4.7% compared with 3.5% with placebo in previous studies) and will be managed in accordance with clinical routines and the judgement of the Investigator. Usually, dapagliflozin treatment can continue in those cases.

The tablets contain lactose, and known galactose intolerance, total lactase deficiency or glucose-galactose malabsorption are therefore exclusion criteria.

If the patient misses a dose he/she should take only one tablet as usual the next day.

Patients mark their daily intake in a diary, which is to be returned at the final visit.

5.3 Rescue medication

If the participant develops HbA1c ≥91 mmol/mol or diabetic ketoacidosis (see below for definition) rescue medication will be initiated in the form of injectable insulin. The participant will be asked to attend a study visit as soon as possible, which will then follow the procedures outlined for Visit 3. These patients will be included in the full analysis set but not in the per protocol analyses. These participants will then end the study and will be referred to healthcare for continued follow-up.

Diabetic Ketoacidosis (DKA) definition

A diagnosis of Diabetic Ketoacidosis should only be made in a clinical setting consistent with DKA (based on patient history, symptoms, and physical exam) and in the absence of more likely alternative diagnoses and causes of acidosis (such as lactic acidosis). The following biochemical data should support diagnosis:

• Ketonaemia ≥3.0 mmol/L and/or significant ketonuria (more than 2+ on standard urine sticks) and at least one of the following criteria suggesting high anion gap metabolic acidosis:

a) Arterial or Venous pH ≤7.3

b) Serum bicarbonate ≤18 mEq/L

c) Anion gap [Na – (Cl + HCO_3)] >10

5.4 Randomization

The randomization will be organized via an independent statistician using a computer-based block randomization algorithm with balanced blocks. Randomization will be stratified for SIDD and SIRD, respectively, so that we get an approximate distribution of 1:1 semaglutide:dapagliflozin in both SIDD and SIRD.

The randomization list will be numbered 1-200 for SIDD patients and 201-400 for SIRD patients with each number corresponding to either semaglutide or dapagliflozin. Sealed envelopes will be prepared for each participant with study ID printed on the outside. Each envelope contains a card informing about the assigned study medication. The envelope corresponding to the patient's study ID will be opened at visit 1 by the site staff (information is blinded to study personnel, Investigator and participant before randomization). The patient receives corresponding study medication.

After visit 1 the assignment is open-label. The study medication taken by each patient will be entered in the medical journal and in a letter to be sent to the physician normally managing the patient's diabetes.

The full randomization list (in a sealed envelope) is kept at the study site during the entire study until final analyses. No emergency envelopes are needed as the study is open-label and the assigned study medication is entered in the medical record.

If a participant is withdrawn during the study, that study ID will not be reused and the participant is not allowed to take part again.

5.5 Packaging, labelling and handling of study medication

Dapagliflozin (Forxiga) will be provided by AstraZeneca and semaglutide (Ozempic) will be provided by NovoNordisk using similar manufacturing, quality controls and packaging as for routine clinical use. The study medication will be sent to Tamro AB in Sweden, where the packages will be labelled with study-specific labels (in addition to the standard labelling of the drug package from the manufacturer).

Study labels will be prepared by Tamro AB in accordance with Good Manufacturing Practice (GMP) and local regulatory guidelines. The labels will fulfil GMP Annex 13 requirements for labelling. The labels will contain the following information:

> Trial medication, study name and EudraCT number, name and contact details of Investigator and storage conditions.

Tamro AB will send study medication to the study site, where it will be stored under appropriate conditions. As participants become randomized they receive study medication in a quantity corresponding to the coming 3-month period. The study personnel manually write (with a ballpen) the participant’s study ID on the packages that are handed out as well as the order of packages to be used (of relevance only for the dose escalation of semaglutide). The participant also receives written information about the study medication and a diary. In the diary, the study personnel write the date of the first dose to be taken and the participant then makes a note every time they take a dose. Participants randomized to semaglutide will receive injection instructions at visit 1.

<u>The participants randomized to semaglutide will receive the following:</u>

At visit 1:

> 1x Ozempic 0,25 mg, 1 x 4 doses
>
> 1x Ozempic 0,5 mg, 1 x 4 doses
>
> 1x Ozempic 1,0 mg, 1 x 4 doses

At visit 2:

> 3x Ozempic 1,0 mg, 1 x 4 doses

Participants who need additional medication, because of e.g. failure or misuse of injection pens or delayed visits, may at the discretion of the Investigator receive additional medication. Dose reduction to 0.5 mg will be allowed if the participant experiences unacceptable side effects at 1.0 mg.

The participants randomized to dapagliflozin will receive the following:

At visit 1:

1x Forxiga 1 x 98 tablets à 10 mg

At visit 2:

1x Forxiga 1 x 98 tablets à 10 mg

Participants who need additional medication, because of e.g. delayed visits may at the discretion of the Investigator receive additional medication. That will be provided as packages containing Forxiga 1 x 28 tablets à 10 mg.

5.6 Compliance

At each visit and telephone contact the investigator will remind the subject to adhere to trial procedures described in the protocol.

Subject compliance will be assessed by monitoring of drug accountability. Prior to visit 2 and 3 the subject will be asked to return all used, partly used and unused trial products. The investigator must assess the amount of trial products returned compared to what was dispensed at the last dispensing visit and if a subject is discovered to be non-compliant, the investigator must inform the subject of the importance of taking trial product as directed.

Patients are also instructed to bring their diary to the visits. Patients judged to have questionable compliance (defined as taking less than 80% or more than 120% of the study medication) will continue in the study, but should be counselled on the importance of taking their medication as prescribed.

5.7 Costs and reimbursement

The participants receive the study medication without any cost. They will also receive travel reimbursement. This amount is subject to tax. Patients withdrawing from the study will receive payment for the visits that have taken place. Travel reimbursement will also be paid in cases of screening failure.

5.8 Destruction of study medication

The patients return any unused or partly used study medication at their final visit. The remaining medication is counted and then destructed via Tamro AB. In case patients withdraw from the study prematurely and will not attend more visits to the study site the patients may also return their remaining medication to a pharmacy. In those cases we will contact the pharmacy and request a destruction note.

5.9 Treatment after study

All subjects should be assessed at end of study (Visit 3 or the latest visit for subjects who discontinue) concerning treatment effect. It should also be assessed if the subject would benefit from ongoing additional anti-diabetic medication in addition to metformin. Choice of treatment is based on current HbA1c, AEs and the patient's view and should then be prescribed for an adequate time by the Principal Investigator. In parallel, a formal letter of referral should be sent to the ordinary care giver at end of study describing the response to treatment and decisions made concerning future treatment until the responsible care giver once again has reasonable opportunity to resume responsibility for the patient. The Principal Investigator is responsible for the anti-diabetic treatment until the ordinary care giver has responded that s/he resumes responsibility.

In addition to the post-study anti-diabetic treatment as described above, if the patient at end of study is severely dysregulated in blood pressure or blood lipids, in the sense that blood pressure is above 140/90, total cholesterol ≥7.0 mM, LDL cholesterol ≥5.0 mM or triglycerides ≥5.6 mM, we will initiate rescue medication based on the considerations for treatment choice as described in 2.6 and 3.4, and send a referral to the appropriate responsible physician. The Principal Investigator is responsible for rescue medication until the ordinary care giver has responded that s/he takes responsibility for follow-up and further treatment.

6. STATISTICS

6.1 Statistical analyses

We will use both full analysis set and per protocol analysis of the outcome variables. The full analysis set will include all participants who have at least one value after randomization (whether from visit 2 or 3) independent of compliance. Participants who are withdrawn prematurely from the study and have taken their medication for at least 3 months will be asked to attend a study visit if possible to measure study variables. That study visit will if possible follow the procedures of Visit 3, including an OGTT. If data from visit 3 are not available, data from visit 2 will be used.

Participants with data of the primary variable from all study visits and least 80% compliance overall and >80% during the last month will be included in the per protocol analysis.

6.2 Demographics and baseline characteristics

Demographic and baseline characteristics will be summarized, using frequency distributions and summary statistics based on the full data set, for each treatment group as well as for all patients combined.

Additional summaries of demographic and baseline characteristics may be performed for specific subgroups, including participants with HbA1c at 48 mmol/mol or above.

6.3 Efficacy analyses

The primary endpoint will be the intraindividual change from baseline in HbA1c (ΔHbA1c in mmol/mol) in response to semaglutide or dapagliflozin in SIDD versus SIRD patients, which will be analysed using an ANCOVA model with a term for the exposure and an interaction term for the exposure and subgroup. HbA1c at baseline

will be used as a covariate. Thus, treatment, SIDD/SIRD assignment and the interaction will be analysed. In a supportive analysis we will also correct for metformin dose using a continuous measure of metformin dose (in mg). If the metformin dose, despite our instructions, has been changed during the study the participant will not be included in the PP analysis.

To better reflect the continuous nature of the disease variables, we will also include a measure of cluster centeredness, as individuals near the centre of a cluster, ‘archetypes’, are more likely to exhibit the typical characteristics of SIDD and SIRD than those at the periphery. We will therefore make a subanalysis based on HOMA-B, HOMA-IR, age, BMI and HbA1c (the variables used to cluster patients into SIDD and SIRD) to analyze how cluster centricity influences treatment efficacy.

The secondary variables will be analyzed similar to the primary efficacy variable. The secondary variables will be compared between between visit 1 and 3 to obtain intraindividual delta values for each participant. The intraindividual delta values will then be analysed across all subjects using independent t-tests.

Missing data will not be imputed.

6.4 Sample size

The standard deviation of ΔHbA1c is 4.9 mmol/mol over 6 months (as observed in the DIACT cohort). With 80% power at alpha=0.05, we need 43 SIDD and 43 SIRD patients to detect a significant treatment effect between the clusters, assuming that the true treatment effect is 3 mmol/mol.[11,12] This applies to both dapagliflozin and semaglutide We aim to recruit 50 individuals to each treatment arm, totally 100 SIDD and 100 SIRD patients. If a participant is withdrawn during the study and has taken the study medication for at least 3 months with 80-120% compliance, the participant will be asked to attend a final visit, which will then, if possible, follow the procedures outlined for Visit 3. If we have not obtained any outcome data for HbA1c for a patient, we will recruit new participants to get the required number of participants for the full analysis set.

7. DATA MANAGEMENT

7.1 Quality control, data and sample handling

All samples should be taken by adequately trained study personnel. The Investigator is to ensure that all personnel involved in the study is appropriately trained. All personnel in the study must have read the protocol in detail.

Routine blood samples will be analysed directly at the hospital's central laboratory. We calculate eGFR as the mean of the relative eGFR based on creatinine (using the revised Lund-Malmö estimating equation) and the relative eGFR based on Cystatin C (using the CAPA formula). Analyses of blood and urine metabolites and proteins from stored samples and stool samples will be made using special kits or equipment (e.g. mass spectrometry for metabolites, ELISA, RIA and arrays for proteins and sequencing for stool samples). These analyses aim to

- Analyse plasma c-peptide, proinsulin, glucagon and GLP-1 to assess beta-cell function and incretin levels in response to treatment in the different patient groups
- short-term identify biomarkers that distinguish SIDD and SIRD patients and predict drug responders and non-responders
- long-term, up to several years after the present study, predict disease deterioration and complications (see 7.3).

All data from CRF and routine blood analyses will be entered manually by specific study personnel into a secure database. Quality control procedures will be applied to each stage of data handling to ensure that all data are reliable and have been processed correctly.

Questionnaires will be given in paper format and the responses will be entered manually into the database by specific study personnel. Only study ID will be used to

identify individuals. The questionnaire data will be analysed using numeric variables and summed into subscales where appropriate.[6,7,16]

Data from glucose sensors will be transferred from the reader to a local disc (no cloud-based storage) as text files. Data from exploratory variables (metabolite, protein and gut microbiota analyses) will be in electronic format using study ID.

The database is stored at local servers at Clinical Research Center, Skåne University hospital, behind a firewall. Only authorized personnel can access the data and all logins are traceable. Only specific IT staff has physical access to the server facility. All stored data will be coded using study ID and analyses are done using coded data. Only the sponsor or by him assigned persons will have access to these pseudonymised data.

The study information and informed consent form describe how data are handled and the procedure if a subjects wants to view, correct or remove data.

Data and samples may also be shared with academic or industrial partners outside the University of Gothenburg or Region Skåne. In that case, any data or samples shared will be anonymized, i.e. the study ID will be removed so that data cannot be linked back the data to the personal ID.

7.2 Documentation / Case Report From

All study data collected in the study will be registered in a paper case report form (CRF). The participant is identified through his/her study ID.

All AE, SAE and SUSAR are classified by the investigator and noted in the CRF.

The investigator, sponsor, monitor and persons assigned by the investigator have access to the CRF.

Correction of CRF is done by manually changing the data, signing and dating the correction.

Data that are specific to the study and not relevant for the care of the participant do not have to be entered into the participant's medical record.

All data in the medical record must be in agreement with the CRF. The name of the study, date for informed consent, study medication, and date for end of treatment must be noted in the medical record. The participant's study ID does not have to be entered in the medical record as the study is not blinded.

CRF and the laboratory results (in paper form) constitute source data. A document of what is classified as source data will be in the Investigator File. The Investigator must ensure that all source data are accessible for monitoring and other quality control.

The investigator must keep a log of staff and a delegation of tasks list at site.

7.3 Data handling after study

Study document and source data will be archived for at least 10 years after study report. Source data in the hospital's medical records system will be stored according to

local regulations.

Data will be published in peer-reviewed medical journals, at scientific conferences, for lay audience and in media and updated in the Eudra-CT database and clinicaltrials.gov within 1 year after end of trial. Only de-identified data based on averages will be used. Data from exploratory analyses may be published at a later stage than the report(s) describing the primary and secondary variables.

The protocol may be published in part or full in accordance with scientific standard.

Study participants may after last visit get to know their routine lab data collected during the study, including Hba1c, if so requested. The sponsor has however no obligation to report other study data, including e.g. analyses of blood metabolomics or gut microbiota, to individual participants. The study participants will after publication of the results receive a brief summary of the study outcome for their information.

The ANDIS registry is continuously updated with data on diabetic complications, medication and diabetes-relevant blood variables. Since all participants will also be part of ANDIS this opens up a valuable opportunity to verify whether blood, urine or stool biomarkers collected in the study can predict long-term disease outcomes. We will therefore after the study prospectively request information from ANDIS on HbA1c, complications (retinopathy, nephropathy, neuropathy and cardiovascular diagnoses) as well as current diabetic medication (with a particular focus on those remaining on semaglutide/dapagliflozin). The aim of this is to identify biomarkers that can predict which patients 1) have increased risk for deteriorated glucose control, 2) have increased risk for specific complications and 3) respond best to semaglutide/dapagliflozin over time in case they continue using those compounds (see also 7.1). We will inform the participants that their study data will be compared with long-term disease outcomes in the ANDIS registry. They can at any time withdraw their consent to that. In that case, no prospective analyses will be done for that participant.

7.4 Monitoration

The study will be monitored by an independent monitor before the study begins, during the study conduct, and after the study has been completed, to ensure that the study is carried out according to the protocol and that data is collected, documented, and reported according to ICH-GCP and applicable ethical and regulatory requirements. Monitoring is performed as per the study's monitoring plan and is intended to ensure that the subject's rights, safety, and well-being are met as well as data in the CRF are complete, correct, and consistent with the source data.

Failure to follow the protocol, GCP or other regulations in a way that significantly affects or likely could affect the participants or the scientific value of the study should be reported to the Medical Product Agency within 7 days. It is the responsibility of the sponsor to make this decision. Smaller aberrations that do not affect the integrity or security of the participants or the scientific value of the study should be documented by the sponsor/investigator.

It is the responsibility of the Investigator to ensure that the monitor has access to the CRF, the medical record and original laboratory data etc. to ensure that source data are

relevant, without violating the integrity of the participants. The sponsor and the monitor will make a risk-based monitoring plan and the sponsor will continuously follow up the plan. An agreement is signed before monitoration to ensure that the monitor is not disclosing personal data about the participants to any third party.

Authorized representatives of a regulatory authority may perform inspections at the center. The purpose of an inspection is to systematically and independently examine all study-related activities and documents, to determine whether these activities were conducted, and data were recorded, analyzed, and accurately reported according to the protocol, ICH GCP guidelines and any applicable regulatory requirements.

Major protocol changes are only possible by approved amendments to the ethics committee and/or the Medical Products Agency. Changes should be marked carefully and protocol version updated accordingly.

8. HANDLING OF ADVERSE EVENTS

8.1 Definitions

An **adverse event** (AE) is any untoward medical occurrence in a subject administered a medicinal product, and which does not necessarily have a causal relationship with this treatment.

An AE can therefore be any unfavourable and unintended sign (including an abnormal laboratory finding), symptom or disease temporally associated with the use of a product, whether or not considered related to the product.

An AE includes:

- A clinically significant worsening of a concomitant illness.
- A clinical laboratory abnormality which is clinically significant, i.e. an abnormality that suggests a disease and/or organ toxicity and is of a severity that requires active management. Active management includes active treatment or further investigations, for example change of medicine dose or more frequent follow-up due to the abnormality.

The following should not be reported as AEs:

- Pre-existing conditions, including those found as a result of screening or other trial procedures performed before exposure to trial product (pre-existing conditions should be reported as medical history or concomitant illness).
- Pre-planned procedures unless the condition for which the procedure was planned has worsened from the first trial related activity after the subject has signed the informed consent.

A **serious adverse event** (SAE) is an experience that at any dose results in any of the following:

- Death.
- A life-threatening experience.
- In-patient hospitalisation or prolongation of existing hospitalisation.
- A persistent or significant disability or incapacity.
- Congenital anomaly/birth defect
- Important medical events that may not result in death, be life threatening or require hospitalisation may be considered an SAE when - based on appropriate medical judgement - they may jeopardise the subject and may require medical or surgical intervention to prevent one of the outcomes listed in the definition of SAE.

A **Suspected Unexpected Serious Adverse Reaction** (SUSAR) is an SAE that has not been documented or reported in previous studies with the drugs. All SAEs must be evaluated as to whether they are unexpectedly or expectedly related to the drug or not at all related.

8.2 Reporting and documentation of adverse events

Adverse Events will be collected from the signing of informed consent throughout the study until and including the last visit/contact.

Adverse events will be collected through spontaneously reporting and by telephone contact in between visits as well as by questions from study personnel at visits followed by documentation in the CRF, including intensity and causality. AEs identified during the study will be followed up by visits to the study site for blood sampling and clinical examination, telephone contacts or appropriate referrals based on the overall clinical picture. AEs should be followed up until the patient has recovered or is taken care of by other healthcare clinics.

Any unresolved AE during the study or at end of study (at visit 3 or earlier in case of withdrawal) will be followed up by up by the Investigator for as long as medically indicated. This will include visits and the study site for blood sampling and clinical examination, telephone contacts and/or appropriate referrals based on the overall clinical picture. Adverse events should be followed up until resolved or stabilized.

Any aberrant blood values discovered at the screening visit will not be considered as AEs and will be handled as appropriate, also for individuals who are not included.

Any change to a concomitant illness should be recorded during the trial. A clinically significant worsening of a concomitant illness must be reported as an AE

The following variables will be collected for each AE:

- AE (verbatim)
- Date of start and stop

- Maximum intensity
- Whether the AE is serious or not
- Causal relationship with study drug, graded as likely, possible or unrelated
- Action
- Outcome (resolved / not resolved)

In addition, the following variables will be collected for SAEs:

- Date AE met criteria for serious AE
- Date Investigator became aware of serious AE
- AE is serious due to
- Date of hospitalization
- Date of discharge
- Detailed description of AE.

Intensity will be graded according to the following rating scale:

• **Mild** (awareness of event but easily tolerated)

• **Moderate** (discomfort enough to cause some interference with usual activity)

• **Severe** (inability to carry out usual activity)

Causality is graded by the investigator as follows:

- **Likely related**, occurring within a reasonable time after administration of the intervention. It is unlikely that the event can be attributed to underlying disease or other drugs without it most likely being caused by the trial drug and its occurrence being reasonable in connection with the use of the trial drug.
- **Possibly related**, occurring within a reasonable time after administration of the intervention. The event may be explained by the trial drug and the onset is reasonable in connection with the use of the trial drug, but there is insufficient information to establish the relationship. The event can be explained by underlying disease or other drugs.
- **Unrelated**, i.e. unlikely to be related to the intervention and may be explained by other drugs or underlying disease.

Deterioration as compared to baseline or reference values in clinical variables measured as part of the protocol should only be reported as AEs if they fulfill any of the SAE criteria or are the reason for discontinuation of treatment with the study medication.

If deterioration in clinical variables is associated with clinical signs and symptoms, the sign or symptom will be reported as an AE and the associated laboratory result/vital

sign will be considered as additional information.

Hyperglycemia is not considered an AE.

Hypoglycemic episodes or symptoms of hypoglycemia should only be reported in the CRF if the event fulfills the definition of a major hypoglycemic event (requiring external assistance due to severe impairment in consciousness or behavior) or the protocol criteria for an SAE.

Dapagliflozin has a modest diuretic effect. Therefore, caution should be exercised when administering dapagliflozin to patients at risk for volume depletion due to co-existing conditions or concomitant medications, such as loop diuretics. These patients should be carefully monitored for volume status, electrolytes, and renal function.

In patients with clinical evidence of upper urinary tract infection (e.g., pyelonephritis) or urosepsis, the Investigator may consider temporarily stopping dapagliflozin treatment until the course of treatment of the infection has been completed and the patient has recovered. That is typically not necessary for lower urinary tract infections, which should be handled according to clinical routines.

Overdose is defined as the accidental or intentional ingestion of any dose of investigational product that is considered both excessive and medically important. Once the Investigator decides that a particular occurrence is an overdose, it must be reported as an SAE and proper action taken, including necessary healthcare contacts or referrals to emergency unit.

If a patient becomes pregnant during the course of the study investigational product should be discontinued immediately. Pregnancy itself is not regarded as an adverse event unless there is a suspicion that the investigational product under study may have interfered with the effectiveness of a contraceptive medication.

SAE should be reported by the study staff to the investigator/sponsor (A. Rosengren) who completes an SAE document within 24 h of becoming informed of the SAE. The investigator/sponsor evaluates causality.

All SAEs related to Forxiga will also be submitted by the investigator/sponsor to the AstraZeneca Product Safety mailbox: AEMailboxClinicalTrialTCS@astrazeneca.com

SUSAR that are lethal or life-threatening are to be reported to the Medical Product Agency by the sponsor as soon as possible and latest 7 days after the sponsor was being informed using a CIOMS document. Other SUSARs are to be reported as soon as possible and latest within 15 days after the sponsor being informed. The Medical Product Agency will help us to report SUSAR in EUDRA-vigilance.

A Development Safety Update Report will be sent to the Medical Product Agency annually.

9. ETHICAL ASPECTS

9.1 Risks - benefits

The study will be done in accordance with applicable laws and regulation and the principles of GCP and the Helsinki Declaration.

The invasive procedures are the capillary and venous cannulations, which may give local pain. The patients also carry a continuous glucose sensor, which means that a fine needle is placed subcutaneously. Those sensors are usually tolerated well. In cases of erythema or other problems with the sensor, the patient may contact us and may remove the sensor if it causes unacceptable problems.

In case venous samples cannot be obtained, we may analyse Hba1c and glucose via capillary samples at Visit 1 and 3. We do not see that this affects the risk-benefit assessment.

We will recruit participants with HbA1c ≥42 and <91 mmol/mol. We will introduce rescue medication in case patients get too high HbA1c, and we do not see any risks with the chosen HbA1c interval for recruited participants.

Both study drugs are approved for T2D at the provided doses. The drugs may be associated with AEs, but relevant precautions have been implemented in the design and planned conduct of the trial in order to minimise the risks and inconveniences of participation in the trial. These precautions include assessment of safety variables, information regarding the correct administration of the drugs, gradual dose adjustment for semaglutide as well as appropriate exclusion and withdrawal criteria. If the interval between screening visit and visit 1 is beyond 2 months, then safety variables will be re-analysed (see 3.2).

The most common adverse reactions reported with dapagliflozin are hypoglycaemia (when used with sulphonylurea or insulin which is not relevant here), genital infections, pruritus (generalised) and increased urination. Uncommon adverse reactions are volume depletion and dysuria. There is a risk for diabetic ketoacidosis in patients treated with dapagliflozin. In the DECLARE study, 8574 patients received

Forxiga 10 mg and 8569 patients received placebo for a median exposure time of 48 months. Events of DKA were reported in 27 patients in the Forxiga 10 mg group and 12 patients in the placebo group. The events occurred evenly distributed over the study period. Of the 27 patients with DKA events in the FORXIGA group, 22 had concomitant insulin treatment at the time of the event. Precipitating factors for DKA were as expected in a type 2 diabetes mellitus population.

The study participants will receive written and oral information on the signs and symptoms of DKA and are instructed to contact healthcare immediately if DKA is suspected. As a safety measure, subjects with a history of diabetic ketoacidosis will be excluded from this trial. If ketoacidosis is suspected, Forxiga will be discontinued and treatment instituted.

The most commonly reported side effects of semaglutide are gastrointestinal (nausea, diarrhoea and vomiting), typically mild to moderate and of short duration. We will apply a dose escalation regimen to minimize these side effects. Acute pancreatitis has been reported in subjects treated with GLP-1 receptor agonists. As a precaution, subjects with a history of acute or chronic pancreatitis will not be enrolled in the trial. Also, subjects will be informed about the symptoms of acute pancreatitis.

There are several benefits for the participants. They get a better characterization of their disease and general health and we will follow up medically relevant abnormal values. Treatment with study medication is expected to provide clinically relevant improvements in glycaemic control. It is also expected that the subjects will benefit from participation through the contacts with the study site, with close follow-up of their T2D and a careful medical examination, all of which will most likely result in an intensified management. All subjects in this trial will receive trial products and auxiliary supplies free of charge.

Improved knowledge of which patients benefit most from these clinically approved drugs could open up for more specific and effective treatment in a rather near future, which could benefit both the participants and T2D patients in general.[13-15]

In conclusion, the potential risk to the subjects in this trial is considered low and acceptable in view of the anticipated benefits the study medication will provide to participants.

Risk/benefit assessment in relation to the Covid-19 epidemic

In light of the current Covid-19 epidemic the sponsor should continuously reassess the overall risk and feasibility to conduct the study as the situation develops. This reassessment should be documented. The sponsor should continue safety reporting in adherence to EU and national legal frameworks and should be particularly attentive to reporting of symptoms or signs that could potentially be related to Covid-19.

The Investigator should continuously assess the risk for each participant, based on age, underlying diseases and social situation, and implement measures which prioritize subject safety. Participation for an individual subject may be postponed or inhibited if the Investigator finds it unsafe or otherwise inappropriate to participate. It should be emphasized that subjects have their right to withdraw at any time.

The participants will be instructed to adhere to updated general national recommendations, e.g. not attend the study site in case of infectious symptoms.

It will be ensured that there is an appropriate stock of study material, blood glucose devices and IMP. Any protocol deviations because of Covid-19-related issues should be documented carefully. Actions and substantial changes to the overall assessment should be communicated to the MPA and Ethics committee.

There is a risk that subjects become exposed to Covid-19 by attending study visits, but we do not see that these visits should lead to higher risk of exposure compared to other public places or hospital areas since there is no reason to believe that there is an overrepresentation of SARS-CoV-2 carriers at the study site. The risk is likely lower compared with e.g. waiting rooms at primary care centres.

The IMP is in this case targeting the subjects' diabetic condition (as compared with trials using IMPs testing research questions unrelated to the subjects' underlying disease). Both IMPs are likely to lower the subjects' blood glucose and we have no placebo group. Improved control and management of a chronic condition like diabetes is likely to be beneficial to the subjects during the current circumstances by improving their overall health status and ability to cope with serious infections. The visits will also lead to assessment and better management of other factors of relevance for Covid-19 risk, including elevated blood pressure.

Based on the current risk/benefit evaluation the study is considered feasible to conduct. The evaluation will however be continuously reassessed.

9.2 Study information and ethical permits

The participants must give written informed consent before included in the study. The original of the consent form will be stored with the Investigator and the participants will receive a copy.

The participants will receive objective and neutral information about the study both in the invitation letter and at the study visits.

The study may start when there are approvals from the Ethical committee, the MPA and the biobank.

Substantial changes should be approved as Amendments by the Ethical committee and/or the MPA.

All correspondence with the Ethical committee and MPA should be saved.

The study will be reported at clinicaltrials.gov.

The Principal Investigator will:

- Ensure each patient is given full and adequate oral and written information about the nature, purpose, possible risk and benefit of the study
- Ensure each patient is notified that they are free to discontinue from the study

at any time

- Ensure that each patient is given the opportunity to ask questions and consider the information provided
- Ensure each patient provides signed and dated informed consent before conducting any procedure
- Ensure the original, signed informed consent form is stored in the Investigator’s Study File and a copy is given to the patient

Leaving stool samples is optional, and the patient can at any time freely discontinue that component of the study and yet continue with the other parts of the protocol.

9.3 Insurance

The participants are insured through Patientskadelagen and Läkemedelsförsäkringen.

10. REFERENCES

1. Worldwide trends in diabetes since 1980. The Lancet, 2016. 387(10027): p. 1513-1530.
2. Diabetes Prevention Program Research, The Lancet. 374(9702): p. 1677-1686.
3. Davies MJ et al., Diabetologia (2018) 61:2461–2498
4. Ahlqvist et al., Lancet Diabetes and Endocrinology. 2018 March 2
5. Al Jobori H et al., J Clin Endocrinol Metab. 2018 Apr 1;103(4):1402-1407.
6. Bradley C (1994) The Diabetes Treatment Satisfaction Questionnaire: DTSQ. In Bradley C (Ed) (1994) Handbook of Psychology and Diabetes: a guide to psychological measurement in diabetes research and practice.
7. Dalton M et al., European Journal of Clinical Nutrition (2015) 69, 1313–1317
8. H. Wu et al., Nature Medicine 23, 850–858 (2017)
9. Pratley RE et al., Lancet Diabetes Endocrinol. 2018 Apr;6(4):275-286.
10. Wiviott SD et al., N Engl J Med 2019;379:347-57.
11. A. J. Garber, et al., Am. J. Med. 103, 491–497 (1997).
12. R. Arechavaleta, et al., Diabetes Obes Metab 13, 160-168 (2011)
13. McCarthy M. Diabetologia (2017) 60:793–799
14. Pearson E. 2013. Diab Med.31,393-98

15. Fitipaldi H. et al., Diabetes 2018;67:1911–1922

16. Rodbard HW et al., Diabetes Care 2019;42:2272–2281

STATISTICAL ANALYSIS PLAN

Statistical analyses

We will use both full analysis set and per protocol analysis of the outcome variables. The full analysis set will include all participants who have at least one value after randomization (whether from visit 2 or 3) independent of compliance. Participants who are withdrawn prematurely from the study and have taken their medication for at least 3 months will be asked to attend a study visit if possible to measure study variables. That study visit will if possible follow the procedures of Visit 3, including an OGTT. If data from visit 3 are not available, data from visit 2 will be used.

Participants with data of the primary variable from all study visits and least 80% compliance overall and >80% during the last month will be included in the per protocol analysis.

Demographics and baseline characteristics

Demographic and baseline characteristics will be summarized, using frequency distributions and summary statistics based on the full data set, for each treatment group as well as for all patients combined.

Additional summaries of demographic and baseline characteristics may be performed for specific subgroups, including participants with HbA1c at 48 mmol/mol or above.

Efficacy analyses

The primary endpoint will be the intraindividual change from baseline in HbA1c (ΔHbA1c in mmol/mol) in response to semaglutide or dapagliflozin in SIDD versus SIRD patients, which will be analysed using an ANCOVA model with a term for the exposure and an interaction term for the exposure and subgroup. HbA1c at baseline will be used as a covariate. Thus, treatment, SIDD/SIRD assignment and the interaction will be analysed. In a supportive analysis we will also correct for metformin dose using a continuous measure of metformin dose (in mg). If the metformin dose, despite our instructions, has been changed during the study the participant will not be included in the PP analysis.

To better reflect the continuous nature of the disease variables, we will also include a measure of cluster centeredness, as individuals near the centre of a cluster, 'archetypes', are more likely to exhibit the typical characteristics of SIDD and SIRD than those at the periphery. We will therefore make a subanalysis based on HOMA-B, HOMA-IR, age, BMI and HbA1c (the variables used to cluster patients into SIDD and SIRD) to analyze how cluster centricity influences treatment efficacy.

The secondary variables will be analyzed similar to the primary efficacy variable. The secondary variables will be compared between between visit 1 and 3 to obtain intraindividual delta values for each participant. The intraindividual delta values will then be analysed across all subjects using independent t-tests.

Missing data will not be imputed.

Sample size

The standard deviation of ΔHbA1c is 4.9 mmol/mol over 6 months (as observed in the DIACT cohort). With 80% power at alpha=0.05, we need 43 SIDD and 43 SIRD patients to detect a significant treatment effect between the clusters, assuming that the true treatment effect is 3 mmol/mol.[11,12] This applies to both dapagliflozin and semaglutide We aim to recruit 50 individuals to each treatment arm, totally 100 SIDD and 100 SIRD patients. If a participant is withdrawn during the study and has taken the study medication for at least 3 months with 80-120% compliance, the participant will be asked to attend a final visit, which will then, if possible, follow the procedures outlined for Visit 3. If we have not obtained any outcome data for HbA1c for a patient, we will recruit new participants to get the required number of participants for the full analysis set.
